# Universality, Limits and Predictability of Gold-Medal Performances at the Olympic Games

**DOI:** 10.1371/journal.pone.0040335

**Published:** 2012-07-12

**Authors:** Filippo Radicchi

**Affiliations:** Departament d’Enginyeria Quimica, Universitat Rovira i Virgili, Tarragona, Spain; University of Namur, Belgium

## Abstract

Inspired by the Games held in ancient Greece, modern Olympics represent the world’s largest pageant of athletic skill and competitive spirit. Performances of athletes at the Olympic Games mirror, since 1896, human potentialities in sports, and thus provide an optimal source of information for studying the evolution of sport achievements and predicting the limits that athletes can reach. Unfortunately, the models introduced so far for the description of athlete performances at the Olympics are either sophisticated or unrealistic, and more importantly, do not provide a unified theory for sport performances. Here, we address this issue by showing that relative performance improvements of medal winners at the Olympics are normally distributed, implying that the evolution of performance values can be described in good approximation as an exponential approach to an *a priori* unknown limiting performance value. This law holds for all specialties in athletics–including running, jumping, and throwing–and swimming. We present a self-consistent method, based on normality hypothesis testing, able to predict limiting performance values in all specialties. We further quantify the most likely years in which athletes will breach challenging performance walls in running, jumping, throwing, and swimming events, as well as the probability that new world records will be established at the next edition of the Olympic Games.

## Introduction

Modern Olympics are inspired by the ancient version of the Games, but based on a wider idea of globality. While ancient Games were opened only to Greek speaking athletes [1], modern Olympics were, since their beginning, considered a world event involving people from every part of the globe [2]. The same symbol of the Olympics, composed of five interlocking rings standing for the five continents, was designed by the *Baron Pierre de Coubertin*, the founder of the modern Olympic Games, with the aim of reinforcing the idea that the Games are an international event and welcome all countries of the world [3]. Since Athens 1896, 26 editions of the event has been organized in different locations around the world, and, from the 241 participants representing 14 nations of the first edition, the Games have grown to about 10,500 competitors from 204 countries at the latest edition of the summer Games of Beijing 2008. The Olympics are one the most important events worldwide not only for sports, but also for politics and society. Many important facts of the last century history, such as the Nazism [4], the Israeli-Palestinian conflict [5], and the cold war [6], have influenced the regular organization of the Games. Also, the Olympics generally play a fundamental and positive role for the economic and urban development of the city that hosts the event [7], [8].

Performance data of athletes at the Olympics are available for each modern edition of the Games organized so far, and represent an optimal proxy for the study of human limits in sport performances for three main reasons: (i) Data cover more than a century of sport performances since the first edition of the Olympics dates back to 1896; (ii) Olympic data provide a detailed record of sports performances at regular 4-year intervals; (iii) The performances of Olympic medalists truly reflect the best achievements that could be obtained in a given historic moment because, in the vast majority of sport disciplines, the Games have always represented the most important event during the career of an athlete, and consequently all the greatest athletes have always taken part in the Olympics.

Latest years have witnessed the appearance of a large number of statistical studies of data coming from professional sports. Examples include basketball [9], [10], baseball [11]–[15], soccer [16], tennis [17], etc. Also Olympic performance data have been the subject of many analyses [18]–[28]. Some of them focused on models aimed at the description of performance progression along time, including linear models [24] that can even lead to unrealistic results [29], [30], S-shaped curves [25] and logistic functions [27]. Others studied statistical properties of performance patterns, such as the power-law relation between time (or speed) and length of running events [19], [21], [22]. In addition, performance data of athletes at the Olympics have been used to tune the parameters of complicated models aimed at the determination of physiological limits in sport performances [31]–[33]. For example, according to a mathematical model for human running performance that accounts for various energetic factors, such as capacity of anaerobic metabolism, maximal aerobic power and reduction in peak aerobic power, Perronet and Thibault predicted the limiting times that athletes can reach in various running events in athletics [32].

In spite of the numerous efforts however, we still miss a general description for the performances of athletes. We still miss a universal way to predict limiting performance values and calculate the probability of future achievements in sport. In this paper, we address all these issues by generating a simple and coherent picture for the description of the performances obtained by Olympic medal winners in all specialties of athletics and swimming. We analyze historic performance data and provide empirical evidence about the discovery of a novel statistical law governing performances of medal winners at the Olympic Games. With a self-consistent approach we simultaneously (i) show that performance improvements obey a universal law, (ii) estimate limiting performance values, (iii) predict future achievements at the Olympics.

## Results

While former statistical studies have mainly analyzed the progression of absolute performance values along the various editions of the Games, here we change point of view and focus our attention on relative improvements in performances between two consecutive editions of the Olympics. Let us indicate with 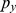 the value of the performance obtained by the gold medalist in a specific specialty at the edition of year *y* of the Olympic Games. Depending on the specialty, 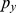 may indicate time (running and swimming), length (long and triple jumps), height (high jump and pole vault), or distance (discus and hammer throws, shot put). We define the relative improvement of the gold-medal performance in the Games of year *y* with respect to the gold-medal performance in the previous edition of the Olympics as
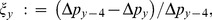
(1)where 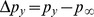 represents the gap between the performance value of the gold medalist in year *y* and the asymptotic performance value 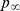. The asymptotic or limiting performance value 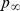 is a unknown parameter representing the physiological limit that can be achieved in the specialty by an athlete. Eq. 1 defines the relative improvement towards the asymptotic performance value of the gold medalist in year *y* with respect to the performance of the gold medalist in year 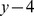. Note that the same definition can be used for the measurement of the relative improvements of silver and bronze medalists, and in principle for athletes who have reached any arbitrary rank position.

**Figure 1:**
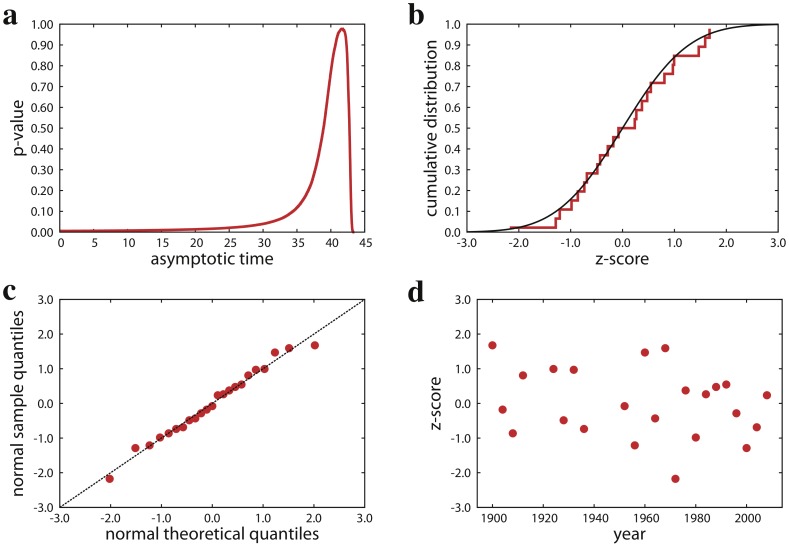
Performances of male gold medalists in 400 meters sprint. **a.** Best estimate of the asymptotic performance value. For each value of 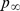 lower than the actual Olympic record, we evaluate the goodness of the fit of performance improvements with a normal distribution. 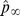 is determined as the value of the asymptotic time 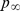 that maximizes the statistical significance (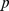-value). For men 400 meters sprint, our best estimate is 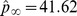 seconds, where we find that relative performance improvements are normally distributed with a confidence of 98%. For this value of 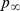, the best empirical estimates of the average value and standard deviation are respectively 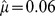 and 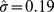. **b.** The cumulative distribution function of the 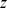-scores obtained for 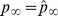 (red curve) is compared with the standard normal cumulative distribution (black curve). **c.** Normal sample quantile are plotted against normal theoretical quantiles [51]. The dashed line corresponds to the theoretically expected behavior in case of a perfect agreement between sample and theoretical distributions. **d.** 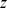-scores of relative performance improvements between consecutive editions of the Games.

For reasonable values of 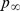, we find that the distribution of the relative performance improvements is statistically consistent with a normal distribution. We determine the best estimate of the asymptotic performance value 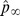 as the value of 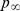 for which the statistical significance (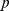-value) of the normal fit is maximized (see Materials and Methods section). The procedure is generally accurate and allows us to identify reasonable values of 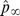 in all specialties considered in this study. In Fig. 1 for example, we report the results obtained by analyzing performance data of male athletes in 400 meters sprint. The best estimate of the asymptotic time is 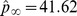 seconds. For this value of 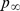, we find that relative improvements obey a normal distribution with average value 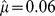 and standard deviation 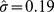. Statistical significance, however, can be used not only for the determination of the best estimate of the asymptotic performance value, but also, in a broader sense, to define confidence intervals for 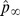. In the case of 400 meters sprint of male athletes for example, we find that, at 5% significance level, 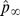 is in the range 31.03 to 43.09 seconds. At 50% significance level, the interval is restricted and 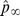 is in the range 38.91 to 42.74 seconds, while, at 95% significance level, 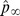 is expected to be between 41.04 and 42.13 seconds. The results shown in Fig. 1 are obtained by analyzing the relative performance improvements of gold-medal winners. Similar results are, however, obtained when considering the performances of silver and bronze medal medalists (Fig. S1). Interestingly, the finiteness of the data does not affect the reliability of the best estimate of the limiting performance value since compatible values of 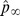 can be detected by removing results of the latest editions of the Games from the analysis (Fig. S2).

**Figure 2:**
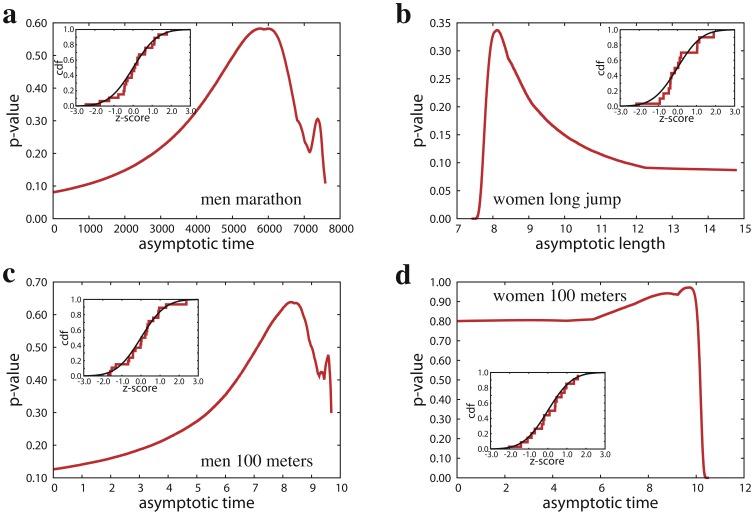
Statistical properties of performance improvements in athletics. In the main panels we show the determination of the best estimate 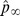 of the asymptotic performance value, while in the insets we provide a graphical comparison between the sample cumulative distributions (red line) and the standard normal cumulative distribution (black line). **a** and **b.** We report the results obtained by the analysis of the performances of male athletes in marathon (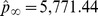 seconds, 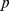-value 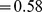) and female athletes in long jump (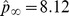 meters, 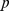-value 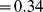). **c** and **d.** We show the outcome of our method for performances of men and women in 100 meters sprint (respectively, 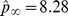 seconds and 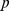-value 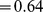, 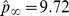 seconds and 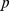-value 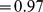).

The normality of the relative improvements towards the asymptotic performance value is a simple and strong result. At each new edition of the Games, gold-medal performances get, on average, closer to the limiting performance value. The average positive improvement observed in historic performance data can be motivated by several factors: as time goes on, athletes are becoming more professionals, better trained, and during the season have more events to participate in; the pool for the selection of athletes grows with time, and, consequently there is a higher level of competition; the evolution of technical materials favors better performances. On the other hand, there is also a non null probability that winning performances become worse than those obtained in the previous edition of the Games (i.e., relative improvement values are negative). All these possibilities are described by a Gaussian distribution that accounts for various, in principle hardly quantifiable, factors that may influence athlete performances: meteorological and geographical conditions, athletic skills and physical condition of the participants, etc. The accuracy of the normal fit is not only testified by its high statistical significance, but also by graphical comparisons between the sample distribution and the theoretical normal distribution (see Figs. 1b and c). It is also important to note that the values of the relative improvements do not depend on the particular edition of the Games, and thus their distribution is stationary (Fig. 1d). The strength of our results, however, is not only in the significance of the fits, but especially in its generality. We repeated the same type of analysis for a total of 55 different specialties, and found that performance improvements are governed by a universal law. First of all, the law holds for all running events in athletics. This is valid for an heterogeneous set of running distances ranging from 100 to 42,195 meters (marathon, Fig. 2 and Supporting Information S1). Second, our analysis suggests that relative improvements are normally distributed not only when considering time performances, but also performances regarding length or height (jumps) and distance (throws). In Fig.2b for example, we report the outcome of our method when applied to performance data of female gold medalists in long jump. Other examples can be found in Supporting Information S2. Finally, the law is valid for performance improvements of athletes in swimming specialties (Supporting Information S3).

Given the attention received in the recent past [24], [29], [30], we reserve a special consideration to the comparison in performances between female and male athletes in 100 meters sprint. In Fig. 2c and 2d, we report the results obtained through the analysis of Olympic performances in this specialty. According to our analysis, the best estimate of the limiting time for males is 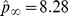 seconds, while for females we identify the best estimate for the asymptotic time at 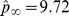 seconds. Our statistical analysis predicts that women will be always slower than men and that the gap will saturate at about 14%, consistent with the estimation by Sparling *et al* [20] but in disagreement with what predicted by the unrealistic model of Atkinson *et al* [24]. It should be noted that for women the statistical significance is less predictive than the one measured for men. While for men we observe that statistical significance is clearly peaked around 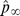 and goes rapidly to zero as 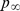 decreases, the same does not happen in the case of women. We believe that the statistics are less accurate because the analysis is based on 19 editions instead of 26 since women started to run the 100 meters sprint only in Amsterdam 1928, while men already in Athens 1896. In particular, the lack of sufficient data provides high statistical significance also for the unrealistic 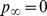 seconds. We expect, however, that the future addition of more data point will suppress this effect. Despite these problems, our analysis still produces meaningful estimates of the upper bound of the asymptotic time: at 5% significance level, the asymptotic value is expected to be lower than 10.31 seconds, while at 50% significance level, 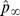 should be lower than 10.17 seconds. Also, our best estimates of the limiting performance values are probably not as accurate for this specialty (or other short distances) because there is not enough reliable performance data regarding the first editions of the Games (automatic time was introduced in Mexico City 1968). The removal of data points for male 100 meters sprint before Amsterdam 1928 (and in general of a few data points from the entire time serie) leads also to the impossibility to determine the best estimate of the asymptotic time as a global maximum of statistical significance (see Fig. S3). For 100 meters sprint, we have performed therefore an additional analysis in which we aggregated together the results of gold, silver and bronze medalists and obtained slightly different estimates for the limiting performance values [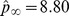 seconds for men (Fig. S4) and 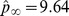 seconds for women (Fig. S5, S6)].

In general, our approach produces good results for specialties with a sufficiently long tradition in the Games. This is basically the case of all male specialties in athletics. Data about female performances typically provide less accurate results, but still, in the majority of the cases, the predictions of the asymptotic performance values are reasonable. We summarize in Table 1 the results obtained for some specialties, while we refer to the Supporting Information for a systematic analysis of all of them. It should be noted that there are also a few cases in which things do not work perfectly. In women 800 meters, for example, statistical significance does not exhibit any peak value (Supporting Information S1). There are also a few specialties in which the best estimate of the limiting performance value does not correspond to the global maximum of statistical significance (Supporting Information S1). In these cases, statistical significance is a non monotonic function of the 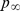 and more maxima are present. Still the peak value that appears more plausible can be used as an estimate of 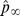. Finally, there are three specialties in athletics in which a clear peak in statistical significance is visible only by excluding performance data of Sidney 2000, but this exclusion is fully justified by the fact that the top athletes of the moment did not take part in the competition (Supporting Information S1). For example, about the men 200 meters sprint of Sidney 2000, the web site sports-reference.com reports: “This race was expected to be between the Americans *Maurice Greene* and *Michael Johnson*. Greene was the best in the world at 100 meters and Johnson at 400 meters, and their race in the middle distance was highly anticipated. But neither qualified for the team at the Olympic Trials, succumbing to minor injuries, although they both made the team in their better events.”

**Table 1:** Predictions of gold-medal performances in athletics and swimming.

sport	gender	specialty	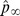	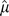	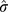	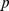 -value	*E*	*P*	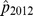
Track & Field	Men	100 m	8.28	0.04	0.10	0.64	26	0.35	9.63 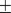 0.13
		110 m hurdles	11.76	0.05	0.12	0.48	26	0.50	12.87 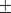 0.14
		400 m	41.62	0.06	0.19	0.98	26	0.14	43.62 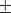 0.41
		10,000 m	1,539	0.05	0.19	0.45	22	0.01	1,617 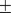 15
		marathon	5,771	0.03	0.15	0.58	26	0.34	7,537 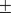 273
		pole vault	6.87	0.05	0.08	0.91	26	0.03	6.00 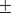 0.07
		hammer throw	103.81	0.04	0.09	0.47	25	0.03	82.89 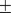 1.96
	Women	100 m	9.72	0.05	0.19	0.97	19	0.12	10.73 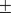 0.20
		400 m	45.14	0.02	0.15	0.77	12	0.00	49.53 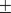 0.67
		long jump	8.12	0.04	0.18	0.34	16	0.01	7.08 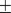 0.19
Swimming	Men	100 m fs	44.84	0.09	0.10	0.92	23	0.36	47.00 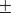 0.24
		100 m bs	48.98	0.09	0.11	0.93	22	0.24	52.22 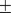 0.39
		100 m brs	57.38	0.16	0.16	0.93	11	0.36	58.67 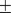 0.24
		1,500 m fs	577	0.05	0.05	0.50	23	0.71	866 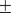 15
	Women	100 m fs	51.87	0.12	0.19	0.54	22	0.00	52.97 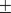 0.24
		100 m bs	54.73	0.08	0.14	0.59	20	0.20	58.62 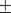 0.59
		100 m brs	62.08	0.13	0.10	0.86	11	0.15	64.77 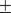 0.31
		800 m fs	388	0.05	0.07	0.84	11	0.76	489 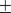 7

We summarize here some of the results obtained with our analysis. We list several specialties in athletics and swimming performed by male and female athletes. For each specialty, we report from left to right: the name of the specialty, the best estimates of the asymptotic performance value 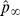, the best estimate of the mean value 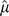, the best estimate of the standard deviation 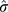, the statistical significance or 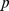-value of the test of normality, the number *E* of Olympic Games that included the specialty, the probability *P* that the actual world record will be beaten in London 2012, and the most likely performance value 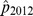 that gold-medal winners will obtain at the next edition of the Olympic Games. For shortness of notation, in swimming specialties we abbreviate “freestyle” with “fs”, “backstroke” with “bs”, and “breaststroke” with “brs”. The values of 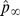 and 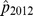 are reported in seconds for running and swimming races, and in meters for jumping and throwing events.

**Figure 3:**
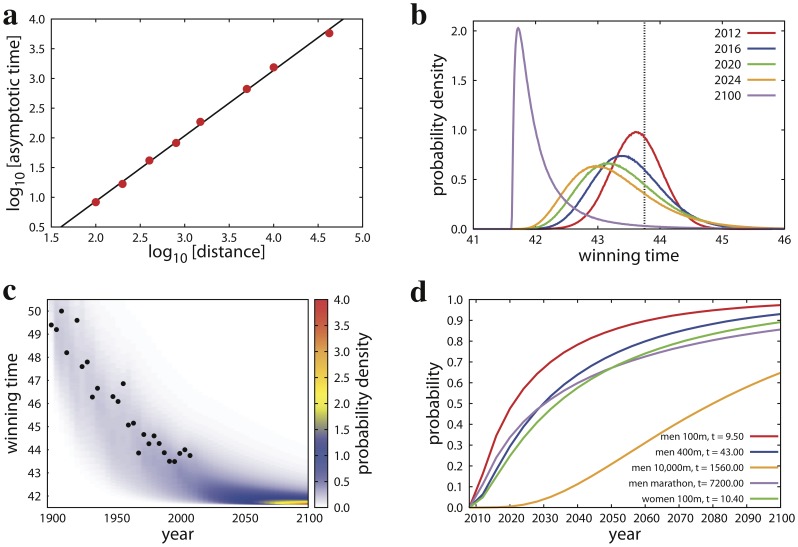
Scaling law between asymptotic time and running length, and prediction of performances at future editions of the Olympic Games. **a.** Relation between the best estimates of the limiting performance value 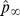 and the length 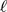 of the race for men running events in athletics (red circles). We excluded from the analysis relay and hurdles events. We find that 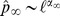, and the best estimate of the power-law exponent is 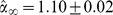 (black line). **b.** Probability density functions of the winning time for the men 400 meters sprint in future editions of the Games. The dashed line represents the winning time in the latest edition of the Olympics in Beijing 2008. This value is used as initial condition for the prediction of future performances. **c.** The probability density of the winning time in men 400 meters predicted by our model is compared to past performance data (black circles). The density plot is obtained by convoluting the various prediction curves derived from real data. **d.** Probability that athletes will breach challenging walls in various specialties of athletics as a function of time.

The good accuracy of our best estimates of the limiting performance values is supported also by the power-law relation between these quantities and the length of the running events in athletics (see Fig. 3a). As already observed by Katz and Katz, world record times (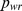) and running distances (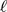) are related by the power-law relation 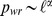 [21]. Katz and Katz studied the relation between world record performances and running distances in various epochs, and found that the power-law exponent value 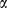 is always slightly larger than 1.1 but decreases for more recent epochs. For example, they measured 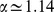 in 1925, and 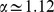 in 1995. On the basis of our measurements, we claim that the asymptotic value of the exponent will be exactly 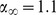, when limiting performance values, and thus definitive world records, will be reached in all specialties of athletics.

A final application of our findings is the prediction of future performances at the Olympics. The performance value of the gold medalist in London 2012, for example, can be estimated as 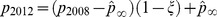, where 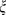 is a random variate extracted from the normal distribution 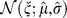 with mean value 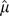 and standard deviation 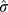. Similar equations can be written also to predict performance values of the other editions after London 2012. For each future edition of the Games, we can draw a distribution of performance values (see Fig. 3b). The distribution is normal for the edition of 2012, but diverges from normality as time grows. In particular, while the expected performance value decreases exponentially towards the asymptotic performance value as time increases, the standard deviation initially grows as we move further in future until predictions become again more accurate because of the boundary effect of 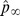 (see Fig. 3c).

By simply looking at the performances expected at the next edition of the Games in London 2012, we can ask what is the probability that the winner of the gold-medal will beat the actual world record of her/his specialty. In Table 1, we list these probabilities for some specialties together with the most likely performance values that gold-medal winners will obtain. In athletics, there are not negligible chances (about 30%) that the actual world records of 100 meters, 110 meters hurdles and marathon will be lowered by men. In swimming specialties, the expectations are more promising: there is a good probability (higher than 70%) that the world record of 1,500 meters freestyle will be beaten by male athletes.

Relevant limits are unlikely to be broken at the next Olympics (Fig. 3d). We will have to wait until 2020 in order to have a 50% chance that a man will run the 100 meters in less than 9.50 seconds. For other specialties, expectations (probability higher than 50%) are even less promising: men will run the 400 meters in less than 43.00 seconds and the marathon in less than two hours (7,200 seconds) only after 2030, women will run the 100 meters sprint in less than 10.40 seconds only after 2040, and finally the wall of 26 minutes (1,560 seconds) in 10,000 meters will likely be breached by male athletes only after year 2080.

## Discussion

In conclusion, our paper shows that the performance of Olympic medal winners in athletics and swimming obey, independently of the type of specialty, a simple universal law. If performance improvements are calculated with respect to an asymptotic performance value, then the relative difference between improvements obtained in two different editions of the Games is a random variate following a normal distribution. This is the common property of a broad class of natural phenomena that be described by the theory of biased random walks [34], such as the locomotory movements of organisms responding to an external stimulus [35]–[37], the activity of spiking neurons [38], the trends of daily temperatures [39], stock prices [40], capital markets [41], etc.

The normality of the relative improvements cannot be explained in trivial terms, especially in this case where the statistics is performed on extremal properties of the system. Remember in fact that the performance values analyzed here are those obtained by the best athletes of a given edition of the Olympics (i.e., potentially the best performers on the earth), and thus it is natural to expect that absolute performance values obey statistical laws of extremes [42]. More importantly, since the distribution is normal, it makes sense to refer to average trajectories of top performance values along editions of the Games. Our findings in fact allow to say that, on average, the absolute performance value of top athletes at the Olympics gets closer to the limiting performance value in an exponential fashion, with a rate of about 5% in athletics and 10% in swimming. More in detail, the average trajectory of the performance value can be described by the equation
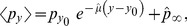
(2)where 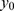 is an arbitrary initial edition year of the Olympics and 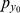 is the performance value measured in year 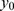. Eq.2 can be derived directly from Eq.1 and the fact that relative improvements are normally distributed but only under the assumptions that the edition year of the Olympics is considered as a continuous variable and that 
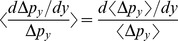
. Note that this observation is important for stressing the difference between our fitting procedure and a more straightforward analysis based on the exponential fit of absolute performance values, as the one used to find that the progression of world record performances follows a piecewise exponential decaying pattern [43]–[45]. Note also that the analysis of the only Olympic performances differs from the one of world record performances for the following reasons: (i) The relative change between two world records, if defined in a similar manner as Eq.1, can be only a positive quantity; (ii) The time difference between two world record performances is not a constant, but a random variate by itself. Because the number of events in which new world records can be established is higher today than it was one century ago (and they had been growing in the course of the years), in any analysis of the progression of world record performances time should be rescaled to account for that [43].

The asymptotic performance value 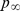 is an *a priori* unknown variable whose value can be self-consistently determined by maximizing the statistical significance of the normality fit. It is particularly important to stress that our simple methodology provides good estimates of performance limits that are in general consistent with those obtained through complicated physiological models [31]–[33]. For example, Perronet and Thibault predicted that the limiting time for men in marathon is 1 hour, 48 minutes and 26 seconds [32]. With our minimalistic model, we are able to predict that this limiting time is between 1 hour, 36 minutes and 11 seconds and 1 hour, 41 minutes and 40 seconds (for men marathon the peak of statistical significance is wide, see Fig. 2a). At the same time, it is also important to stress that our minimalistic analysis can also lead to little inconsistencies. For example, the best estimates of 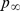 obtained here state that, asymptotically, the average pace in marathon would be higher than the one in 10,000 meters. This means that according to our estimates, the first 10,000 meters in marathon would be run in less than 23 minutes, while the entire race of 10,000 meters would be run asymptotically in more than 25 minutes. This inconsistency can be partially explained by the fact that the statistics for 10,000 meters is less reliable because based only on 22 events, while the one for marathon on the results of 26 editions of the Games. In general, it is very important to remark that, at the moment, we are able to provide only good estimates of the asymptotic performance values because such estimates are based on a relatively small set of empirical data (at best 26 editions of the Olympics), and therefore must be taken with a grain of salt. We expect in fact that, while the normal law governing performance improvements will likely continue to hold, the accuracy in the estimation of the asymptotic performance values will improve with the addition of more data points in the future, starting already from the next edition of the Games in London 2012.

## Materials and Methods

### Data Set

Medal lists and results of all editions of the Olympic Games have been collected from the web sites www.sports-reference.com and www.databaseolympics.com. Whenever possible, we considered automatic measures of time instead of manual ones. We included in our study all results obtained in the editions of the modern Olympic Games since Athens 1896, but we excluded from the analysis data about the so-called “Intercalated” edition of the Games held in Athens in 1906. We focused on sports classified as “Track & Field” and “Swimming”, and particularly on specialties of these sports that have been performed at least in the latest ten editions of the Olympic Games. We compared only performances between subsequent editions of the games held at four years of difference. We excluded therefore comparisons between either the consecutive editions of Stockholm 1912 and Antwerp 1920 (separated by World War I), and those of Berlin 1936 and London 1948 (separated by World War II).

For consistency, we considered only specialties whose rules or techniques have not changed during time. For example, we excluded javelin throw because of the javelin redesign in 1986. We also excluded performances in high jump before Mexico City 1968 when athletes started for the first time to adopt the modern jump style called “Fosbury flop”.

Data are made available for download at filrad.homelinux.org/resources.

### Normality Test

The results reported in the paper are based on the normality test introduced by Anderson and Darling [46]. Given a value of 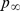, we compute the best estimates of the mean 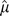 and the standard deviation 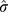 as 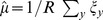 and 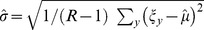, respectively. The relative improvement 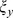 is defined in Eq.1. *R* indicates the number of results between consecutive editions of the Olympic Games that are included in the analysis. We then compute the 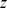-scores as 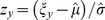 and rearrange them in ascending order such that 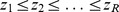. The Anderson-Darling distance is computed with the formula 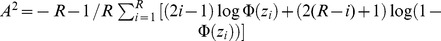, where 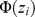 is the standard normal cumulative distribution function. We further use the modified statistics 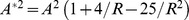, suitable in the case in which both the mean and standard deviation are estimated from the data as suggested by Stephens [47].

We evaluate the goodness of the fit by generating 10^5^ random number sequences of length *R* extracted from the standard normal distribution. The statistical significance of the normality test (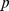-value) is calculated as the number of artificial sequences whose 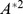 is larger than the one measured for real data divided by the total number of generated sequences. Note that there is a trivial monotonic relation between the 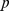-value and the Anderson-Darling distance 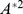, and therefore the maximum of the 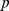-value corresponds to the minimum of 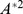.

We used the normality test by Anderson and Darling because this test is considered one of the best empirical distribution function statistics for detecting most departures from normality, and can be used for testing the normality of very small sample sizes [47]. We verified, however, the robustness of our results by using other standard normality tests, including those based on the criteria of Kolmogorov-Smirnov, Cramér-von Mises and Shapiro-Wilk [48], [49]. We also verified the consistency of our results with normality tests based on the moments of the distributions (see Fig. S6).

Furthermore, we tested the accuracy of our fitting method by implementing a bootstrap procedure [50], and found that our fitting method is able to well recover the correct parameter values in artificial sequences generated according to our model (see Fig. S7).

## Supporting Information

Figure S1 **Men 400 meters.**
(PDF)

Figure S2 **Men 400 meters.**
(PDF)

Figure S3 **Comparison of male and female performances in 100 meters sprint between 1928 and 2008.**
(PDF)

Figure S4 **Men 100 meters.**
(PDF)

Figure S5 **Women 100 meters.**
(PDF)

Figure S6 **Analysis based on the excess kurtosis.**
(PDF)

Figure S7 **Sensitivity test for the fitting procedure.**
(PDF)

Supporting Information S1 Complete analysis of “track” specialties in athletics.
(PDF)

Supporting Information S2 Complete analysis of “field” specialties in athletics.
(PDF)

Supporting Information S3 Complete analysis of swimming specialties.
(PDF)
