# Supplementary material for: Universality, Limits and Predictability of Gold-Medal Performances at the Olympic Games
Source: PLoS One. 2012 Jul 12;7(7):e40335. doi: 10.1371/journal.pone.0040335 (PMC3395717; doi:10.1371/journal.pone.0040335)
Supplement: Figure S1 — Men 400 meters. (PDF) [file pone.0040335.s001.pdf]

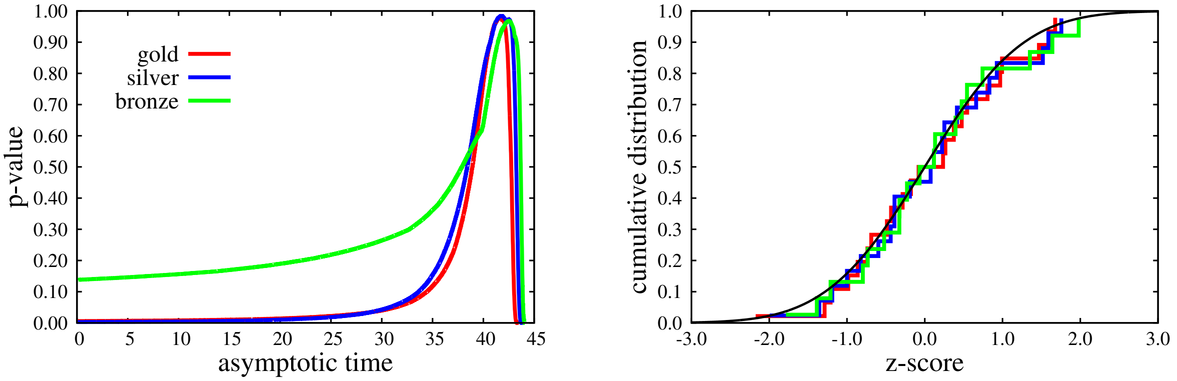

Figure S1: Men 400 meters. Separate analysis for performance data of gold, silver and bronze medalists.
