# Supplementary material for: Universality, Limits and Predictability of Gold-Medal Performances at the Olympic Games
Source: PLoS One. 2012 Jul 12;7(7):e40335. doi: 10.1371/journal.pone.0040335 (PMC3395717; doi:10.1371/journal.pone.0040335)
Supplement: Figure S2 — Men 400 meters. (PDF) [file pone.0040335.s002.pdf]

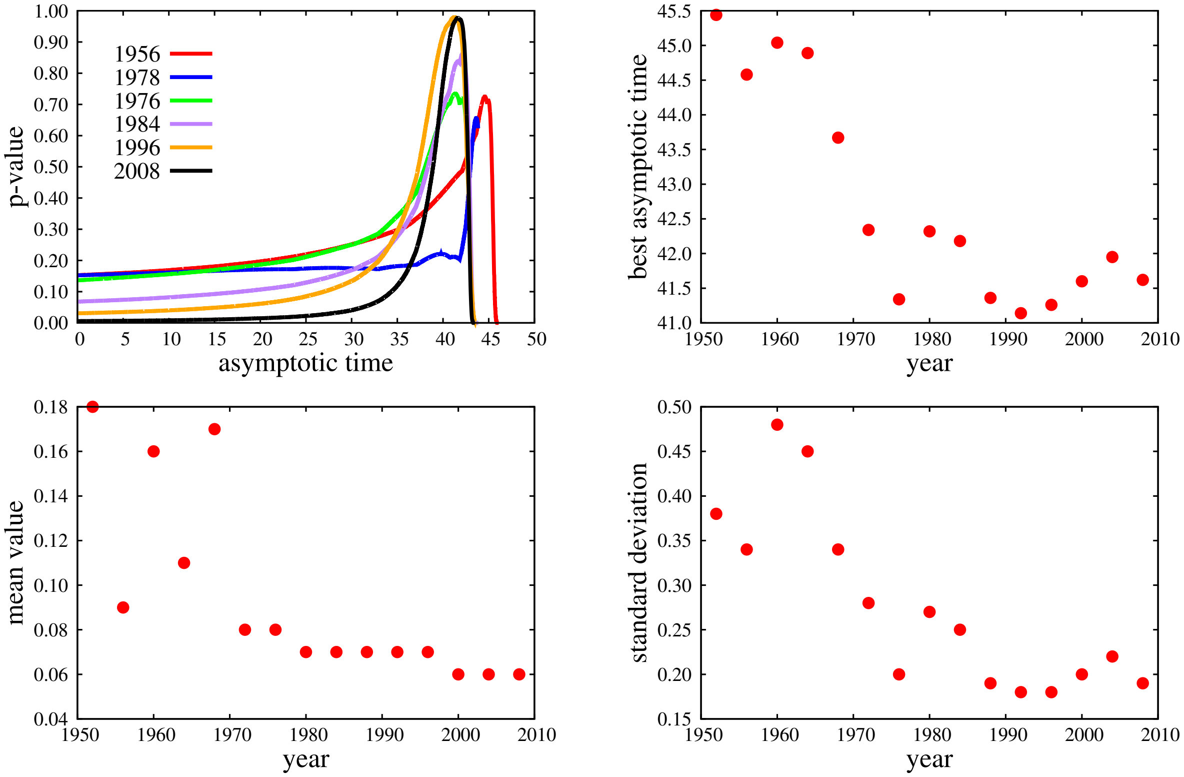

Figure S2: Men 400 meters. We apply our method to shorter time series. We consider all results between 1896 and a given ending year. In the upper left panel, we show the determination of the best estimate of the asymptotic time for various ending years. In the other panels, we plot the best estimates of the asymptotic time, mean value and standard deviation as functions of the ending year.
