# Supplementary material for: Universality, Limits and Predictability of Gold-Medal Performances at the Olympic Games
Source: PLoS One. 2012 Jul 12;7(7):e40335. doi: 10.1371/journal.pone.0040335 (PMC3395717; doi:10.1371/journal.pone.0040335)
Supplement: Figure S3 — Comparison of male and female performances in 100 meters sprint between 1928 and 2008. (PDF) [file pone.0040335.s003.pdf]

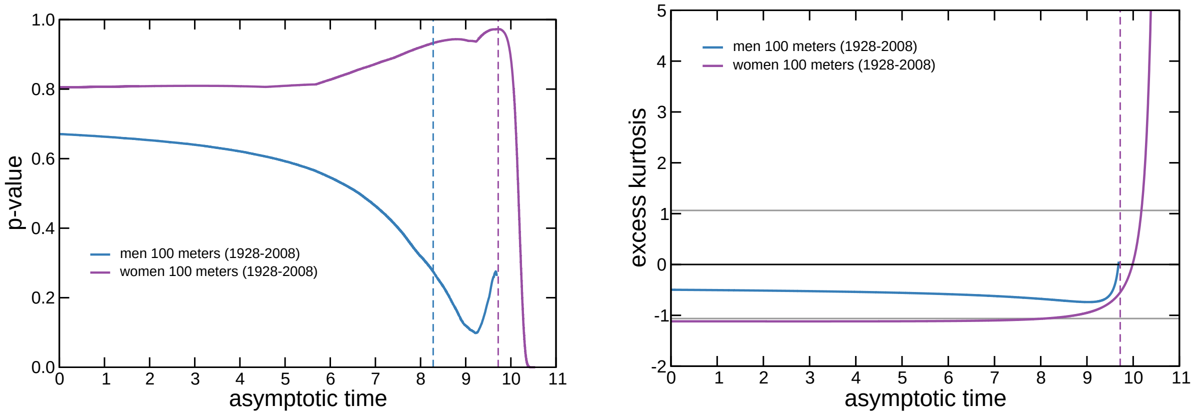

Figure S3: Comparison of male and female performances in 100 meters sprint between 1928 and 2008. Left. Statistical significance (p-value) as a function of p_∞ for performance data of males (blue line) and females (purple line) in 100 meters sprint olympic events held between 1928 and 2008. The dashed blue line corresponds to the best estimate of p_∞ computed on the entire time window 1896-2008. Right. Excess kurtosis as a function of p_∞ for performance data of males (blue line) and females (purple line) in 100 meters sprint olympic events held between 1928 and 2008. The gray lines bound the region of one standard deviation away from zero. The dashed purple line corresponds to the best estimate of p_∞ corresponding to the maximum of statistical significance.
