# Supplementary material for: Universality, Limits and Predictability of Gold-Medal Performances at the Olympic Games
Source: PLoS One. 2012 Jul 12;7(7):e40335. doi: 10.1371/journal.pone.0040335 (PMC3395717; doi:10.1371/journal.pone.0040335)
Supplement: Figure S5 — Women 100 meters. (PDF) [file pone.0040335.s005.pdf]

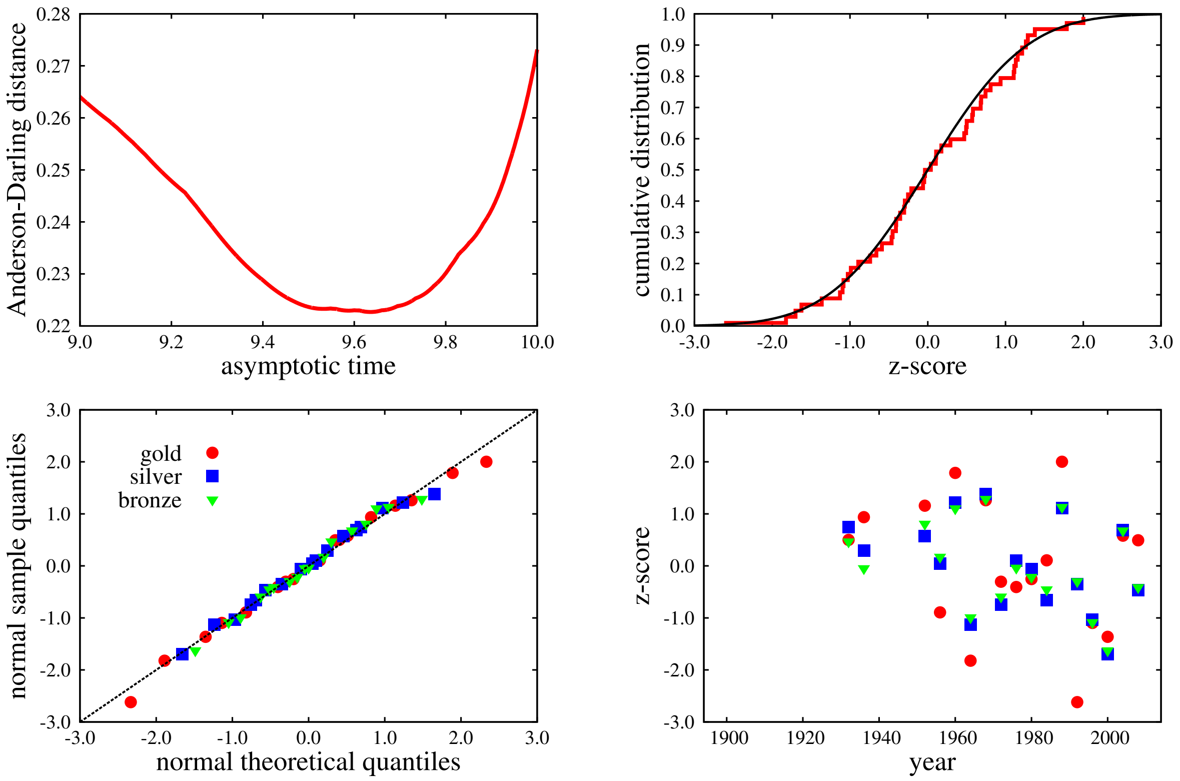

Figure S5: Women 100 meters. We included in the analysis the results of gold, silver and bronze medalists. In this case, we could not provide an estimation of the statistical significance due to the intrinsic correlations among variables (the improvement in year y of gold medalists is positively correlated with those of the same year obtained by silver and gold medalists). The best estimate of p_∞ is instead identified as the value for which the Anderson-Darling distance is minimal. We find: $\hat{p}_\infty = 9.64$, $\hat{\mu} = 0.05$, $\hat{\sigma} = 0.14$ and $A^{*2} = 0.22$.
