# Supplementary material for: Universality, Limits and Predictability of Gold-Medal Performances at the Olympic Games
Source: PLoS One. 2012 Jul 12;7(7):e40335. doi: 10.1371/journal.pone.0040335 (PMC3395717; doi:10.1371/journal.pone.0040335)
Supplement: Figure S6 — Analysis based on the excess kurtosis. (PDF) [file pone.0040335.s006.pdf]

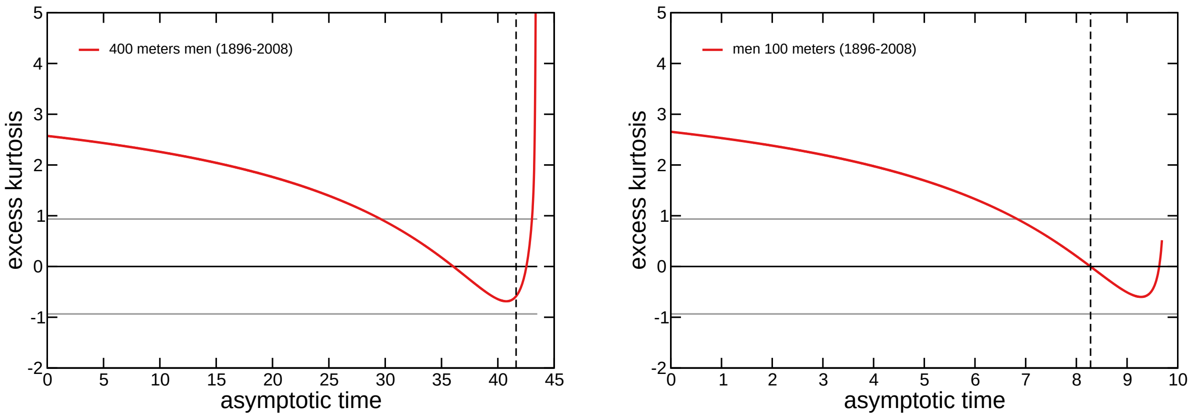

Figure S6: Analysis based on the excess kurtosis. Excess kurtosis ($g_2 = \frac{m_4}{m_2^2} - 3$, where $m_4 = \frac{1}{R}\sum_{i=1}^{R} z_i^4$, $m_2 = \frac{1}{R}\sum_{i=1}^{R} z_i^2$, z_i are the z-scores used also in the other normality tests, and R indicate the number of points included in the analysis) calculated as a function of the asymptotic time (p_∞) for 400 (left) and 100 (right) meters sprint of male athletes. Values of g_2 close to zero generally indicate that the variables for which the excess kurtosis is calculated are normally distributed. Since g_2 is calculated on a finite number of points the gray lines bound the region corresponding to one standard deviation away from the expected value of the excess kurtosis for a normal distribution ($\langle g_2 \rangle = 0$, black line). The variance of the excess kurtosis for a finite sample of size R is $\sigma_{g_2}^2 = \frac{4\left[6R(R-1)^2(R+1)\right]}{(R-3)(R-2)(R+1)(R+3)(R+5)}$. Finally as terms of comparison, the dashed lines indicate the best estimates $\hat{p}_\infty$ obtained with the Anderson-Darlington test.
