# Supplementary material for: Universality, Limits and Predictability of Gold-Medal Performances at the Olympic Games
Source: PLoS One. 2012 Jul 12;7(7):e40335. doi: 10.1371/journal.pone.0040335 (PMC3395717; doi:10.1371/journal.pone.0040335)
Supplement: Figure S7 — Sensitivity test for the fitting procedure. (PDF) [file pone.0040335.s007.pdf]

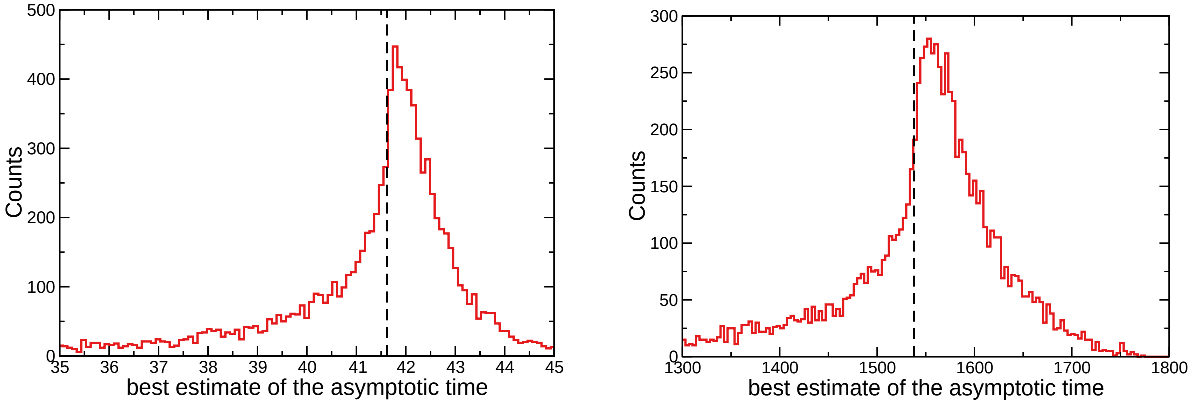

Figure S7: Sensitivity test for the fitting procedure. We fix the initial performance value equal to the one of Athens 1896 and generate artificial time series of performance values according to our model with μ, σ and p_∞ equal to the best estimates obtained on real data. We then evaluate the best estimate of p_∞ with our fitting procedure on the artificial sequence. We repeat the operation on $10,000$ artificial series and we report here the histograms of the evaluated p_∞ (red). The dashed black line represent the value of $\hat{p}_\infty$ measured on real data. We present two examples: 400 meters sprint and 10,000 meters, both run by male athletes.
