# Supplementary material for: Universality, Limits and Predictability of Gold-Medal Performances at the Olympic Games
Source: PLoS One. 2012 Jul 12;7(7):e40335. doi: 10.1371/journal.pone.0040335 (PMC3395717; doi:10.1371/journal.pone.0040335)
Supplement: Supporting Information S1 — Complete analysis of “track” specialties in athletics. (PDF) [file pone.0040335.s008.pdf]

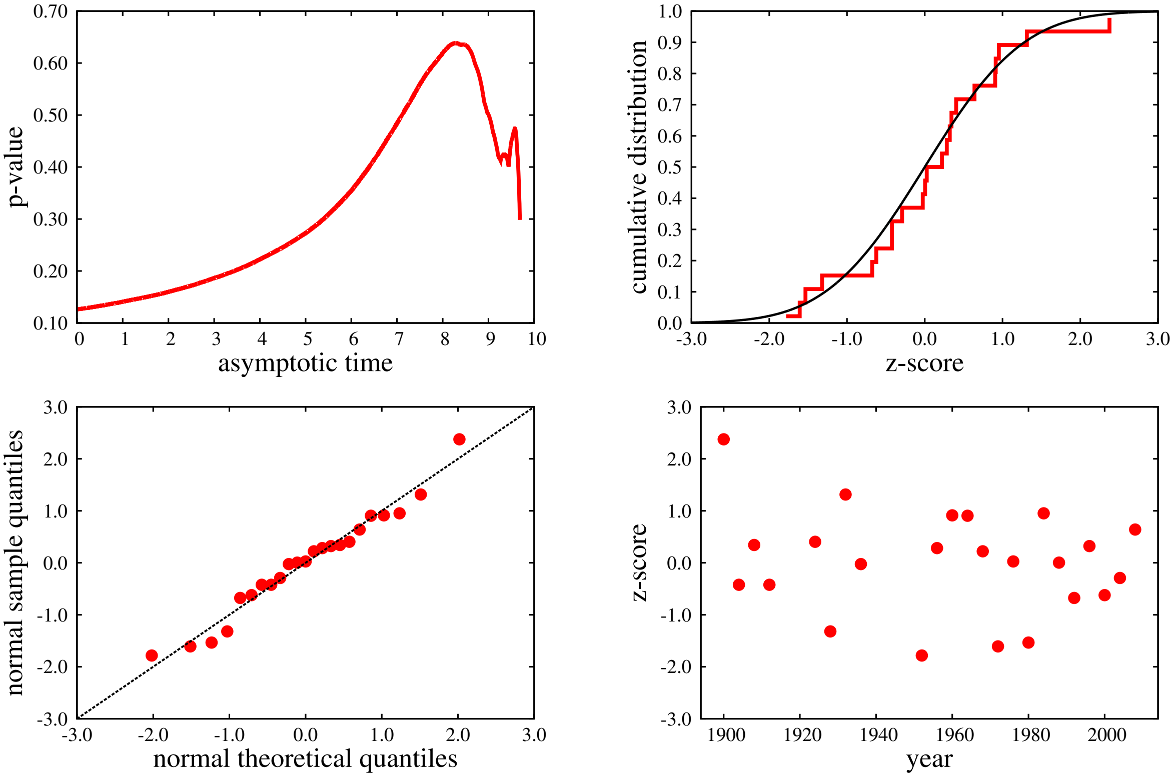

Figure S1.1: Track & Field: men 100 meters

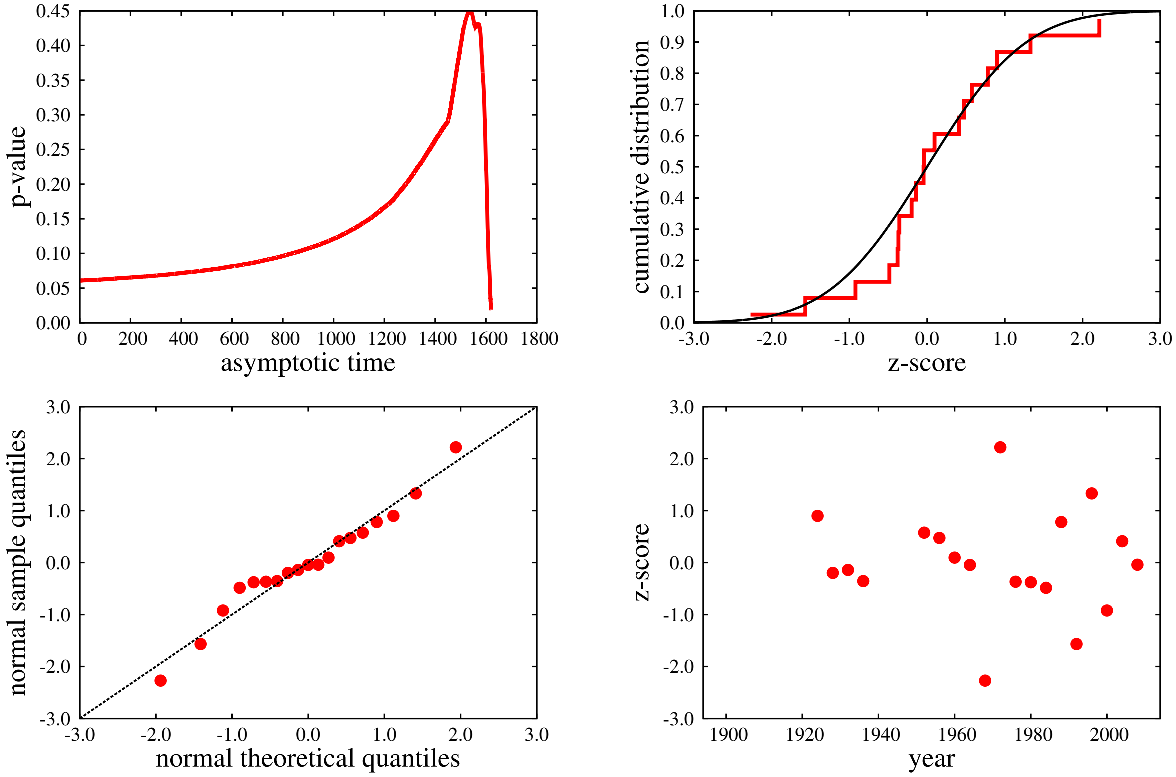

Figure S1.2: Track & Field: men 10000 meters

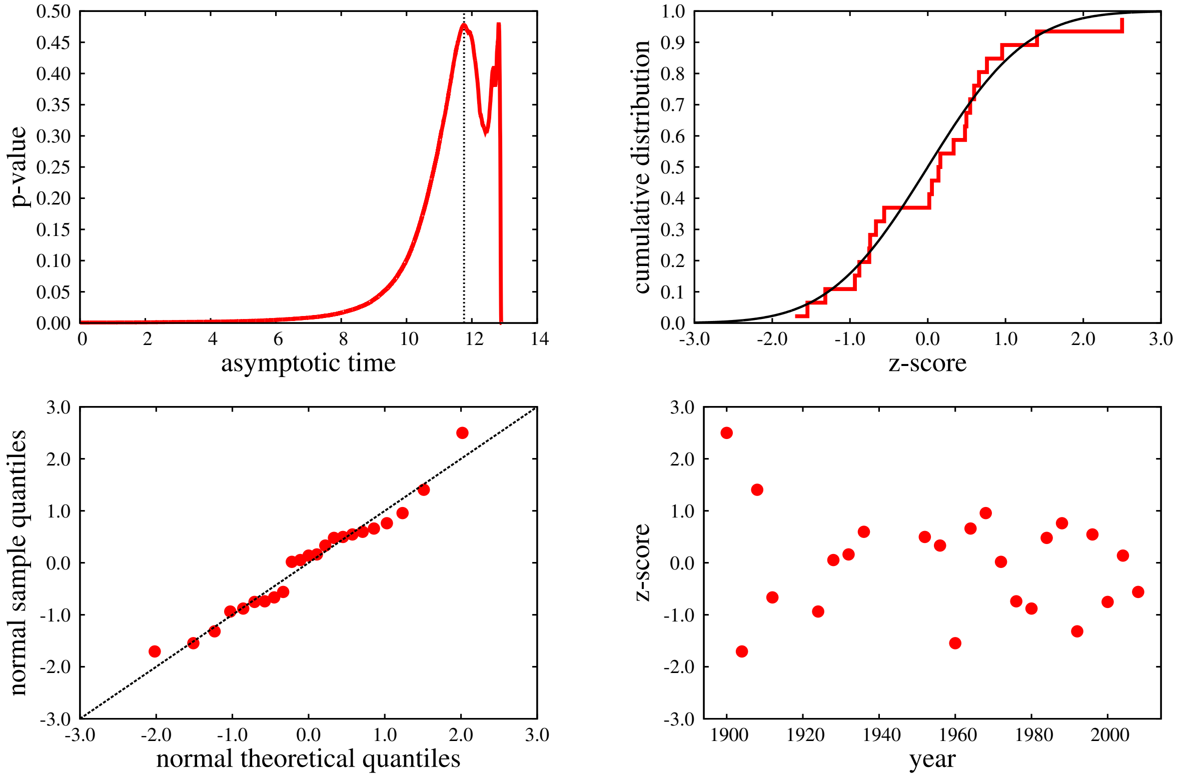

Figure S1.3: Track & Field: men 110 meters hurdles

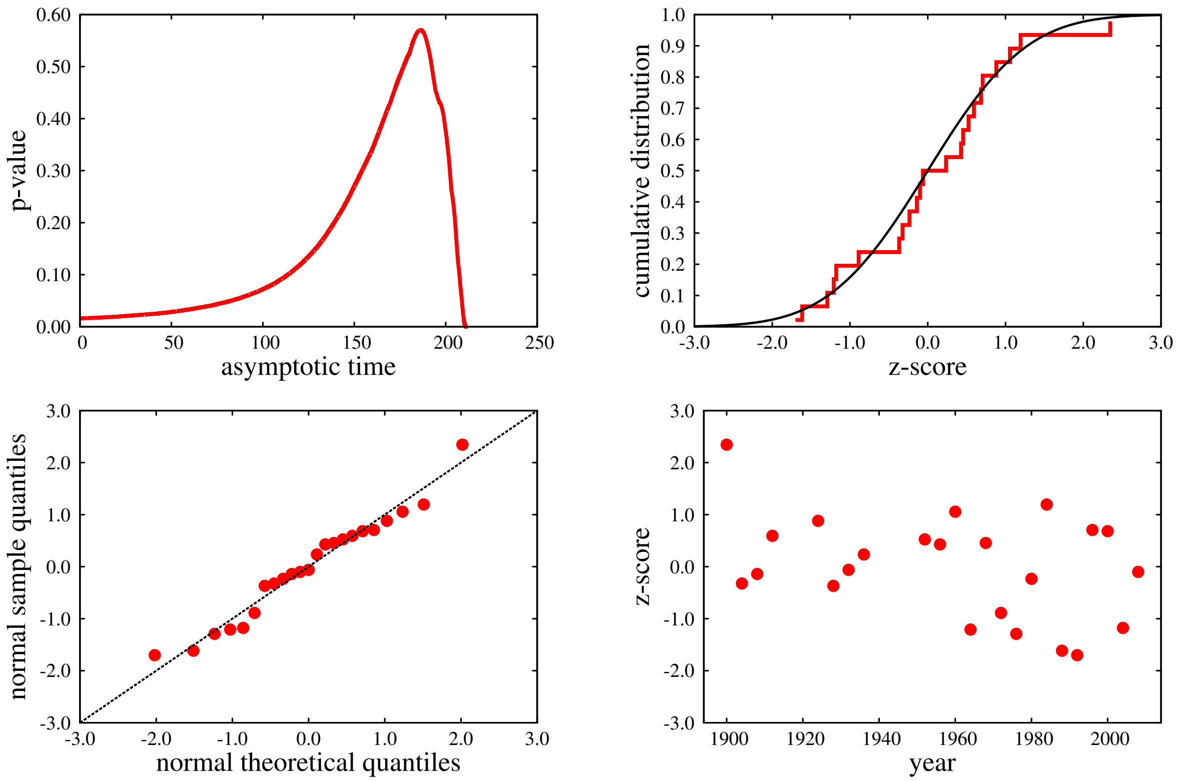

Figure S1.4: Track & Field: men 1500 meters

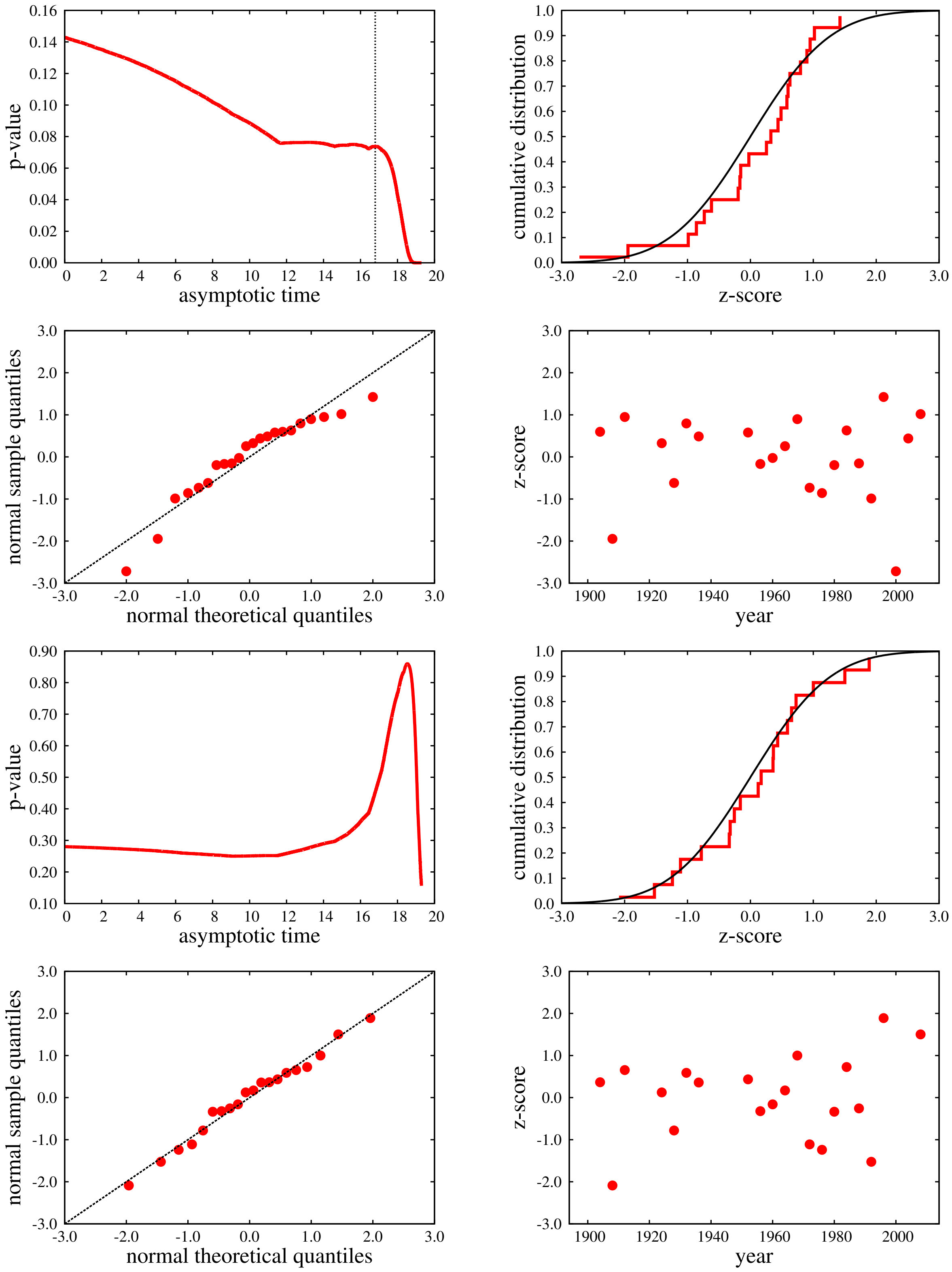

Figure S1.5: Track & Field: men 200 meters. The lower four panels are the results obtained with the exclusion of the performance data of Sidney 2000 ($\hat{p}_\infty = 18.54$).

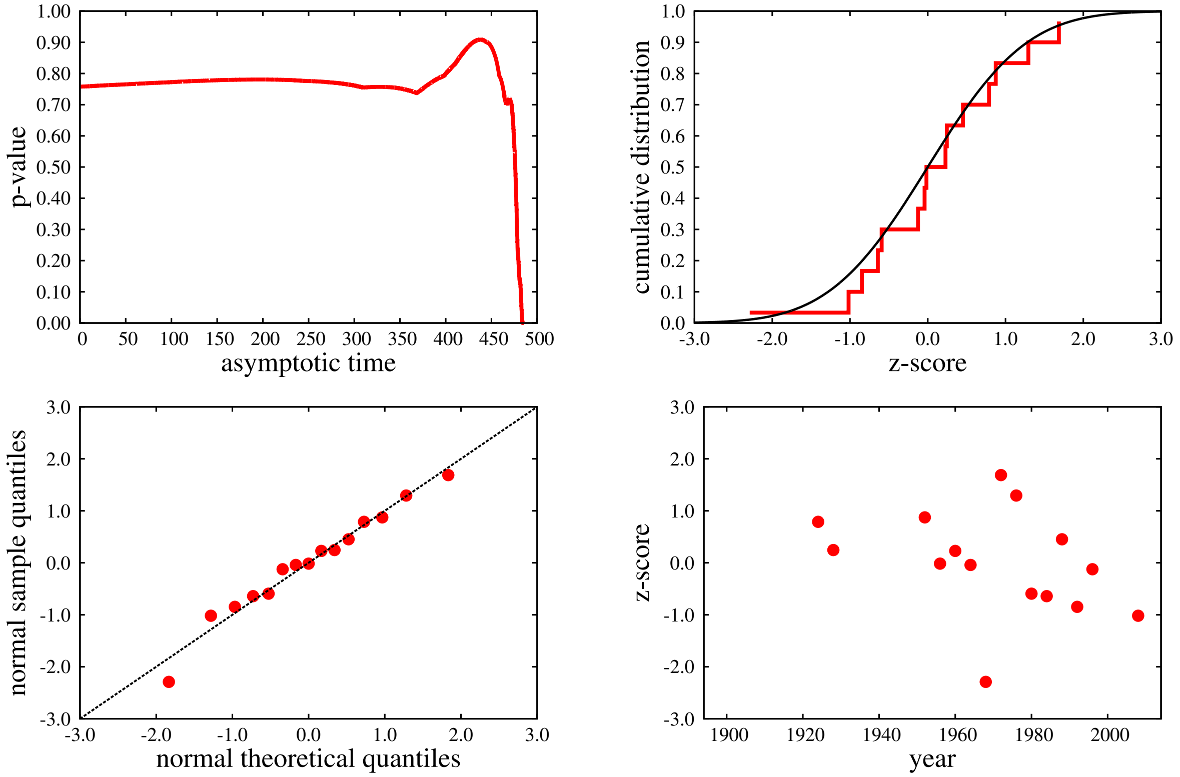

Figure S1.6: Track & Field: men 3000 meters steeplechase

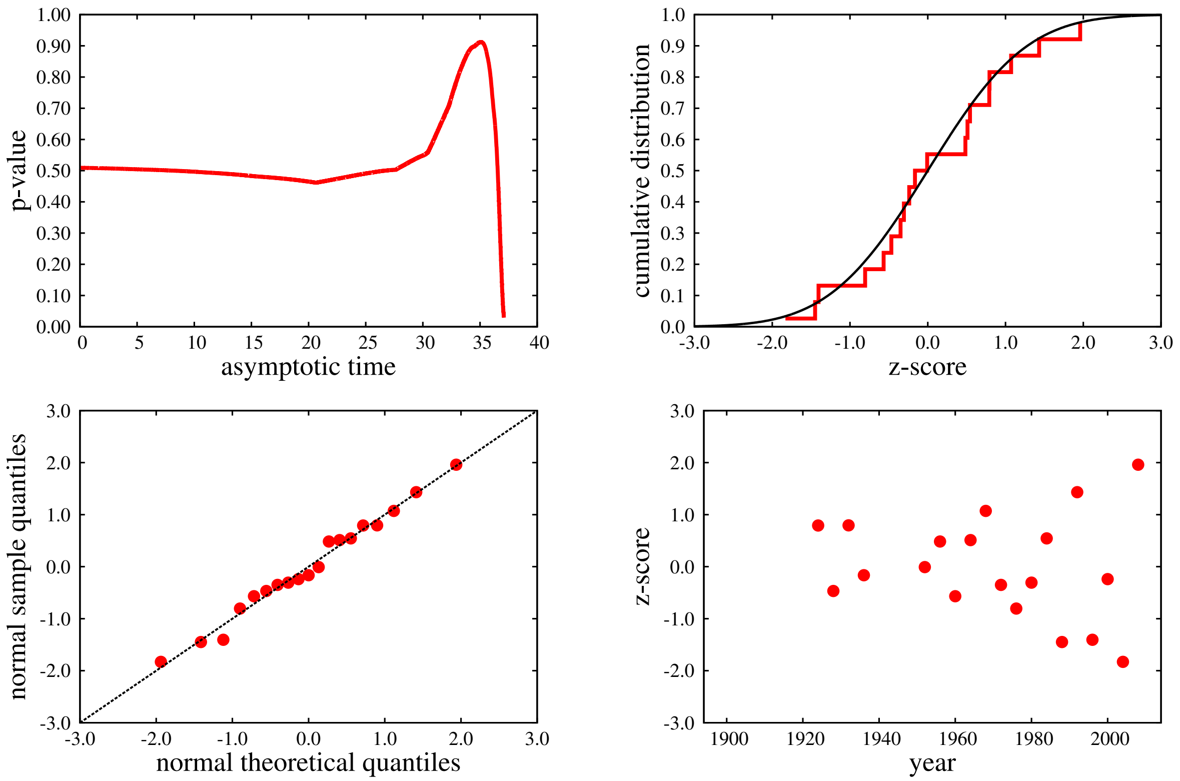

Figure S1.7: Track & Field: men 4 x 100 meters relay

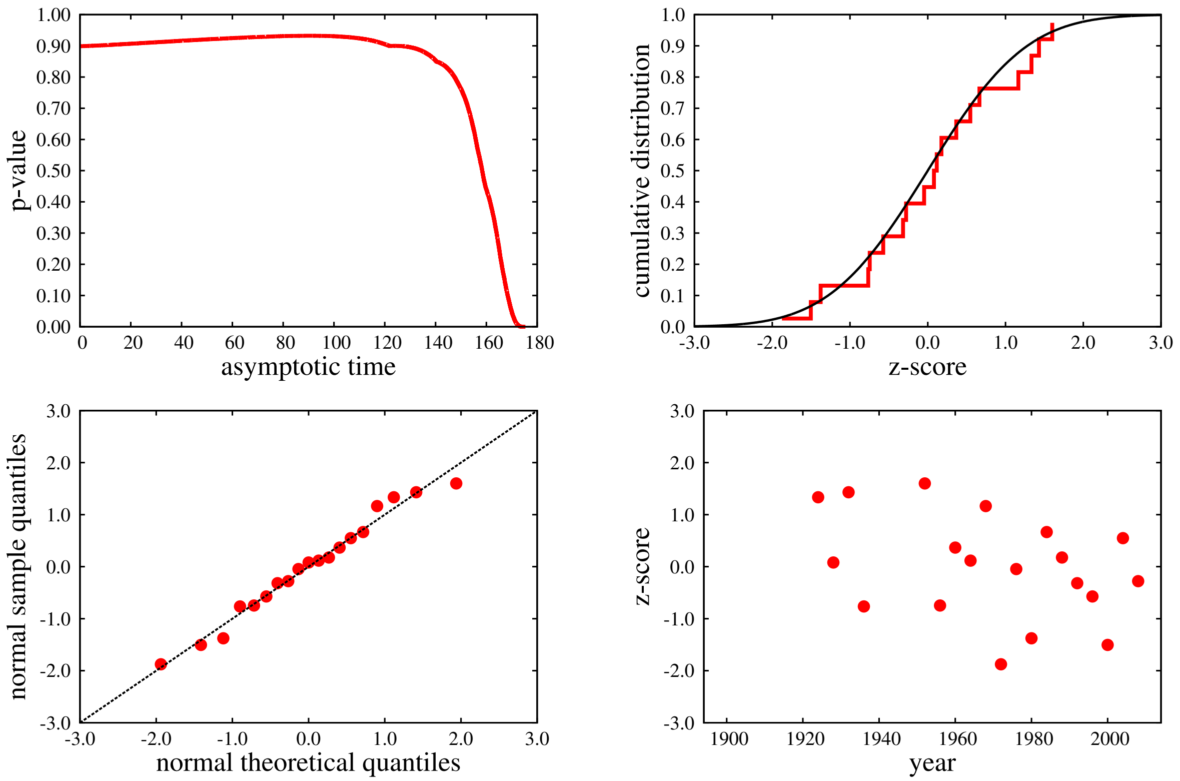

Figure S1.8: Track & Field: men 4 x 400 meters relay

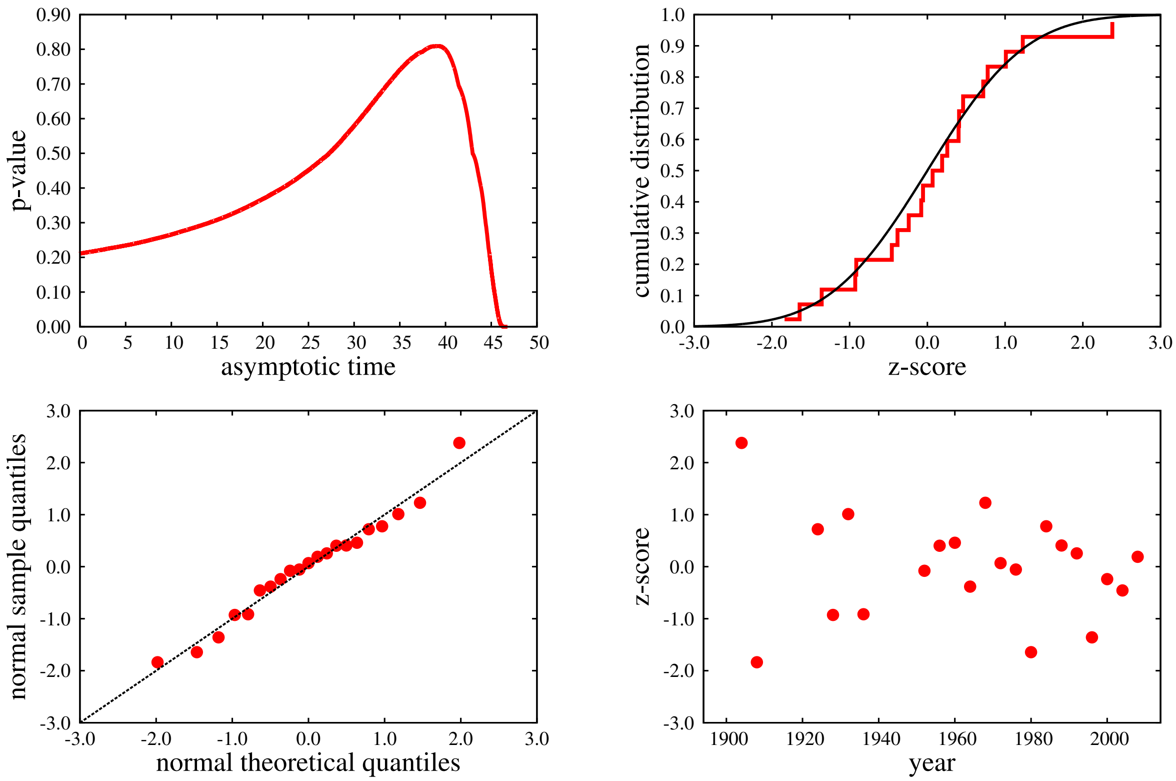

Figure S1.9: Track & Field: men 400 meters hurdles

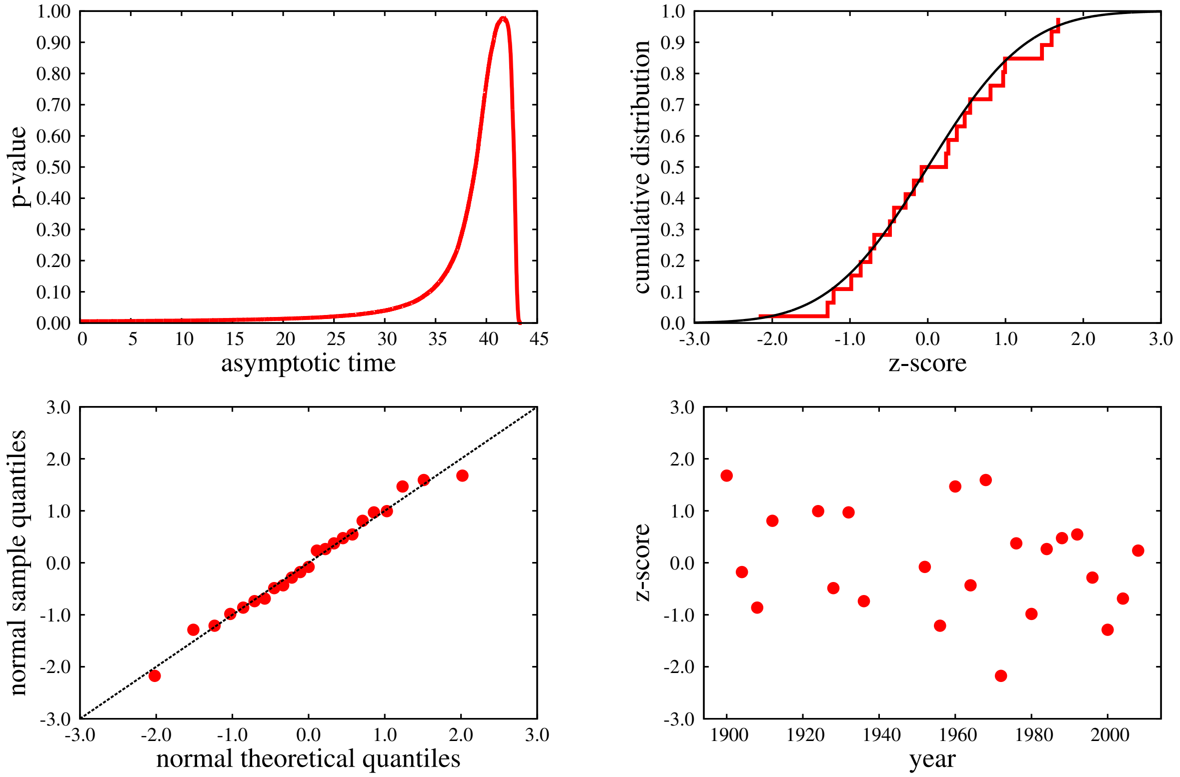

Figure S1.10: Track & Field: men 400 meters

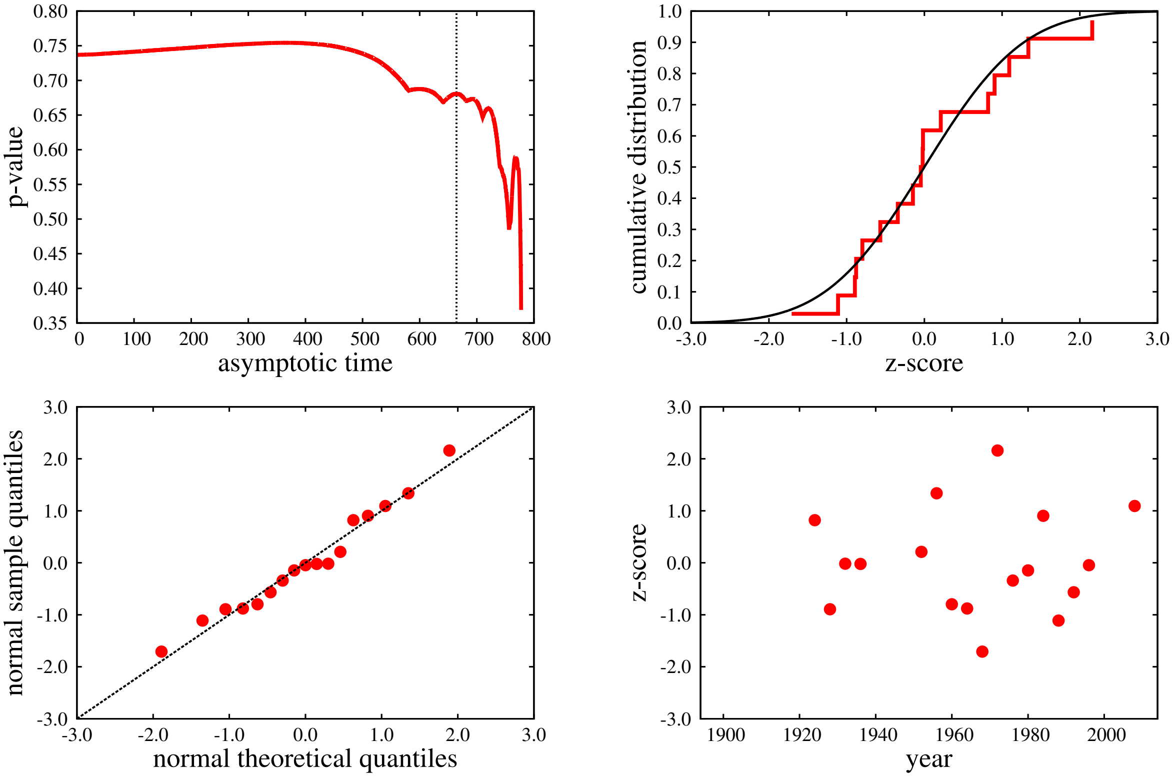

Figure S1.11: Track & Field: men 5000 meters

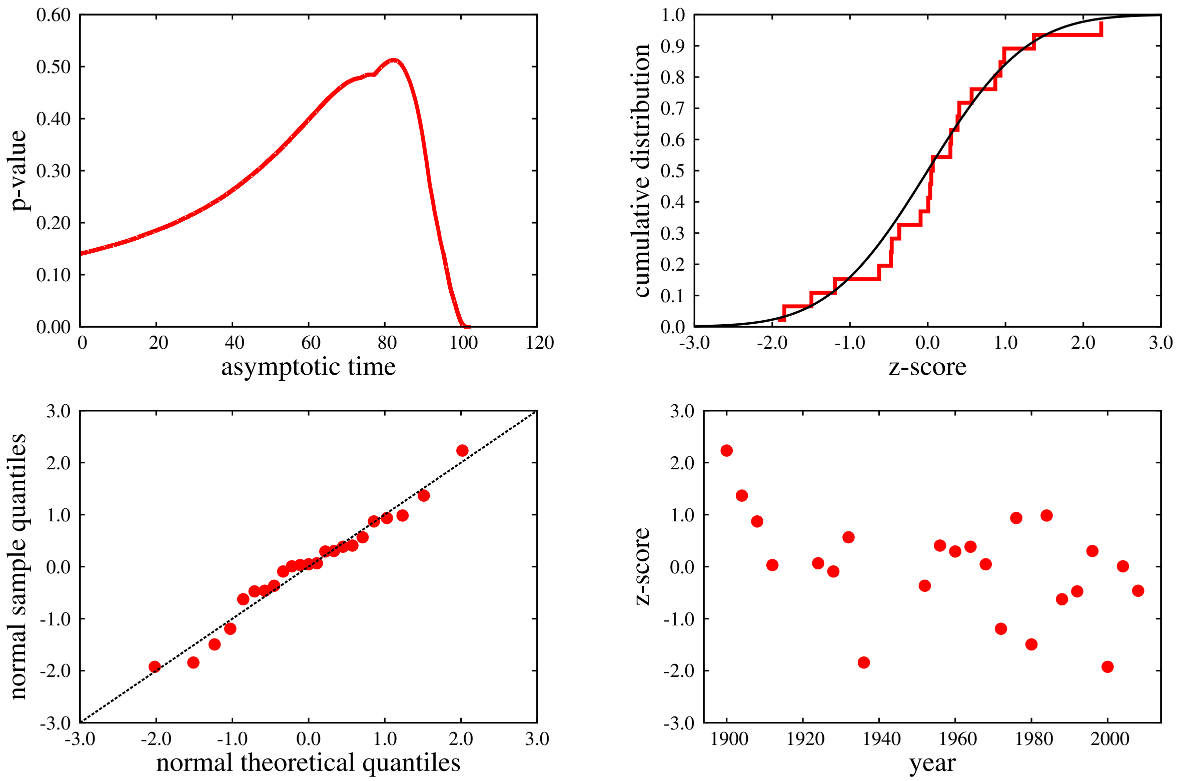

Figure S1.12: Track & Field: men 800 meters

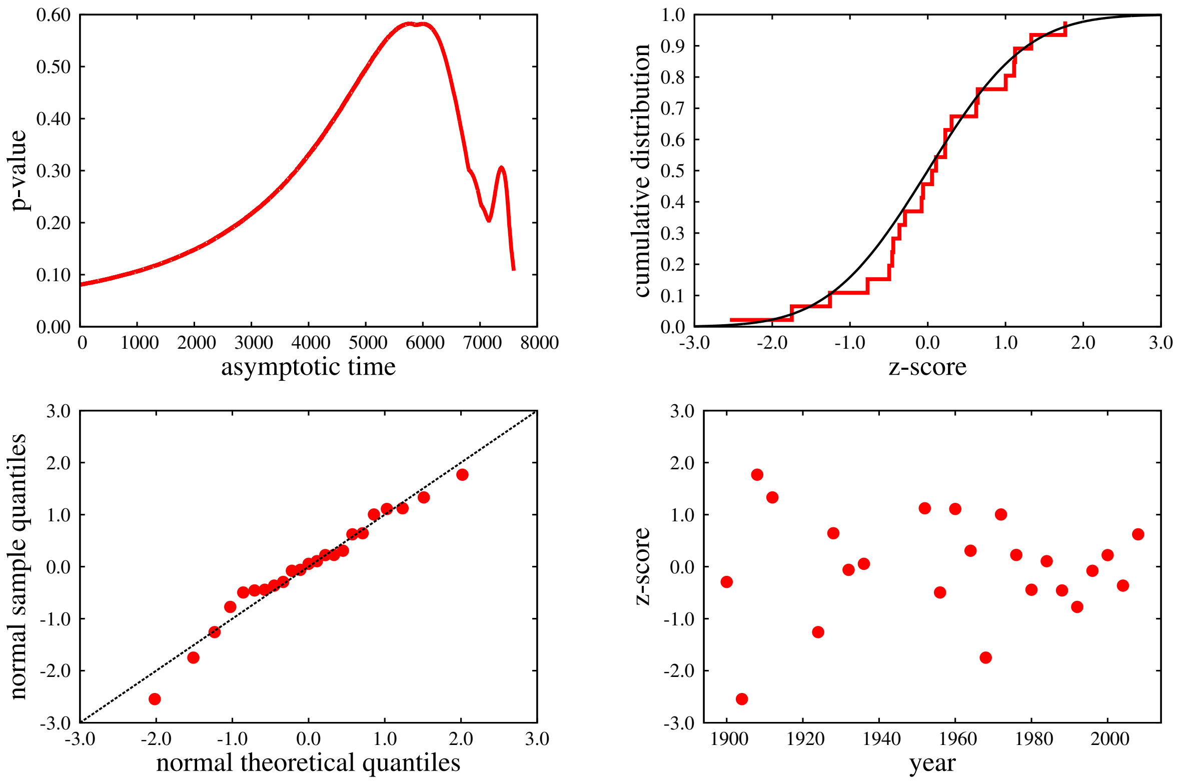

Figure S1.13: Track & Field: men marathon

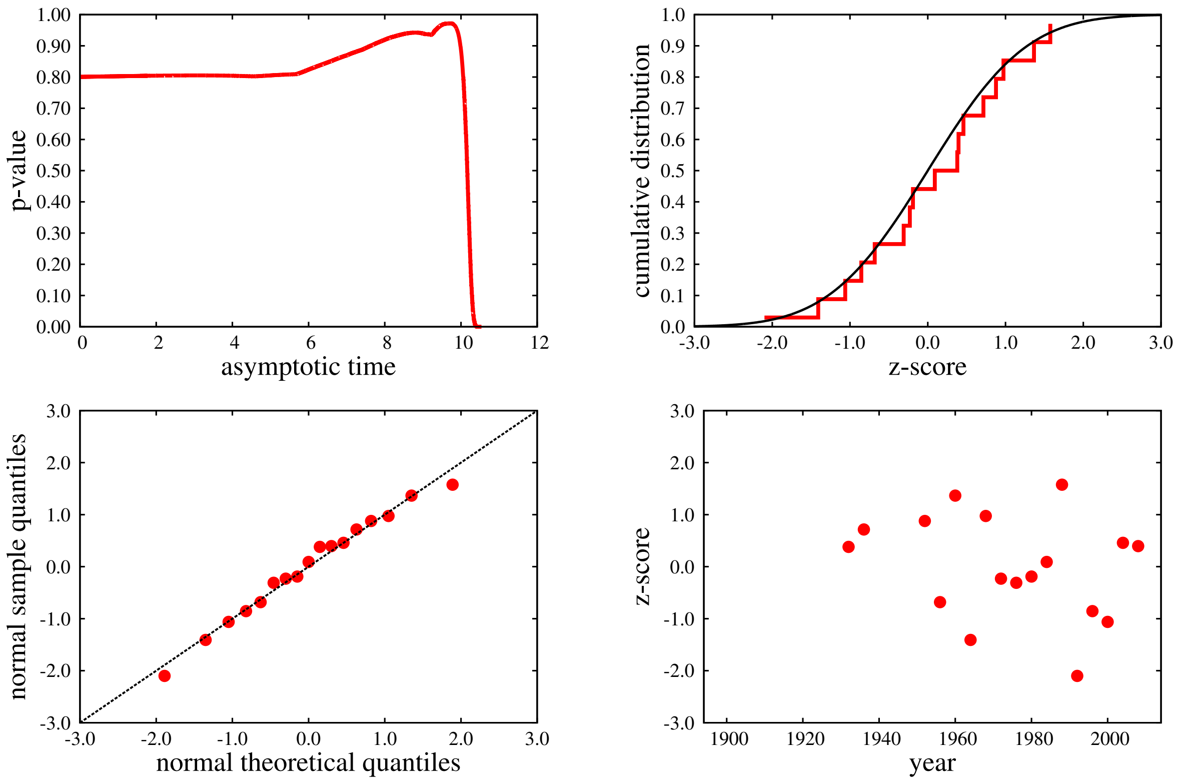

Figure S1.14: Track & Field: women 100 meters

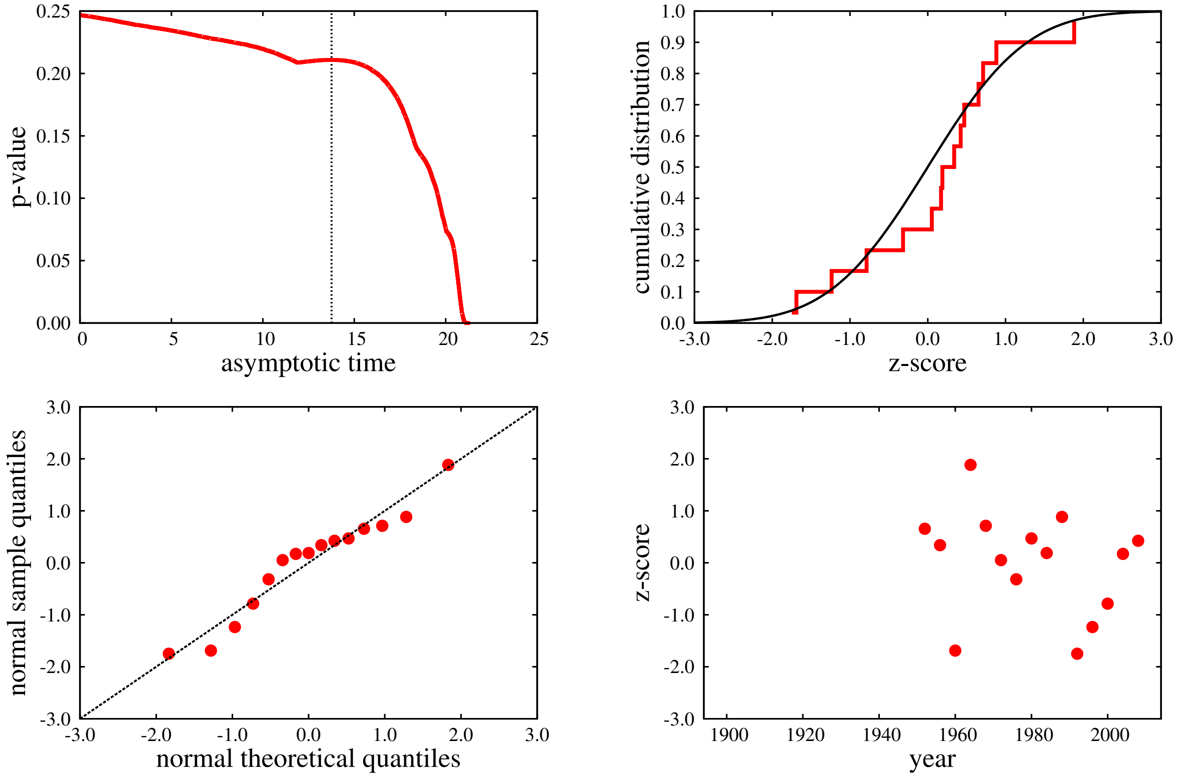

Figure S1.15: Track & Field: women 200 meters

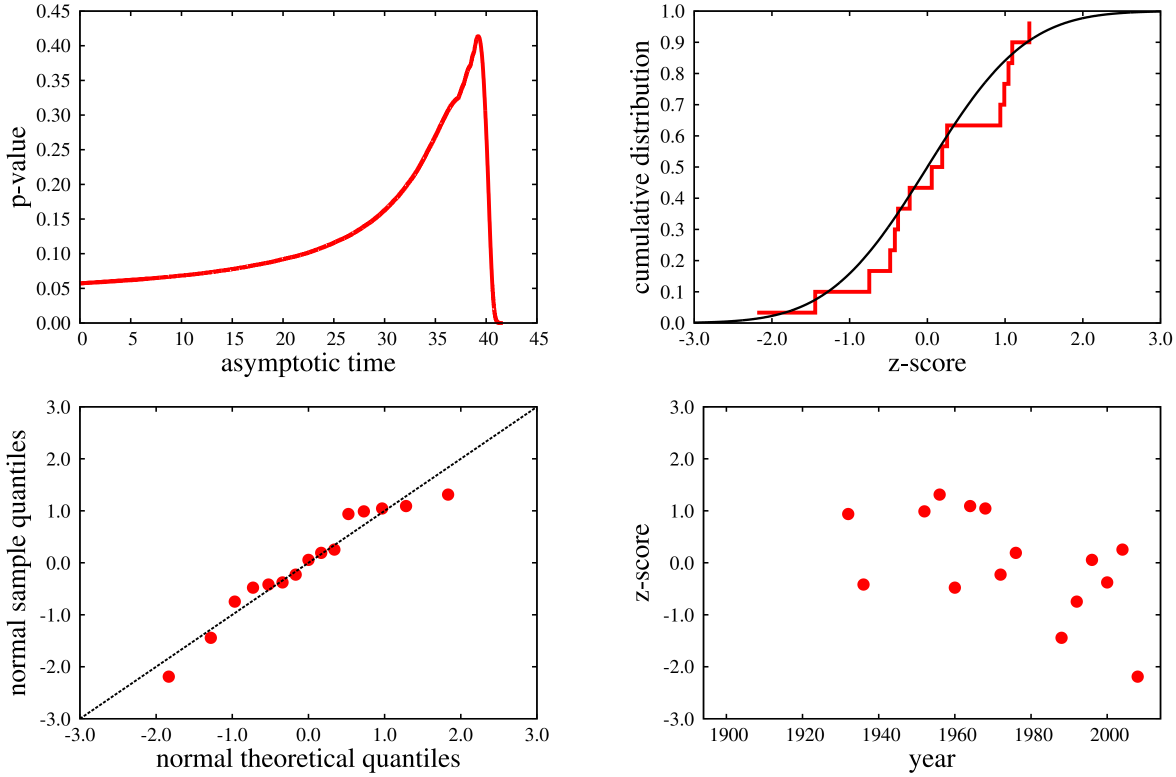

Figure S1.16: Track & Field: women 4 x 100 meters relay

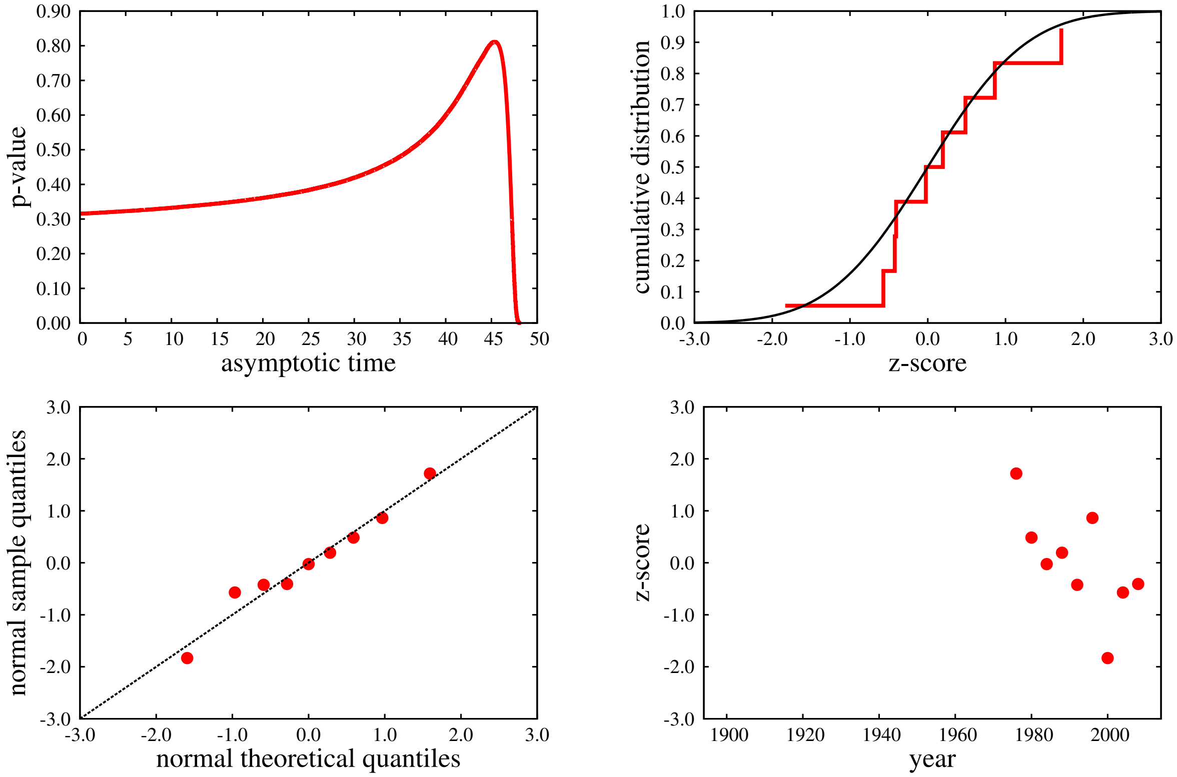

Figure S1.17: Track & Field: women 400 meters

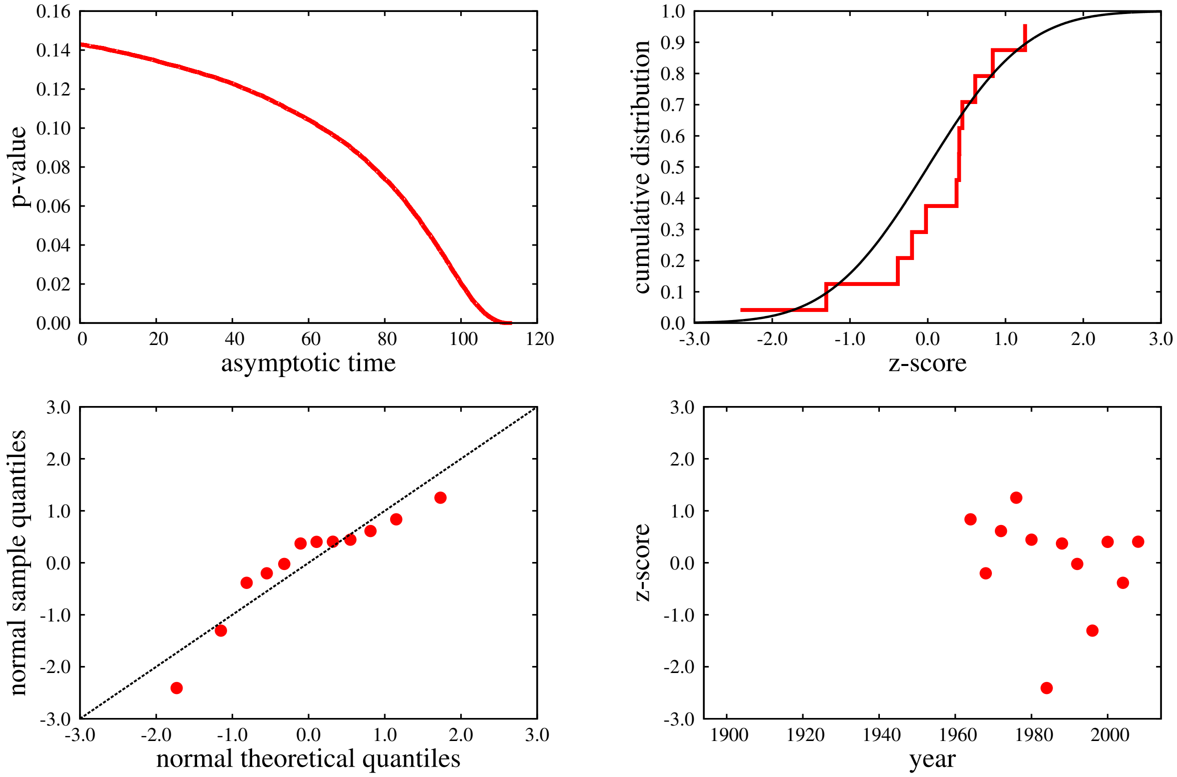

Figure S1.18: Track & Field: women 800 meters

specialty	$\hat{p}_\infty$	$\hat{\mu}$	$\hat{\sigma}$	A^{*2}	p-value	$\hat{p}_\infty^{(u)}$	nr. events
men 100 meters	8.28	0.04	0.10	0.32	0.64	9.69	26
men 10000 meters	1,538.81	0.05	0.19	0.40	0.45	1,616.31	22
men 110 meters hurdles*	11.76	0.05	0.12	0.38	0.48	12.89	26
men 1500 meters	186.20	0.05	0.11	0.34	0.57	208.80	26
men 200 meters*	16.79	0.04	0.12	0.74	0.07	17.91	25
men 3000 meters steeplechase†	437.93	0.05	0.14	0.21	0.91	482.79	19
men 4 x 100 meters relay	35.09	0.06	0.13	0.21	0.91	37.04	22
men 4 x 400 meters relay	90.43	0.01	0.03	0.20	0.93	169.95	22
men 400 meters hurdles	39.17	0.03	0.09	0.25	0.81	45.65	24
men 400 meters	41.62	0.06	0.19	0.16	0.98	43.08	26
men 5000 meters†,*	664.41	0.04	0.08	0.30	0.68	777.66	21
men 800 meters	82.29	0.03	0.08	0.37	0.51	98.09	26
men marathon	5,771.44	0.03	0.15	0.34	0.58	7,590.48	26
women 100 meters	9.72	0.05	0.19	0.17	0.97	10.31	19
women 200 meters*	13.76	0.02	0.05	0.55	0.21	20.55	16
women 4 x 100 meters relay	39.22	0.05	0.13	0.41	0.41	40.63	18
women 400 meters	45.36	0.02	0.17	0.25	0.80	47.65	12
women 800 meters	-	0.02	0.08	0.61	0.05	90.13	13

Table S1.1: Summary table for running specialties in Track & Field. From left to right, we report: the name of the specialty, the best estimate of the asymptotic performance value $\hat{p}_\infty$, the best estimate of the average value $\hat{\mu}$ and standard deviation $\hat{\sigma}$ of performance improvements, the Anderson-Darling distance A^{*2} between sample and theoretical normal distributions, the statistical significance (p-value) of the normal fit, the upper value at 5% significance level of the limiting performance value $\hat{p}_\infty^{(u)}$, and the number of Olympic games that included the specialty. Unless specified, the values of $\hat{\mu}$, $\hat{\sigma}$, A^{*2} and p-value have been calculated at $p_\infty = \hat{p}_\infty$. Results annotated with * have been obtained by identifying the best estimate of the asymptotic time as a local maximum of the p-value. We highlighted in gray the specialties for which we are unable to determine the best estimate of the asymptotic time. Results annotated with † have been obtained by excluding the edition of Sidney 2000. The exclusion of these performance data is justified by the low level of the participant to the competition of this edition of the Games. For example, about the men 5000 meters of Sidney 2000 the web site `sports-reference.com` writes: "This race was one of survivors and those who chose to run. Notable among those not in the race were world record holder Haile Gebrselassie, 1999 World Champion Saleh Hissou, and top Kenyan distance runner Paul Tergat. The final pace was funereal ...". For the men 200 meters sprint, the web site `sports-reference.com` writes: "This race was expected to be between Americans Maurice Greene and Michael Johnson. Greene was the best in the world at 100 meters and Johnson at 400 meters, and their race in the middle distance was highly anticipated. But neither qualified for the team at the Olympic Trials, succumbing to minor injuries, although they both made the team in their better events....".
