# Supplementary material for: Universality, Limits and Predictability of Gold-Medal Performances at the Olympic Games
Source: PLoS One. 2012 Jul 12;7(7):e40335. doi: 10.1371/journal.pone.0040335 (PMC3395717; doi:10.1371/journal.pone.0040335)
Supplement: Supporting Information S2 — Complete analysis of “field” specialties in athletics. (PDF) [file pone.0040335.s009.pdf]

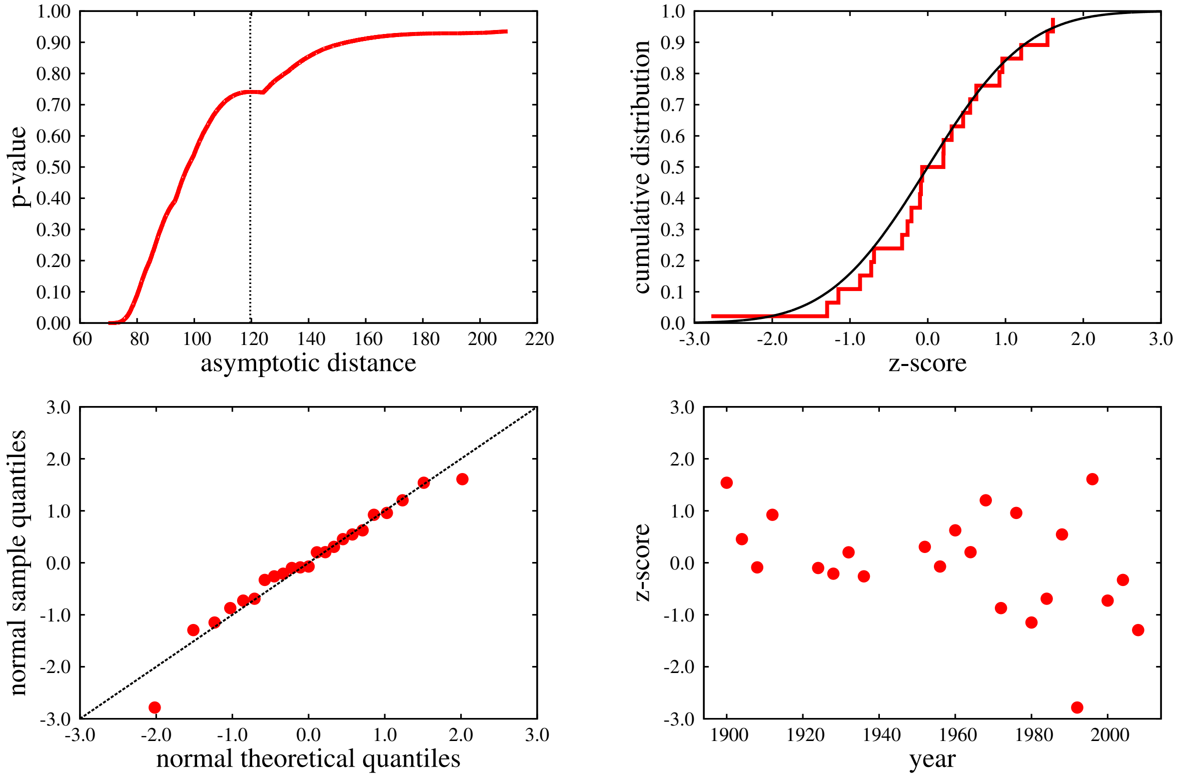

Figure S2.1: Track & Field: men discus throw

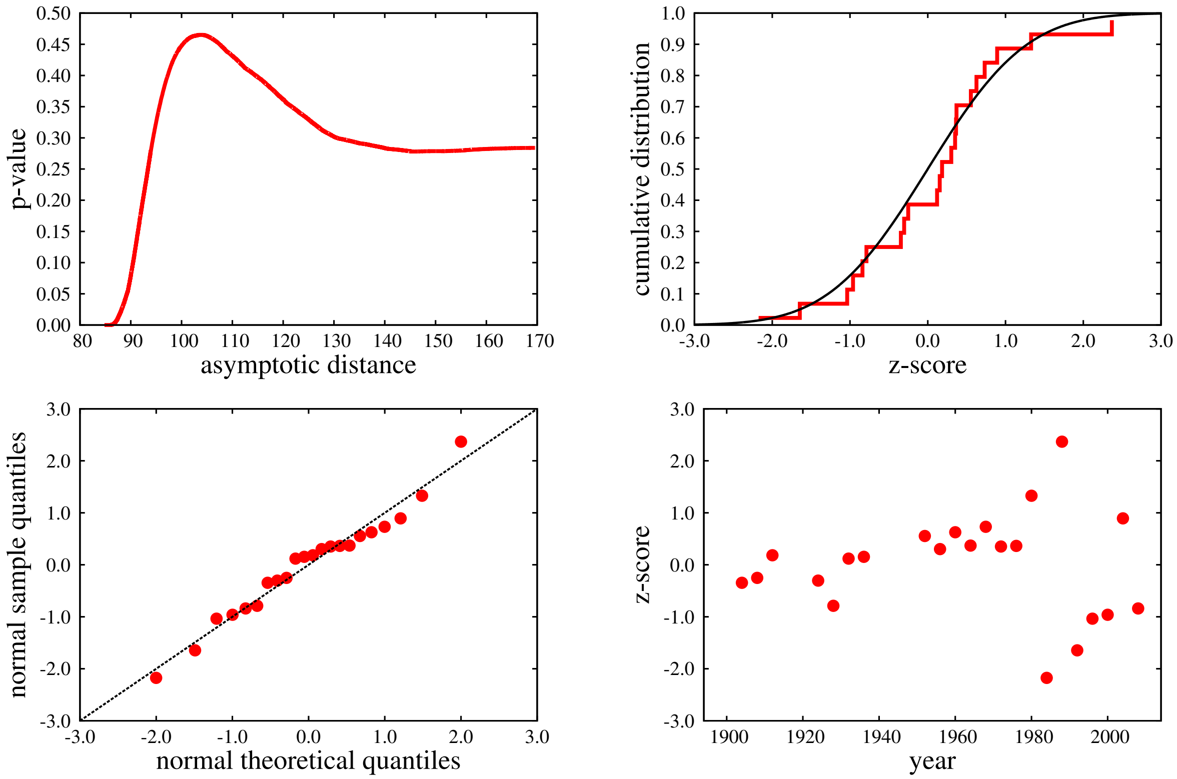

Figure S2.2: Track & Field: men hammer throw

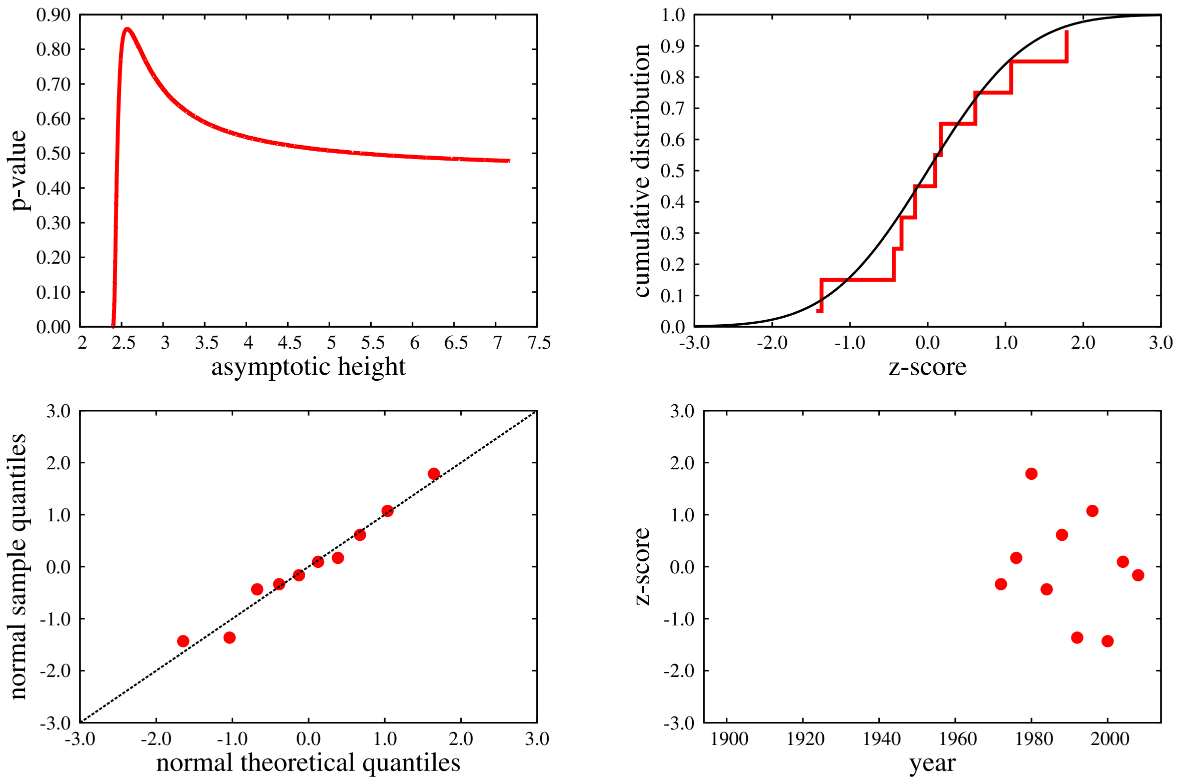

Figure S2.3: Track & Field: men high jump

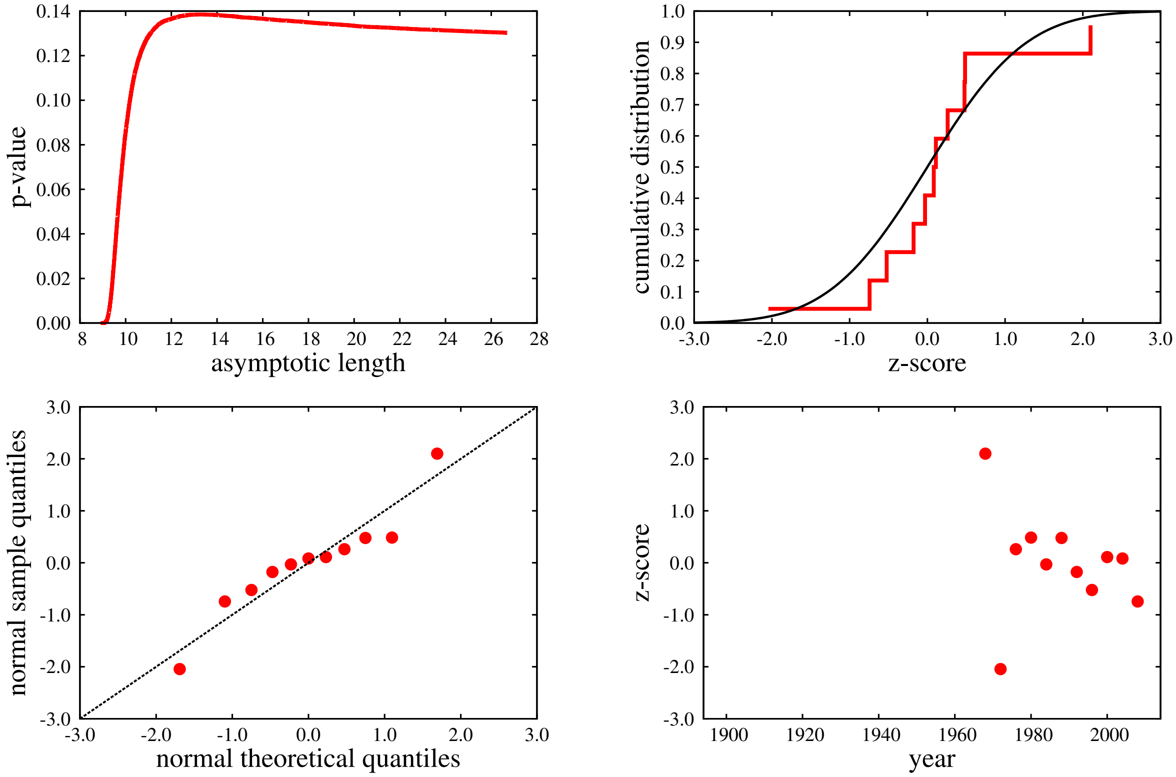

Figure S2.4: Track & Field: men long jump

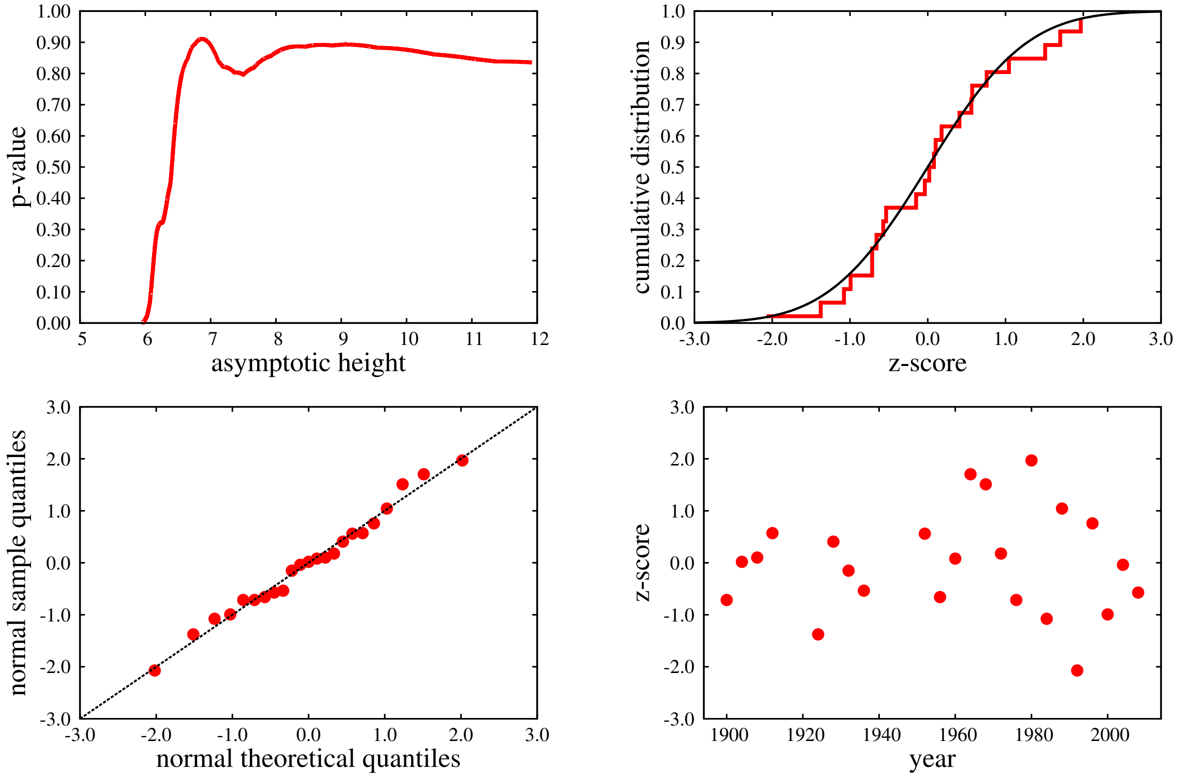

Figure S2.5: Track & Field: men pole vault

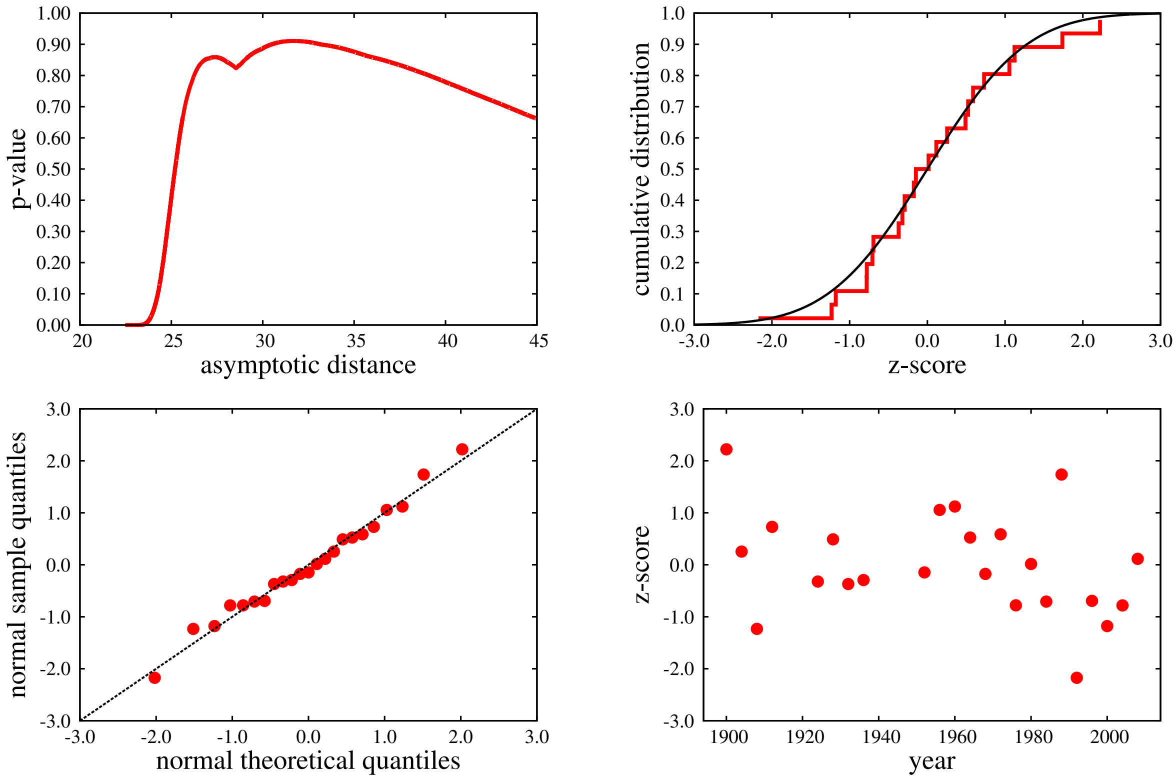

Figure S2.6: Track & Field: men shot put

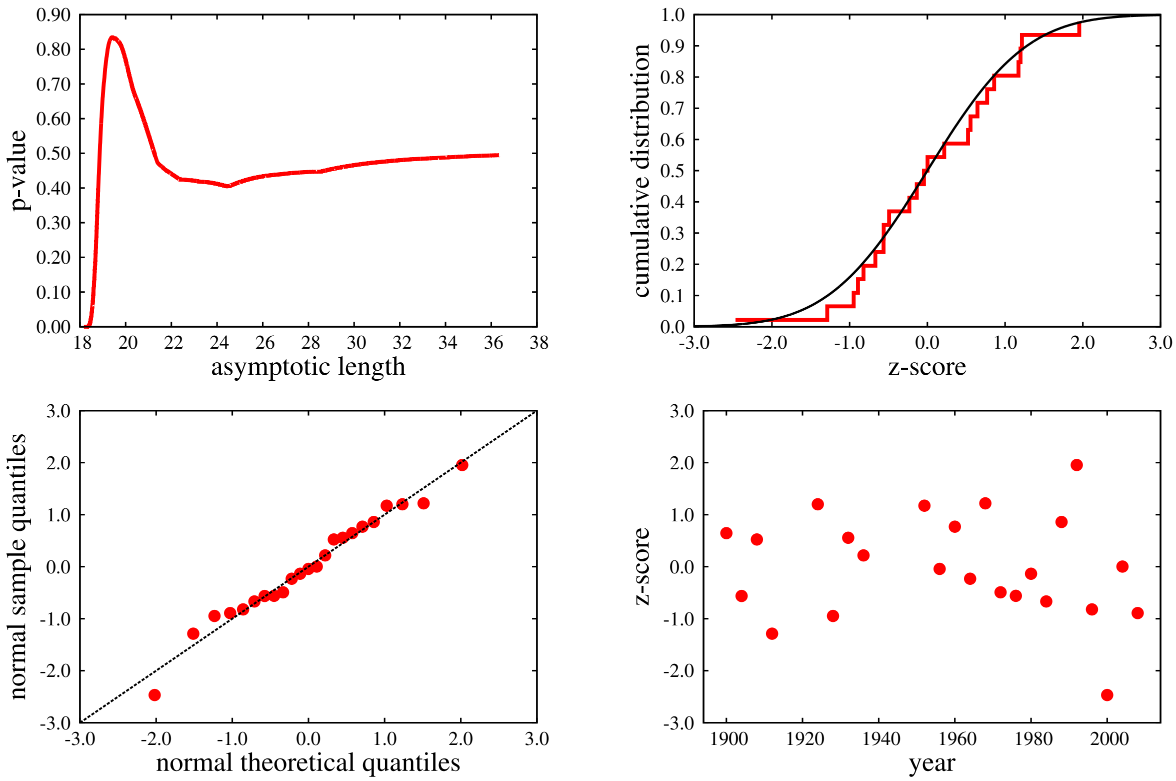

Figure S2.7: Track & Field: men triple jump

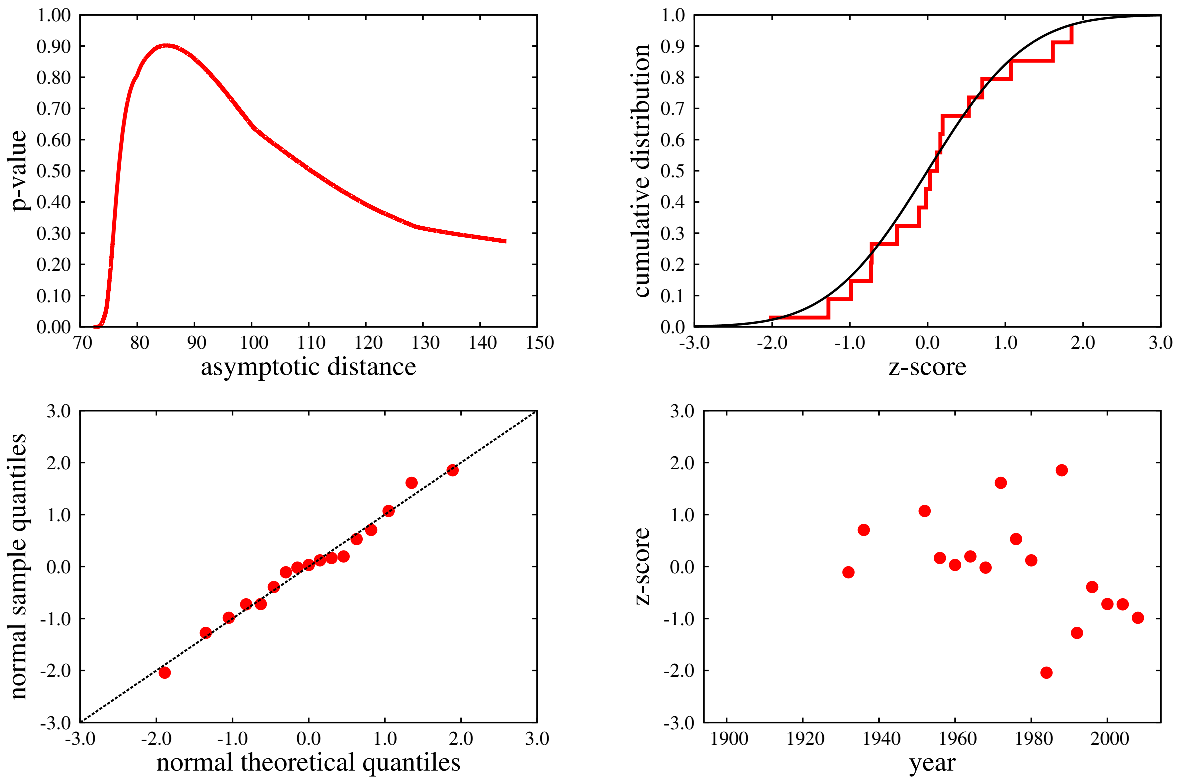

Figure S2.8: Track & Field: women discus throw

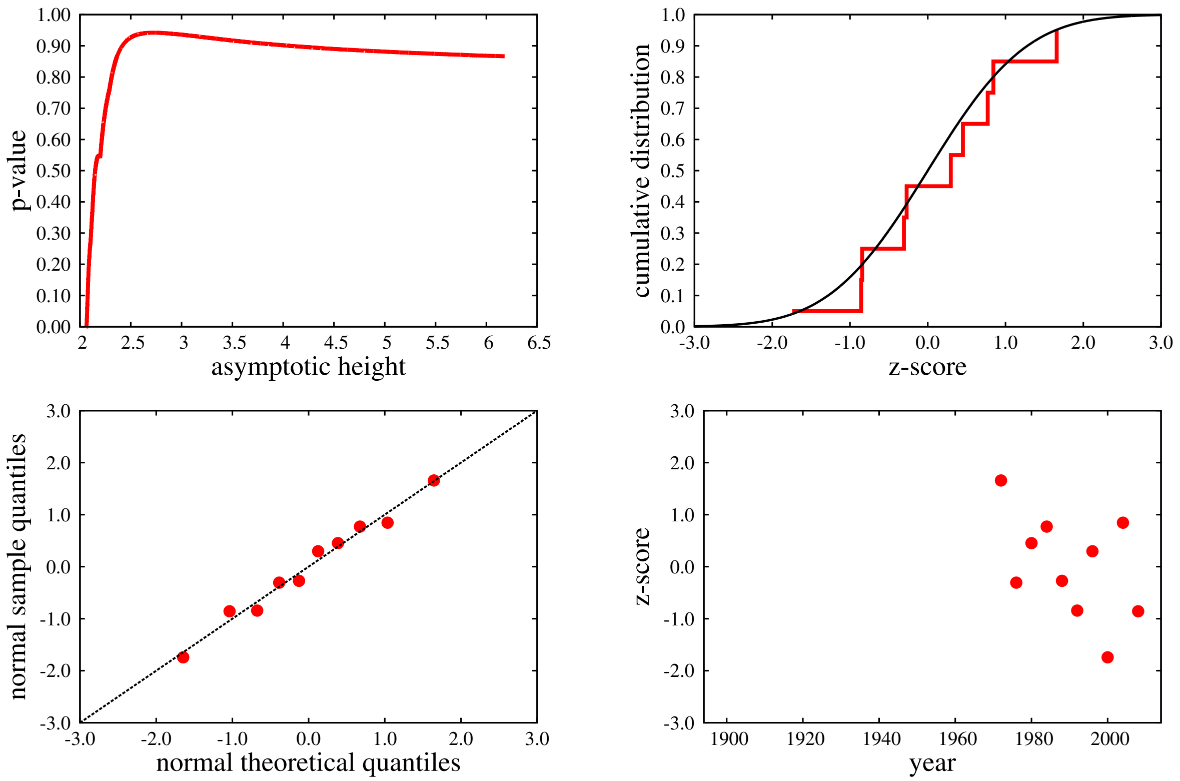

Figure S2.9: Track & Field: women high jump

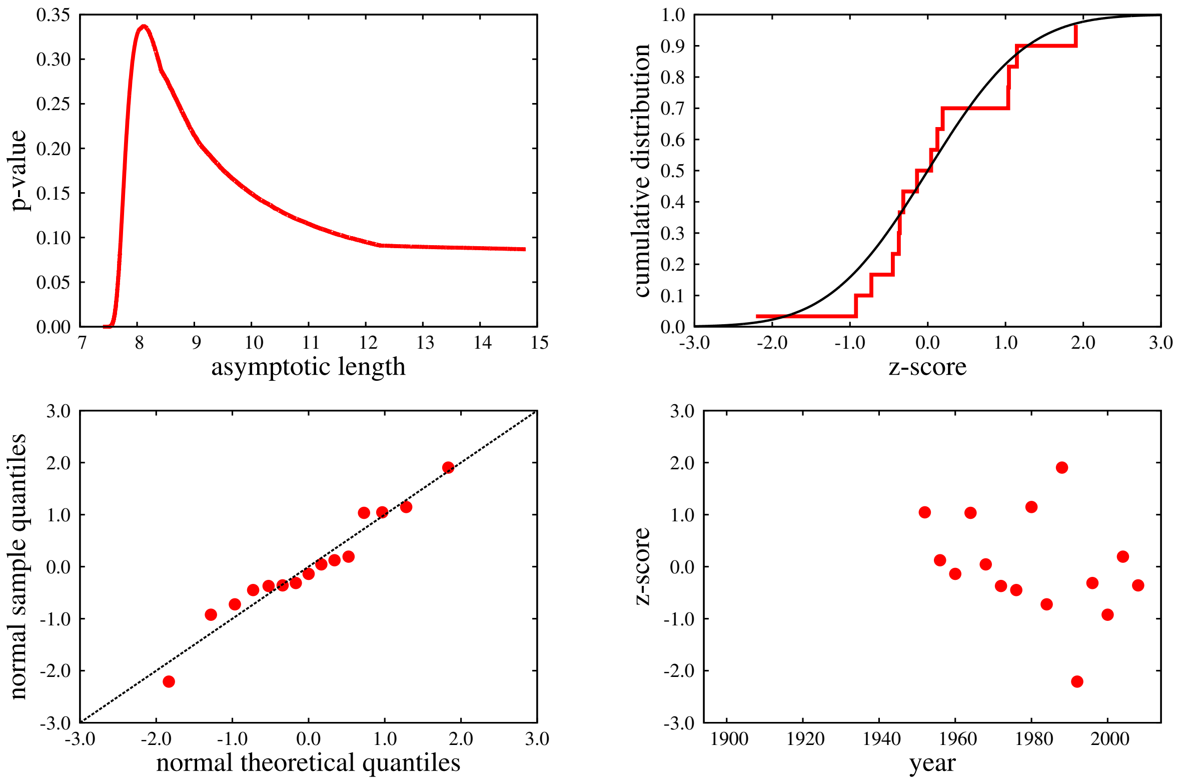

Figure S2.10: Track & Field: women long jump

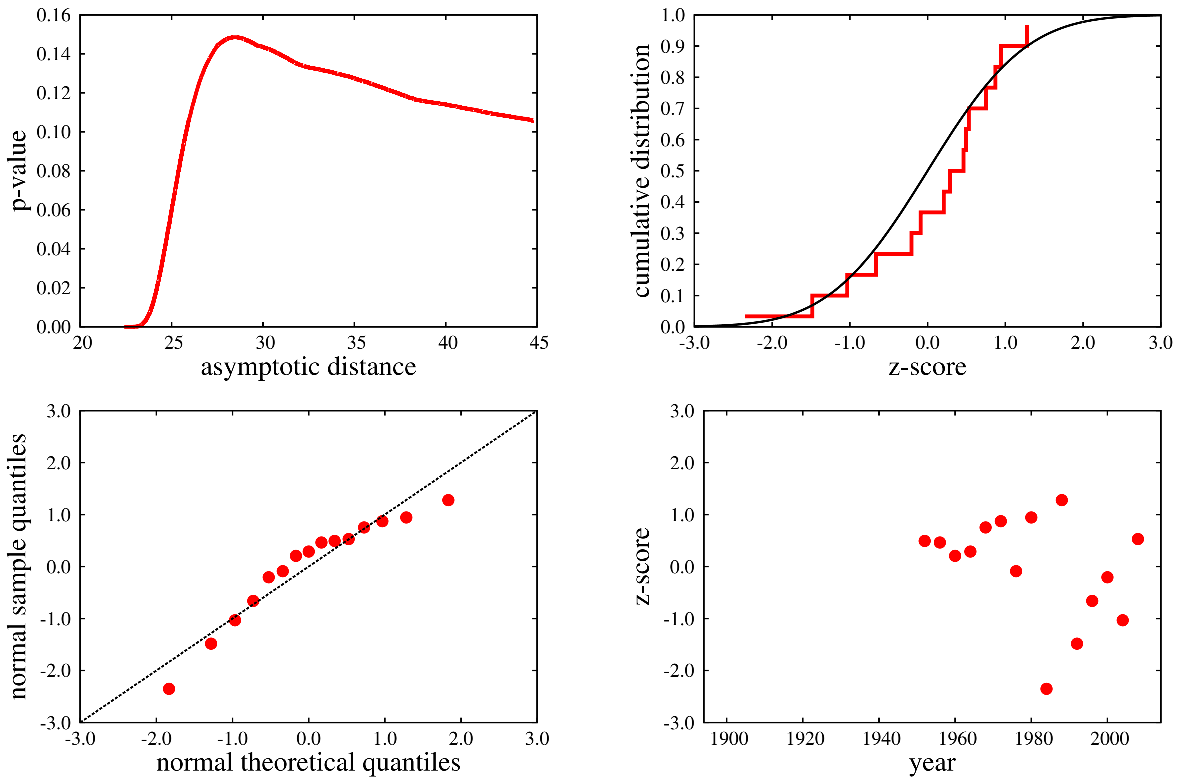

Figure S2.11: Track & Field: women shot put

specialty	$\hat{p}_\infty$	$\hat{\mu}$	$\hat{\sigma}$	A^{*2}	p-value	$\hat{p}_\infty^{(u)}$	nr. events
men discus throw*	119.60	0.03	0.03	0.28	0.74	78.19	26
men hammer throw	103.81	0.04	0.09	0.39	0.47	89.24	25
men high jump†	2.57	0.03	0.18	0.23	0.86	2.41	11
men long jump†	13.28	0.00	0.07	0.62	0.14	9.67	11
men pole vault	6.87	0.05	0.08	0.21	0.91	6.06	26
men shot put	31.70	0.03	0.05	0.21	0.91	24.04	26
men triple jump	19.42	0.05	0.13	0.24	0.83	18.53	26
women discus throw	85.07	0.04	0.17	0.22	0.90	74.51	19
women high jump†	2.73	0.03	0.05	0.19	0.94	2.07	11
women long jump	8.12	0.04	0.18	0.46	0.34	7.67	16
women shot put	28.47	0.03	0.15	0.61	0.15	24.82	16

Table S2.1: Summary table for jumping and throwing specialties in Track & Field. From left to right, we report: the name of the specialty, the best estimate of the asymptotic performance value $\hat{p}_\infty$, the best estimate of the average value $\hat{\mu}$ and standard deviation $\hat{\sigma}$ of performance improvements, the Anderson-Darling distance A^{*2} between sample and theoretical normal distributions, the statistical significance (p-value) of the normal fit, the upper value at 5% significance level of the limiting performance value $\hat{p}_\infty^{(u)}$, and the number of Olympic games that included the specialty. Unless specified, the values of $\hat{\mu}$, $\hat{\sigma}$, A^{*2} and p-value have been calculated at $p_\infty = \hat{p}_\infty$. Results annotated with † have been obtained by excluding the edition prior Mexico City 1968. Results annotated with * have been obtained by identifying the best estimate of the asymptotic performance value as a local maximum of the p-value.
