# Supplementary material for: Universality, Limits and Predictability of Gold-Medal Performances at the Olympic Games
Source: PLoS One. 2012 Jul 12;7(7):e40335. doi: 10.1371/journal.pone.0040335 (PMC3395717; doi:10.1371/journal.pone.0040335)
Supplement: Supporting Information S3 — Complete analysis of swimming specialties. (PDF) [file pone.0040335.s010.pdf]

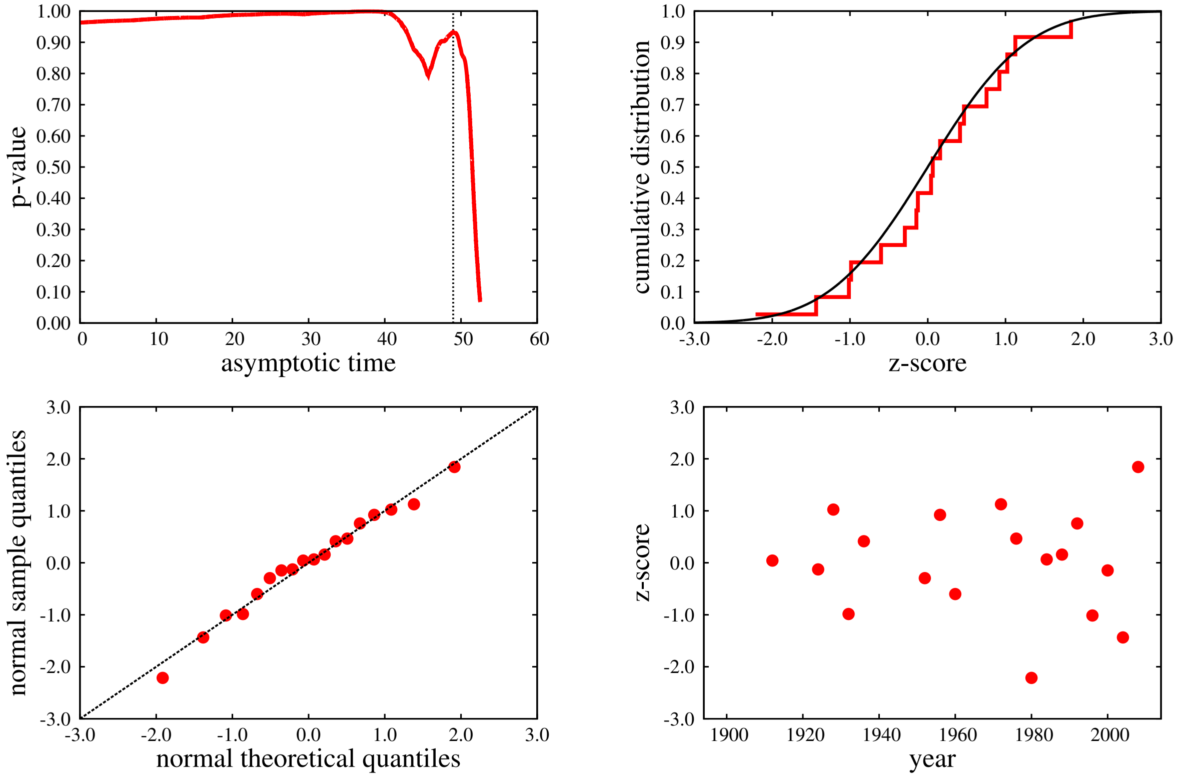

Figure S3.1: Swimming: men 100 meters backstroke

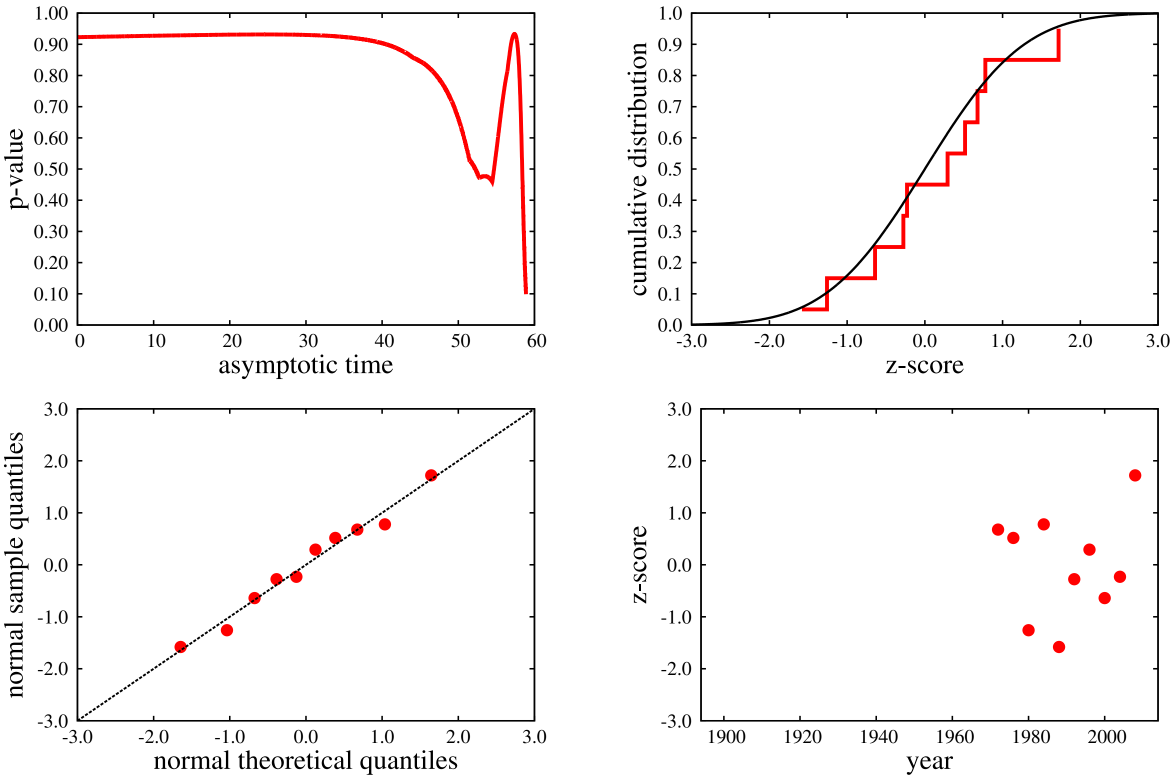

Figure S3.2: Swimming: men 100 meters breaststroke

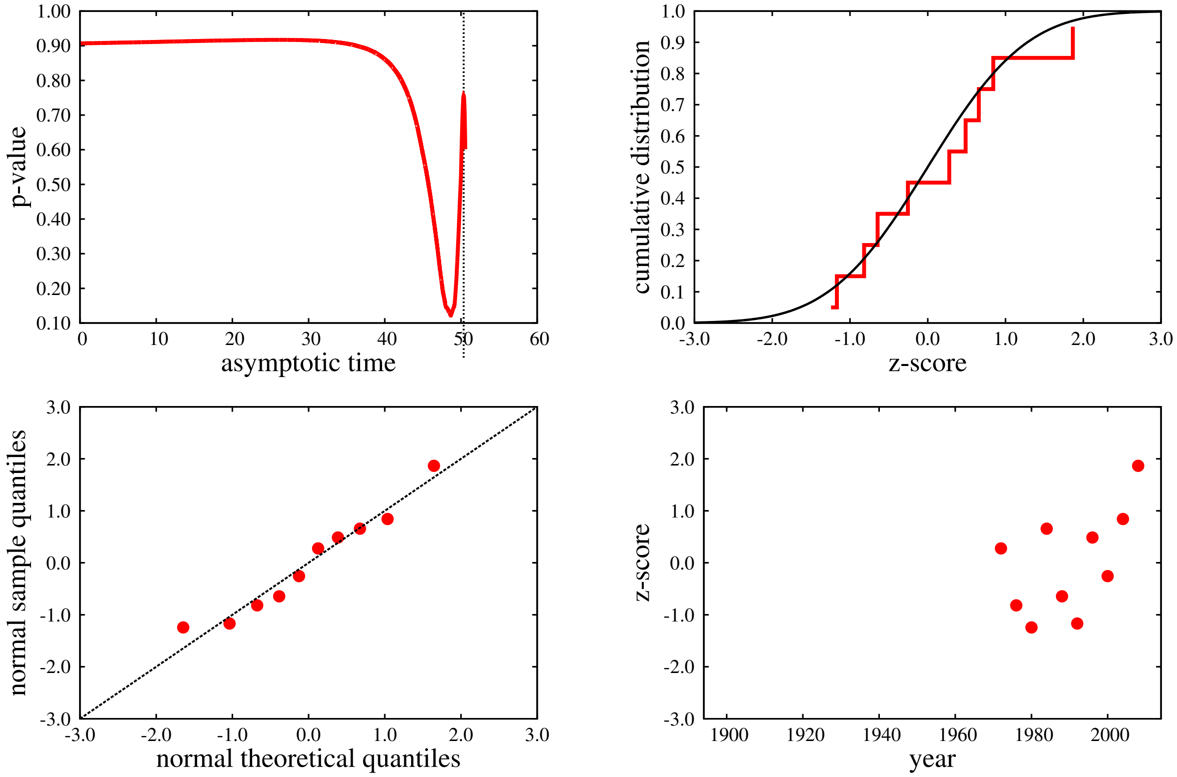

Figure S3.3: Swimming: men 100 meters butterfly

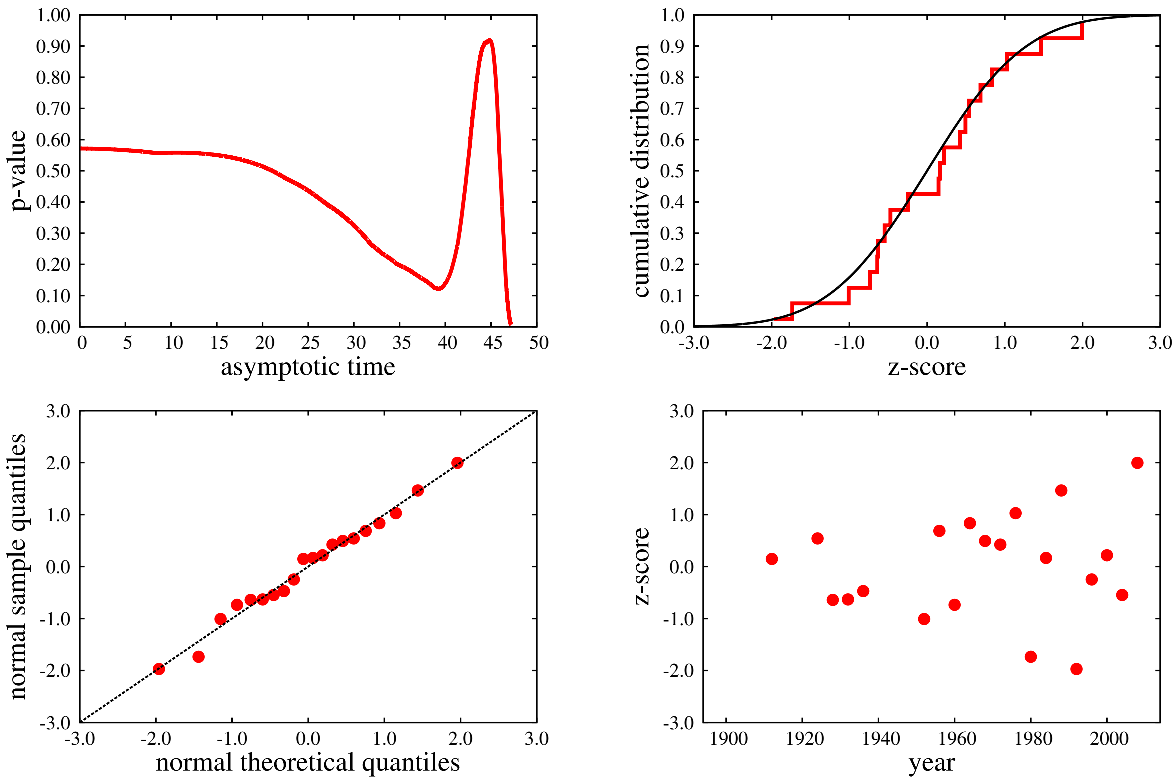

Figure S3.4: Swimming: men 100 meters freestyle

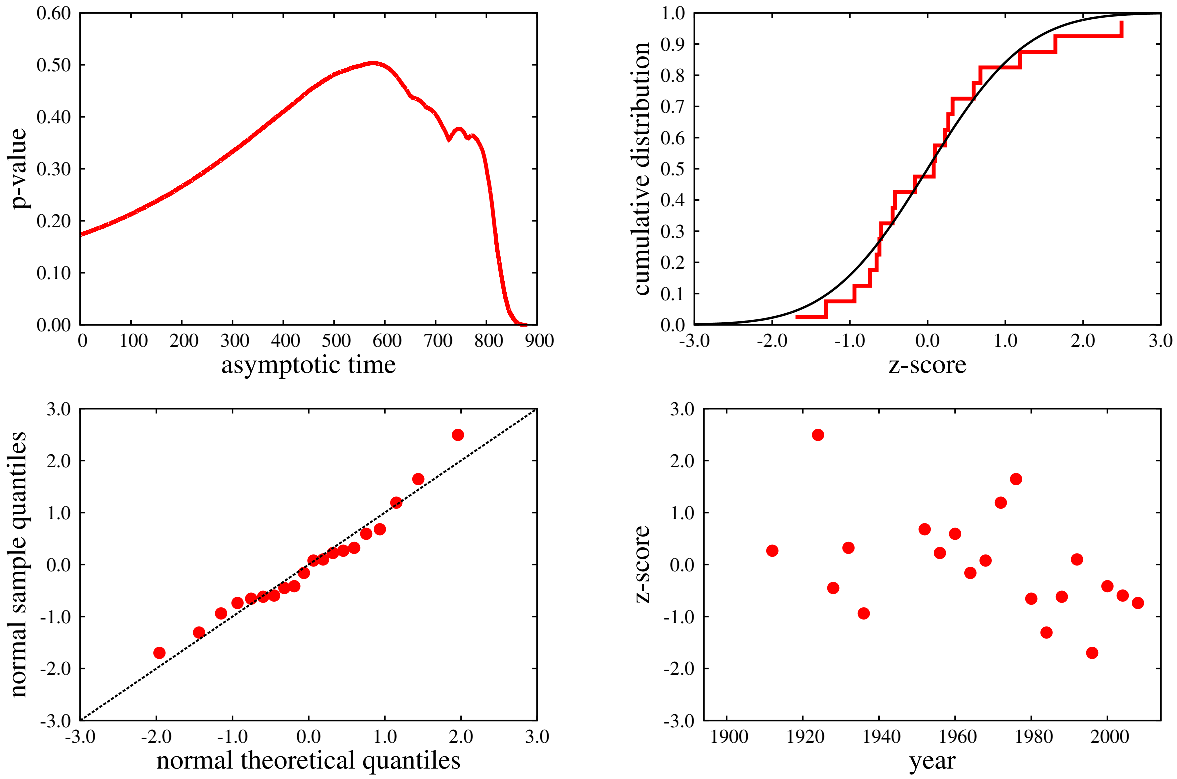

Figure S3.5: Swimming: men 1500 meters freestyle

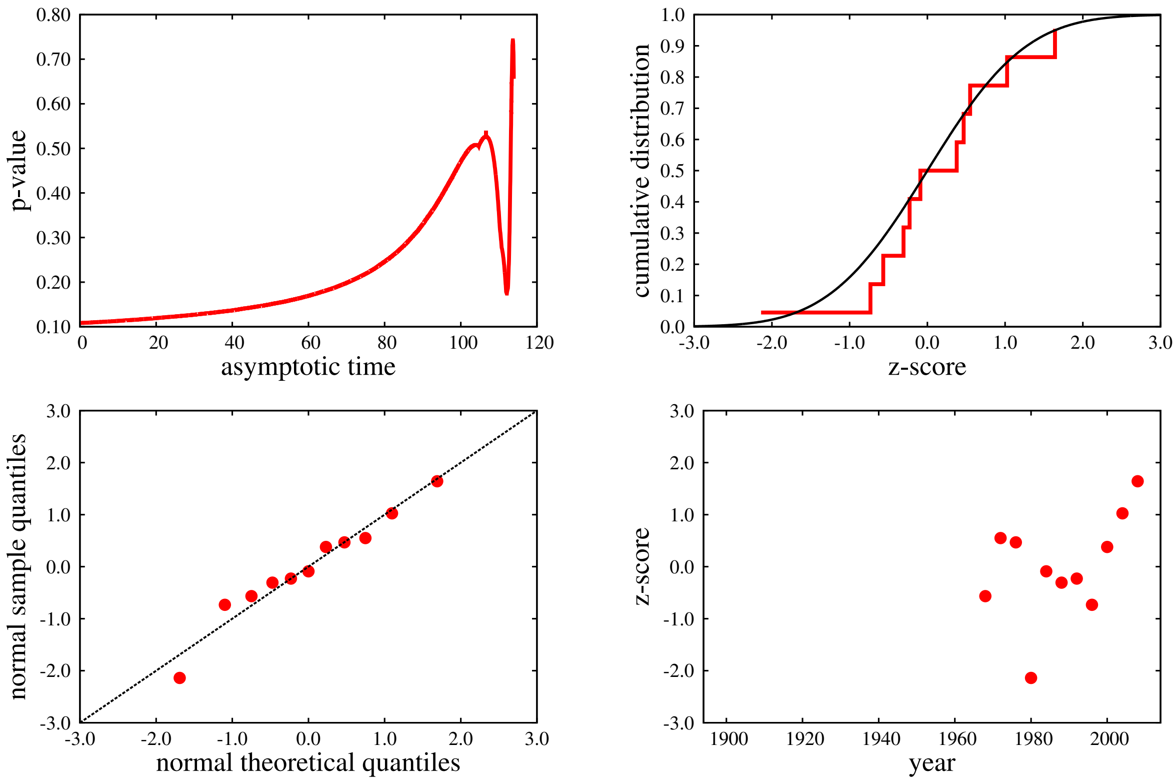

Figure S3.6: Swimming: men 200 meters backstroke

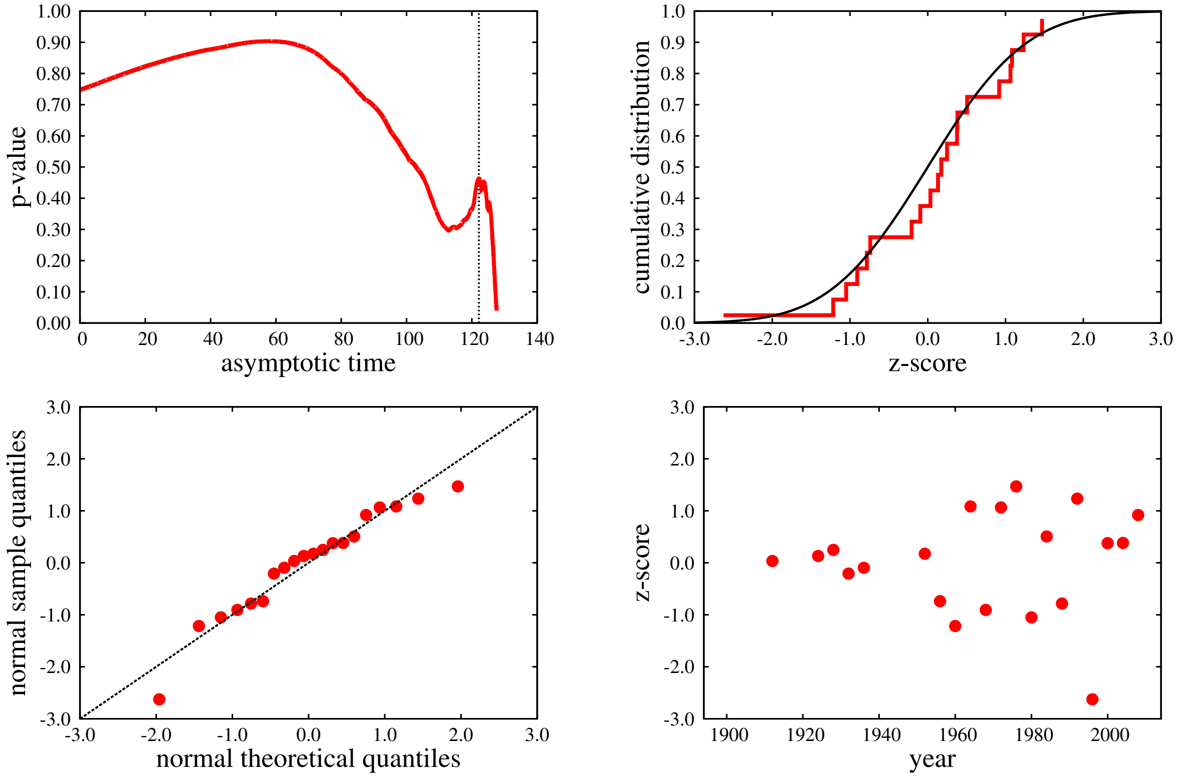

Figure S3.7: Swimming: men 200 meters breaststroke

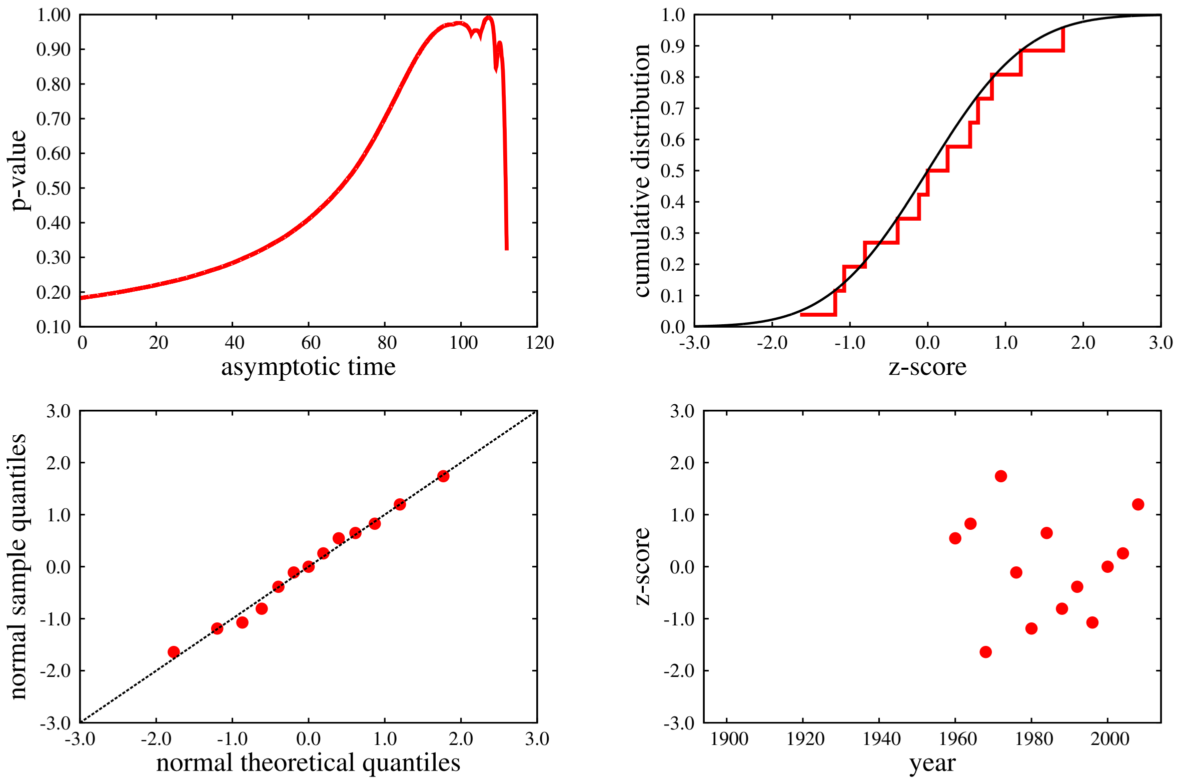

Figure S3.8: Swimming: men 200 meters butterfly

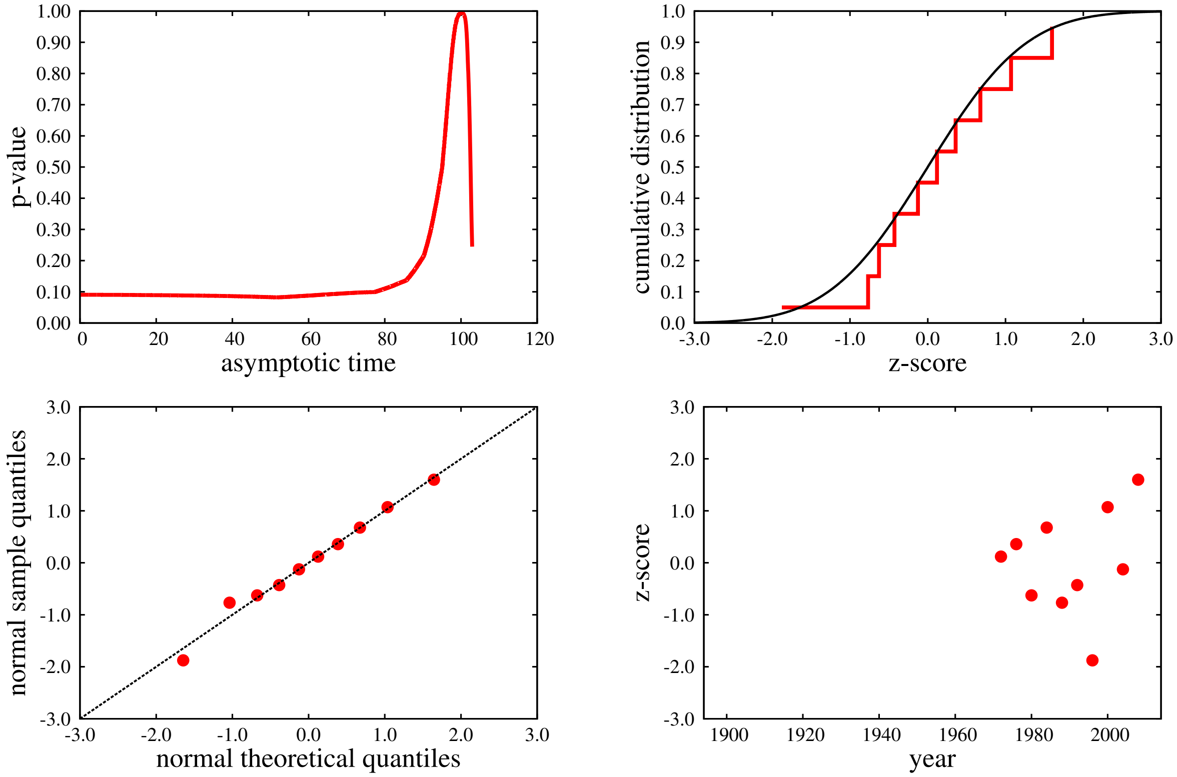

Figure S3.9: Swimming: men 200 meters freestyle

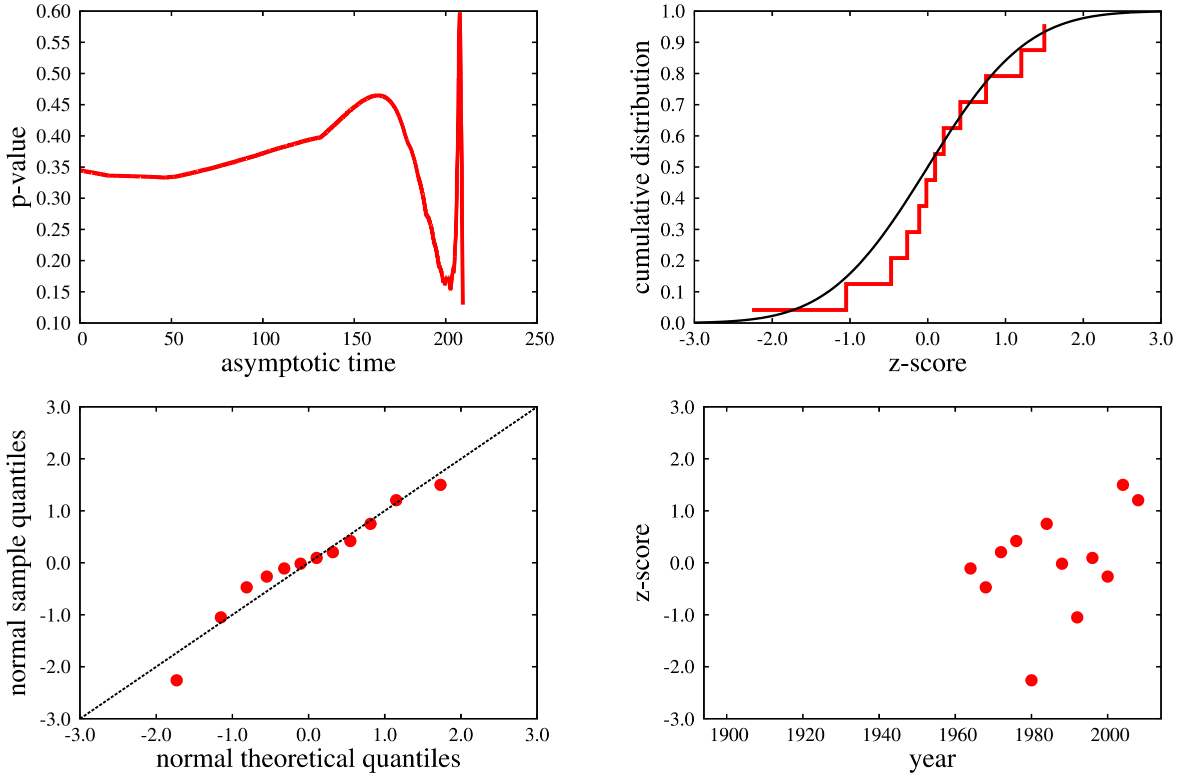

Figure S3.10: Swimming: men 4 x 100 meters medley relay

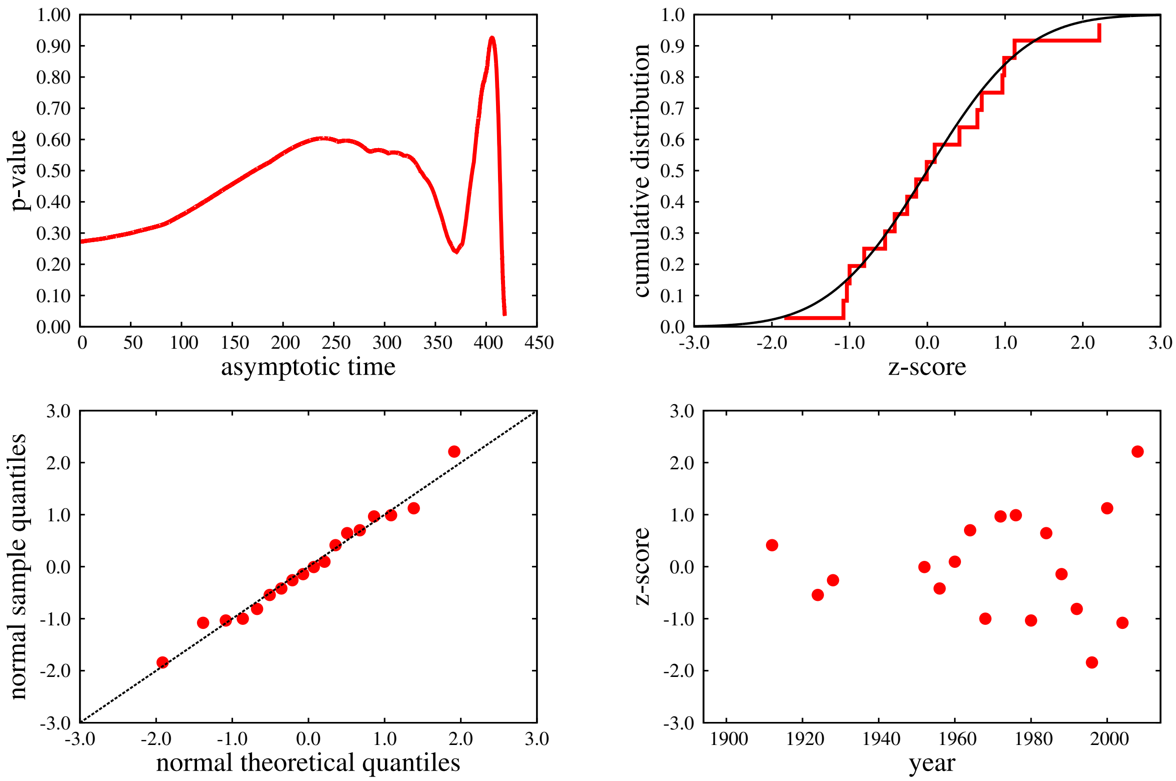

Figure S3.11: Swimming: men 4 x 200 meters freestyle relay

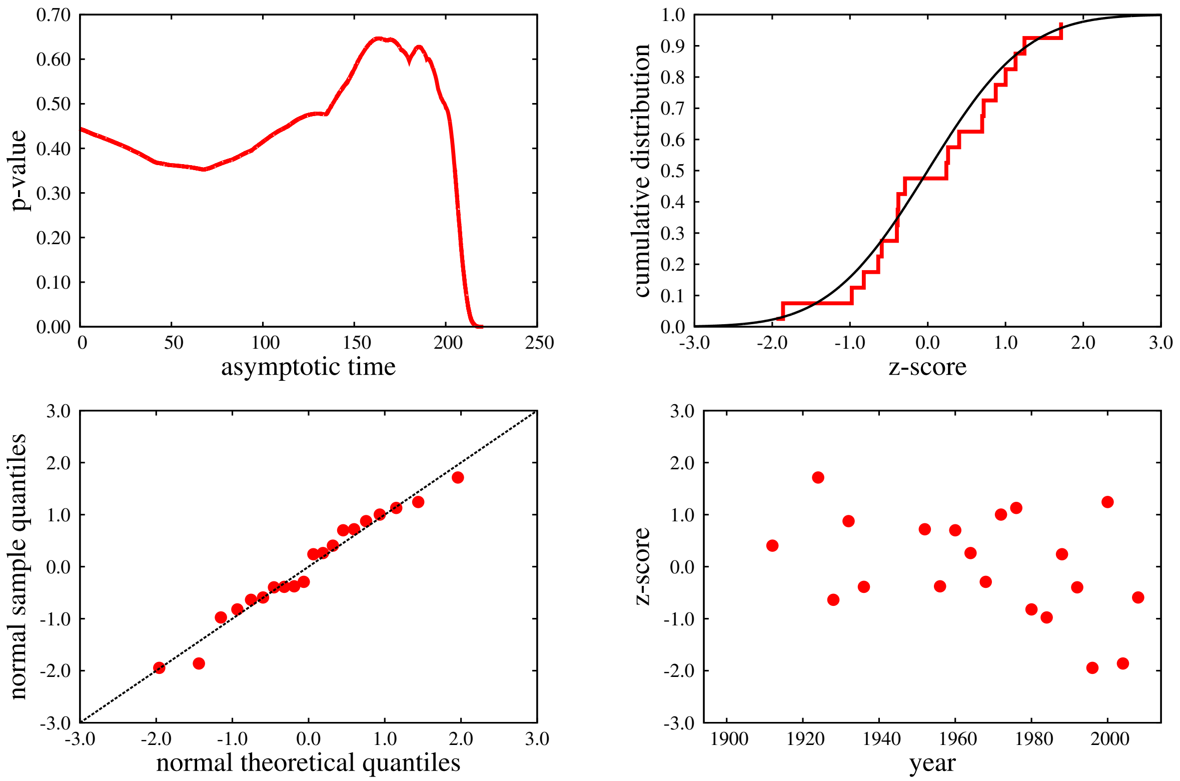

Figure S3.12: Swimming: men 400 meters freestyle

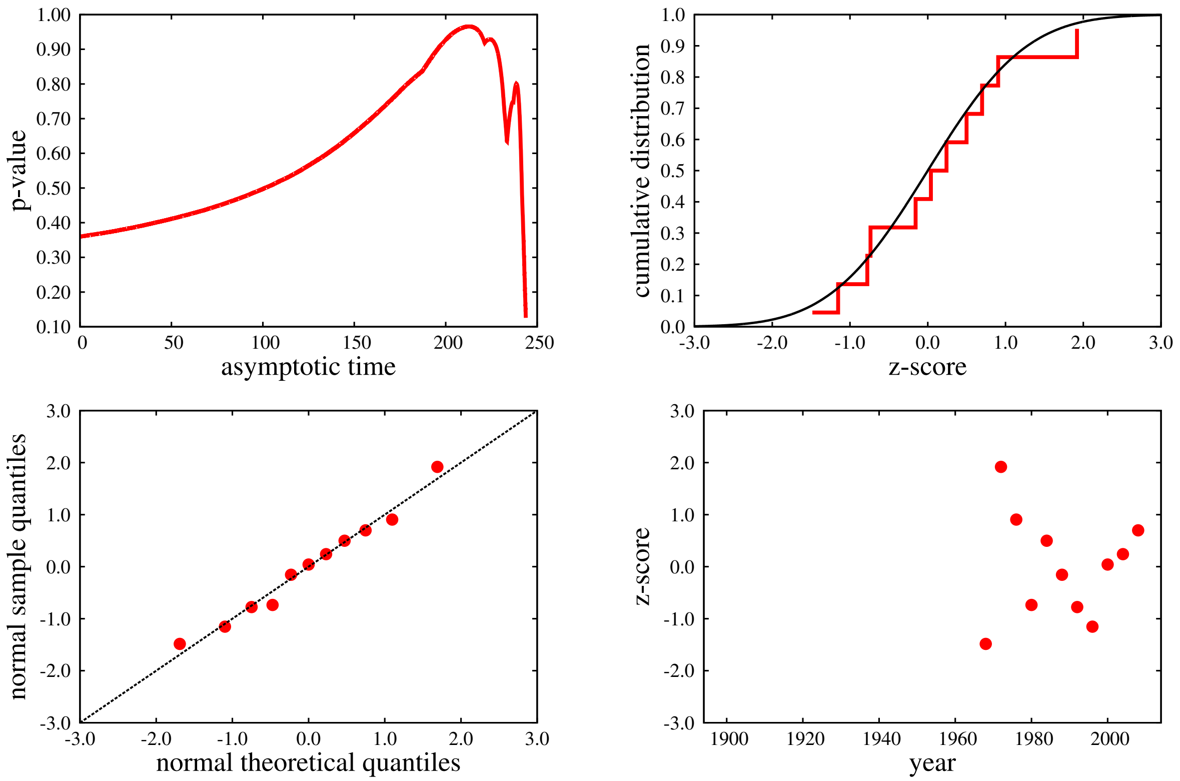

Figure S3.13: Swimming: men 400 meters individual medley

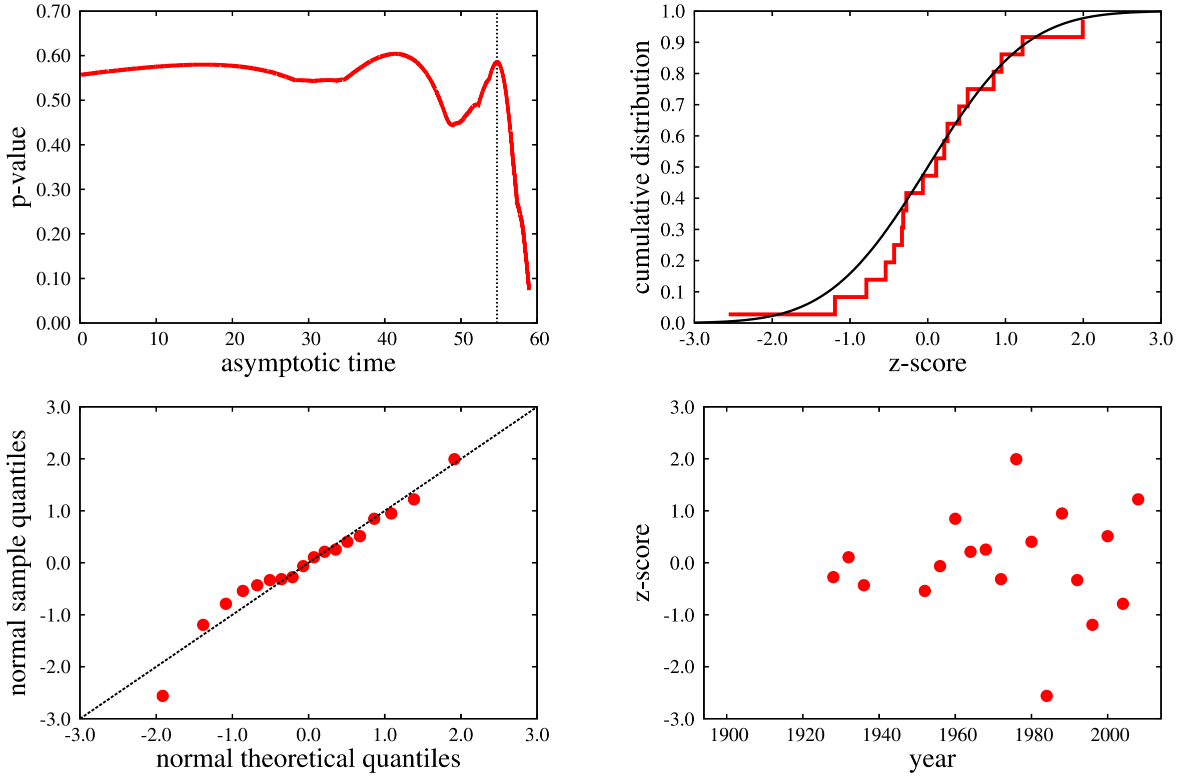

Figure S3.14: Swimming: women 100 meters backstroke

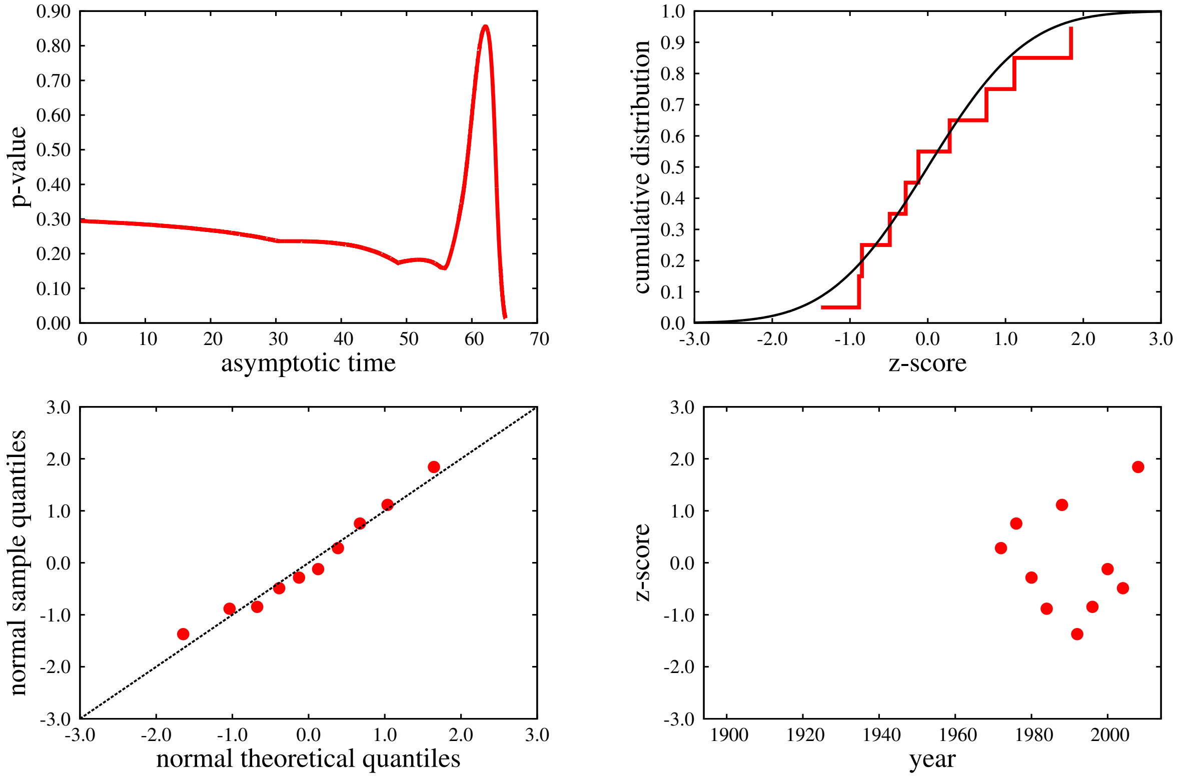

Figure S3.15: Swimming: women 100 meters breaststroke

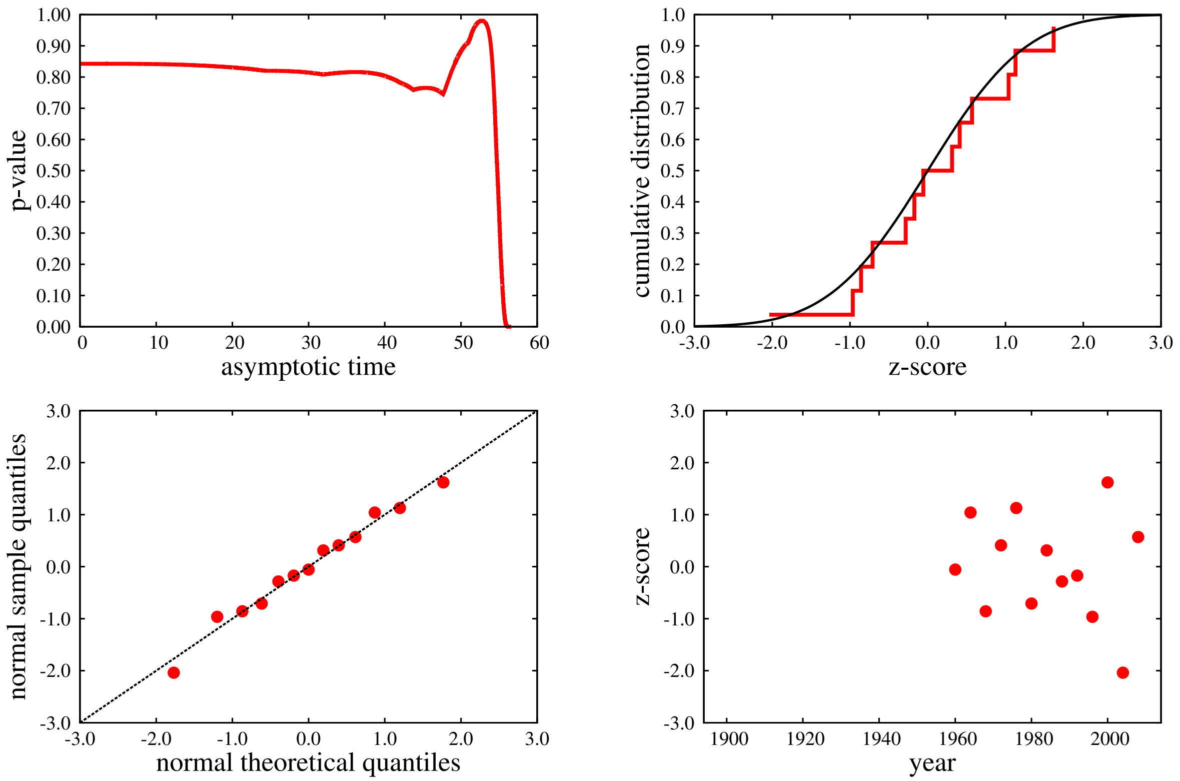

Figure S3.16: Swimming: women 100 meters butterfly

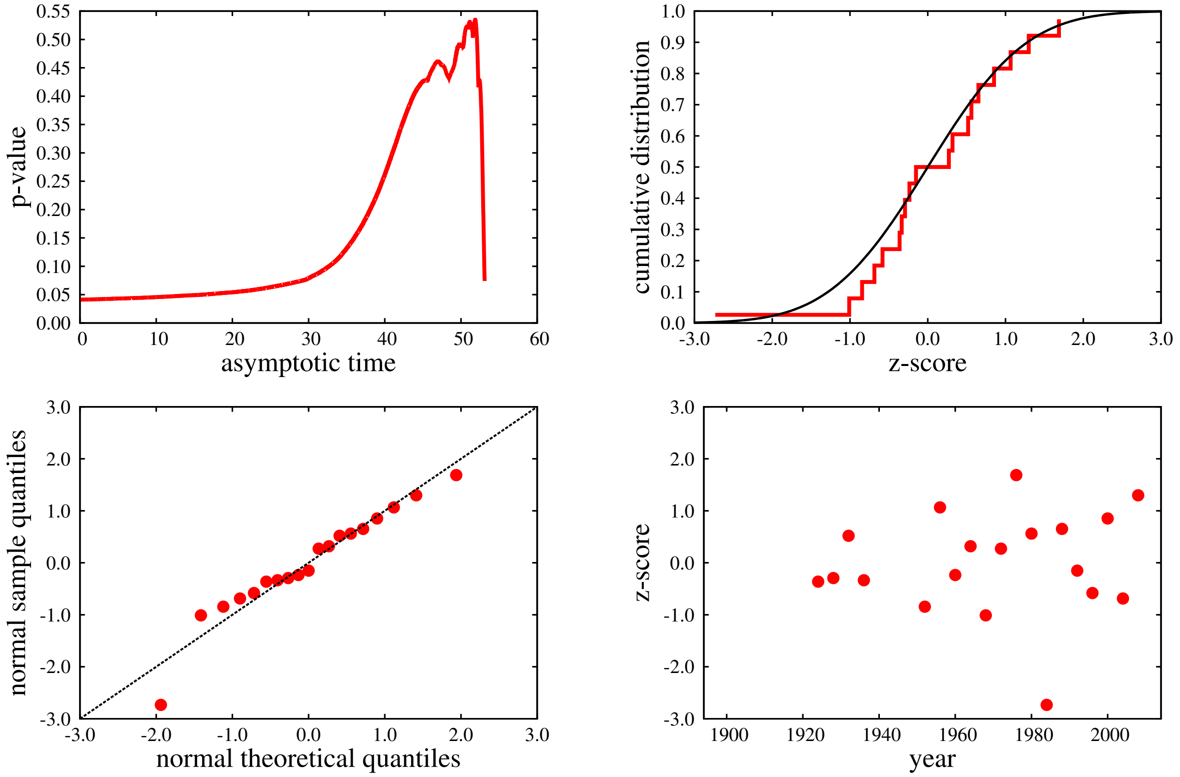

Figure S3.17: Swimming: women 100 meters freestyle

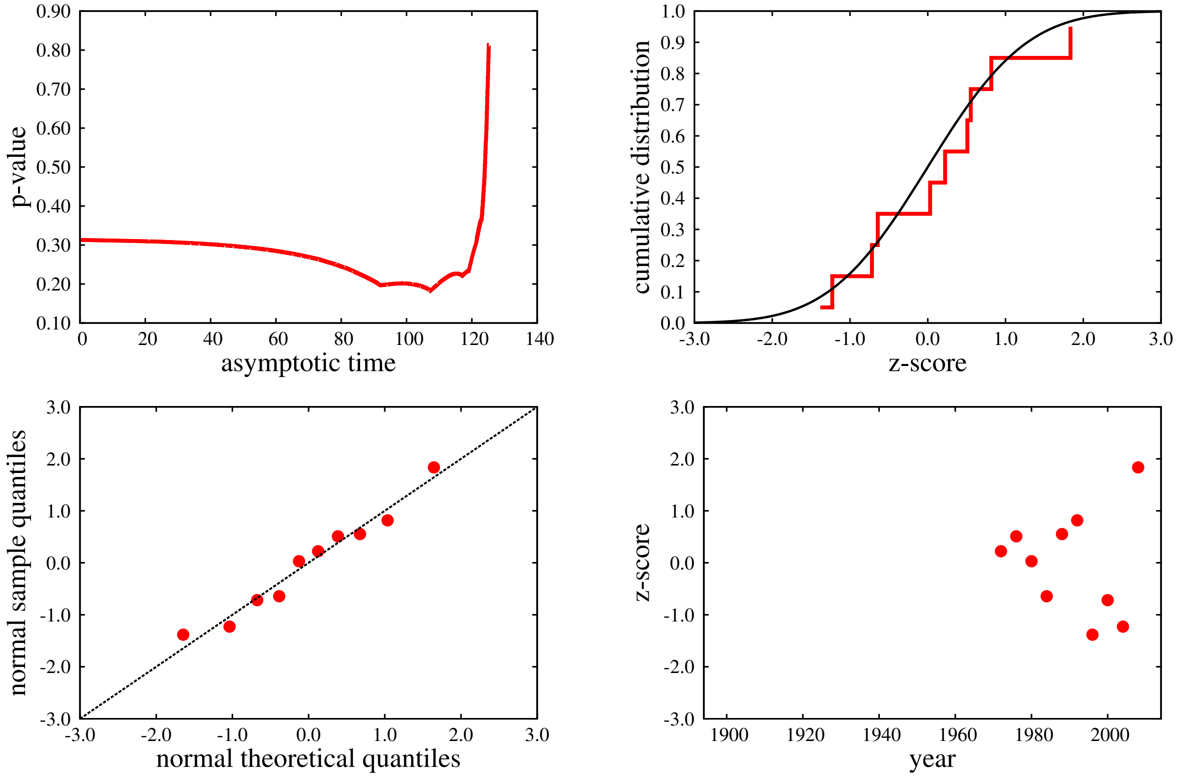

Figure S3.18: Swimming: women 200 meters backstroke

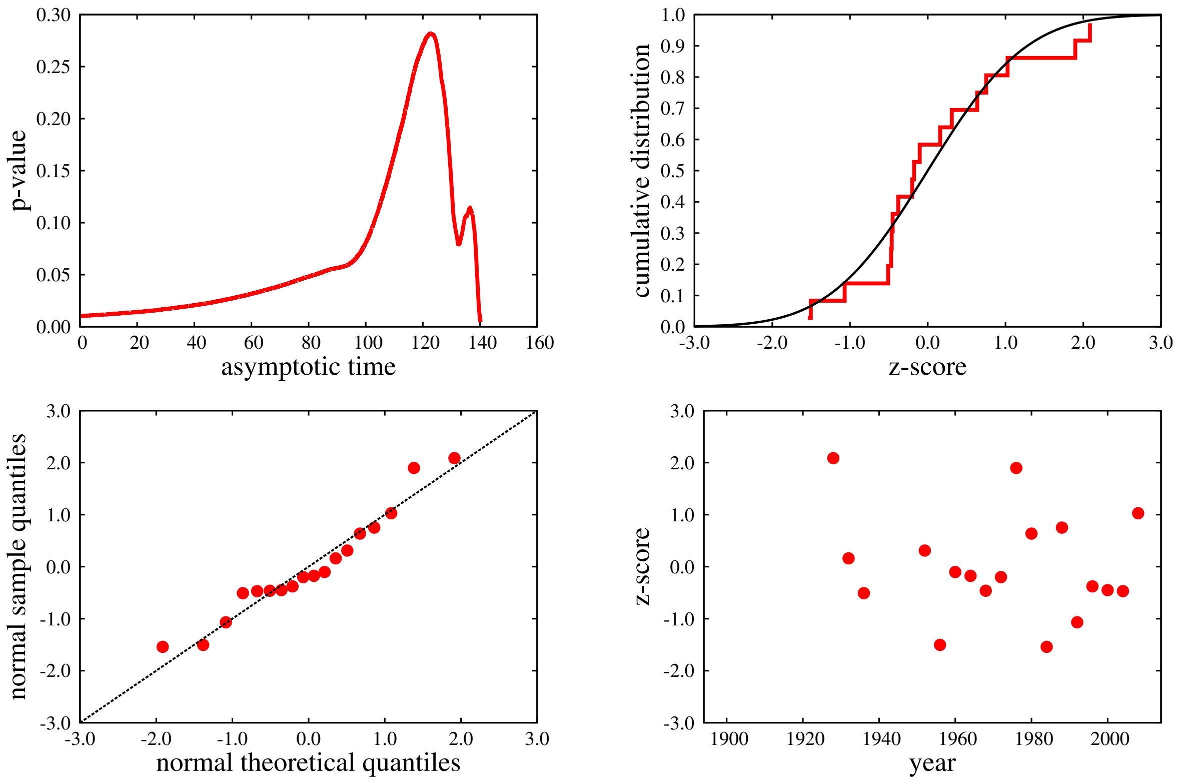

Figure S3.19: Swimming: women 200 meters breaststroke

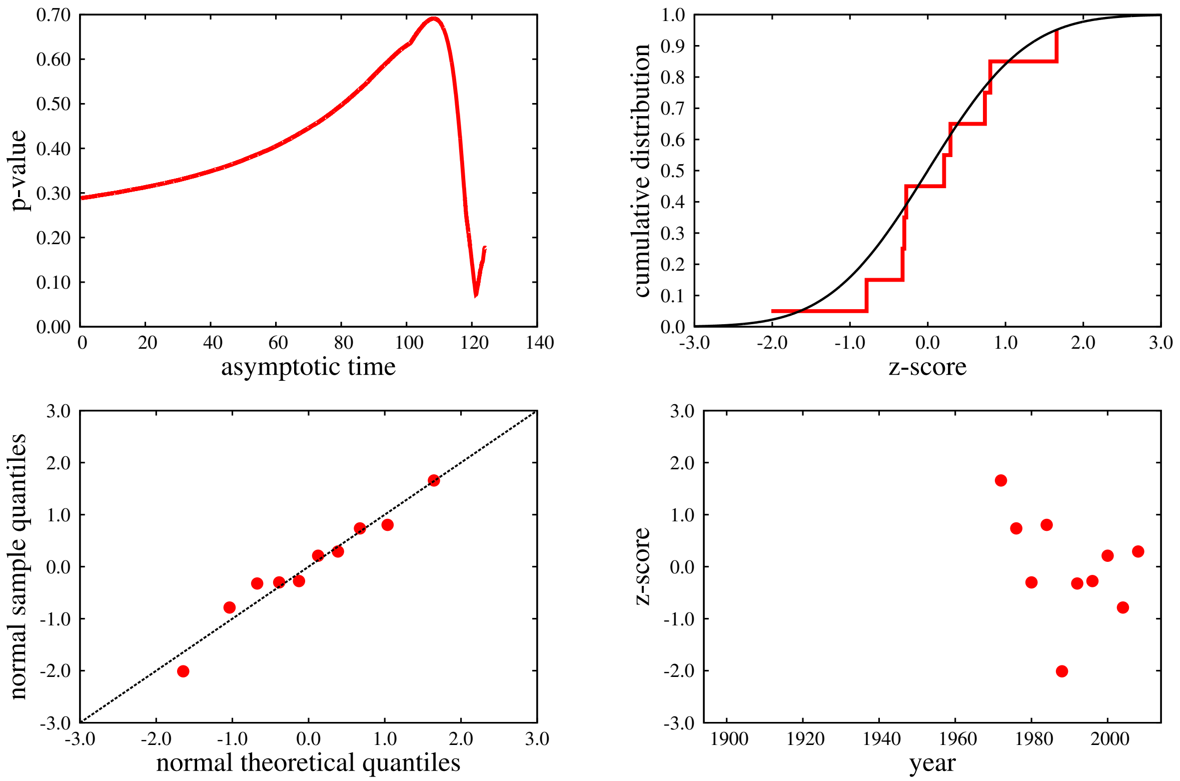

Figure S3.20: Swimming: women 200 meters butterfly

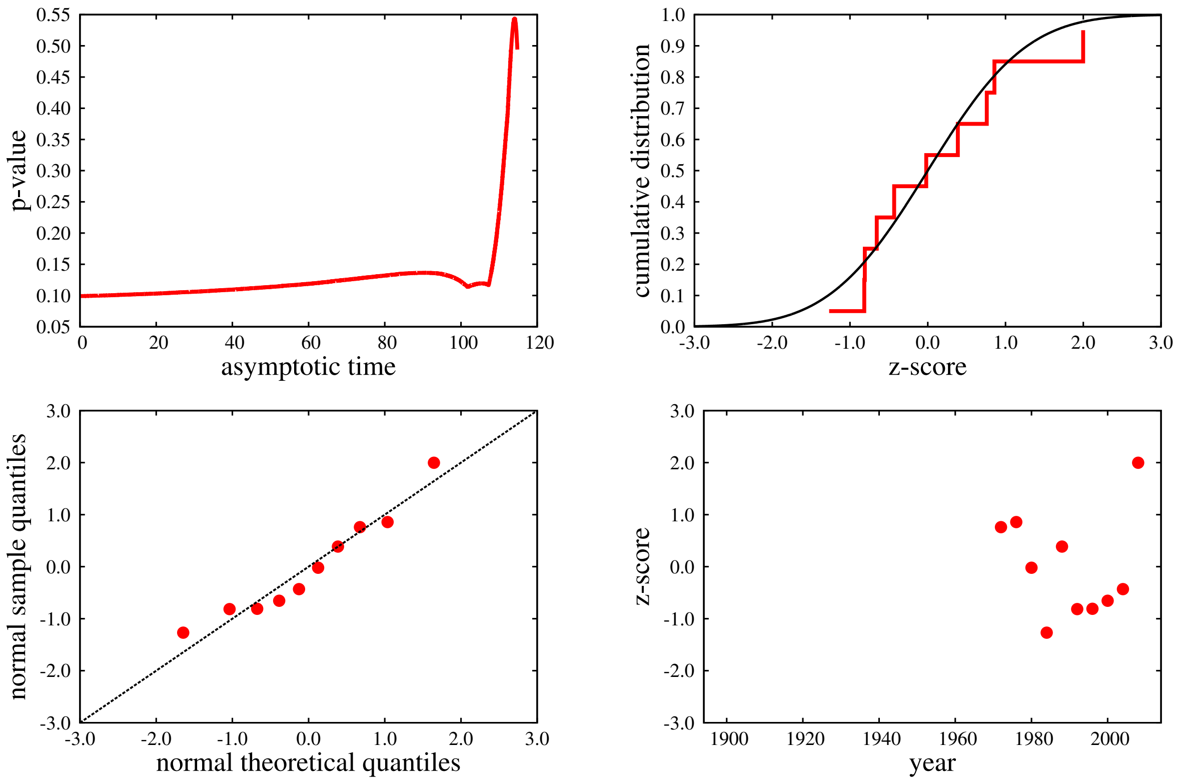

Figure S3.21: Swimming: women 200 meters freestyle

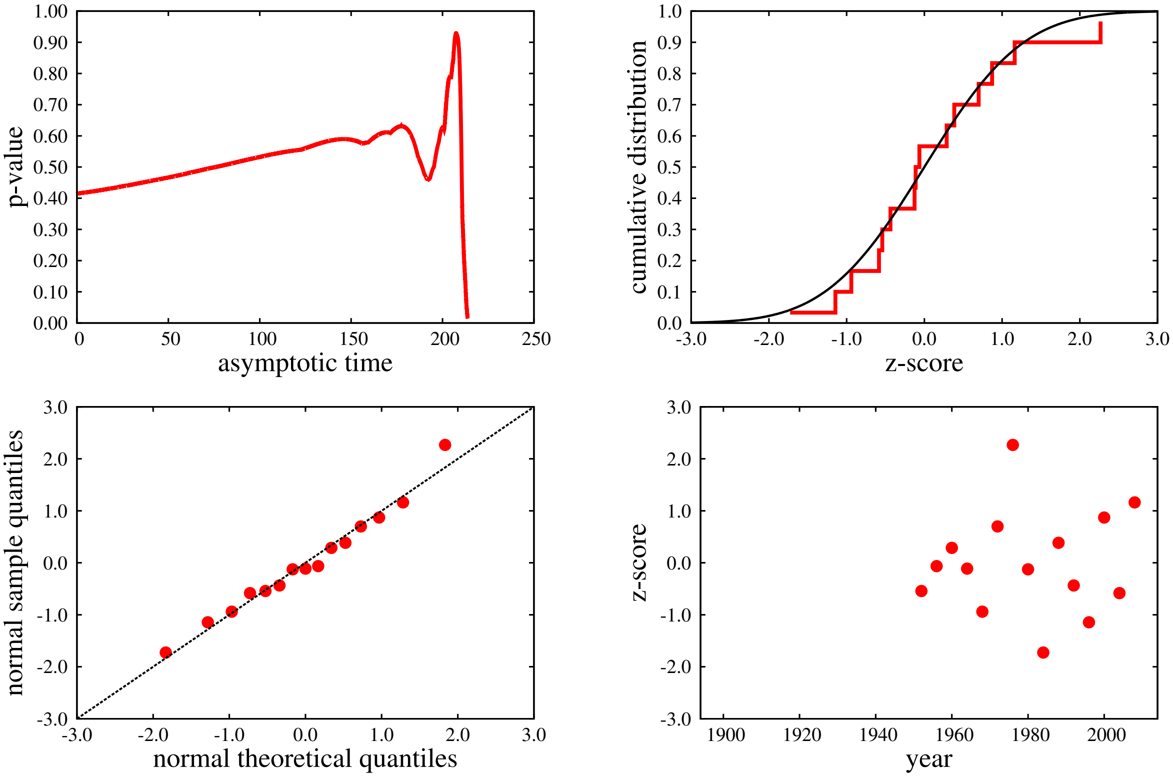

Figure S3.22: Swimming: women 4 x 100 meters freestyle relay

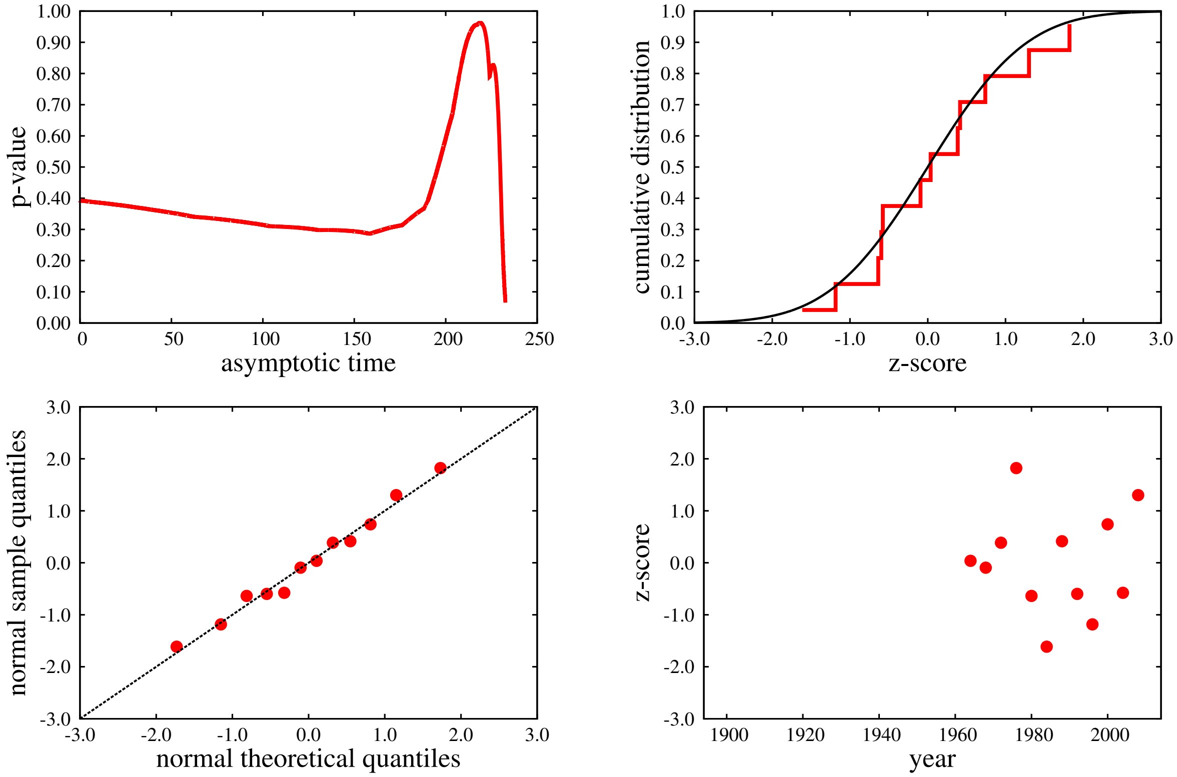

Figure S3.23: Swimming: women 4 x 100 meters medley relay

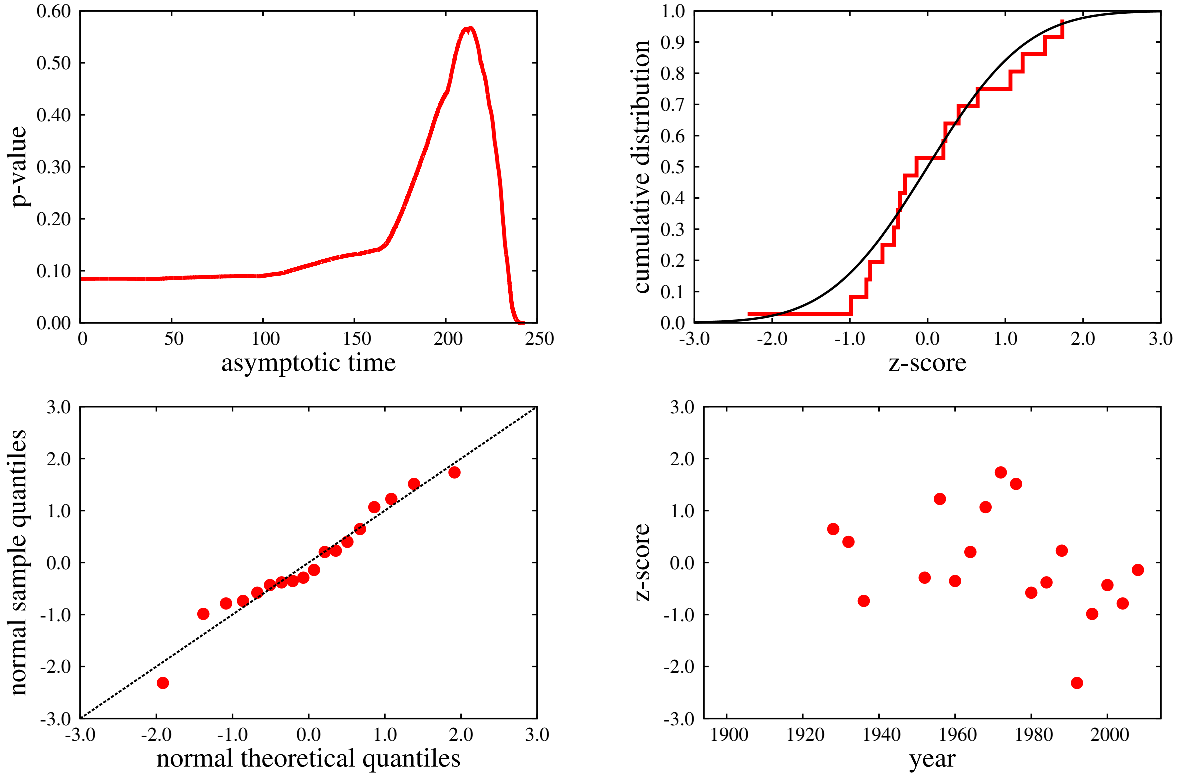

Figure S3.24: Swimming: women 400 meters freestyle

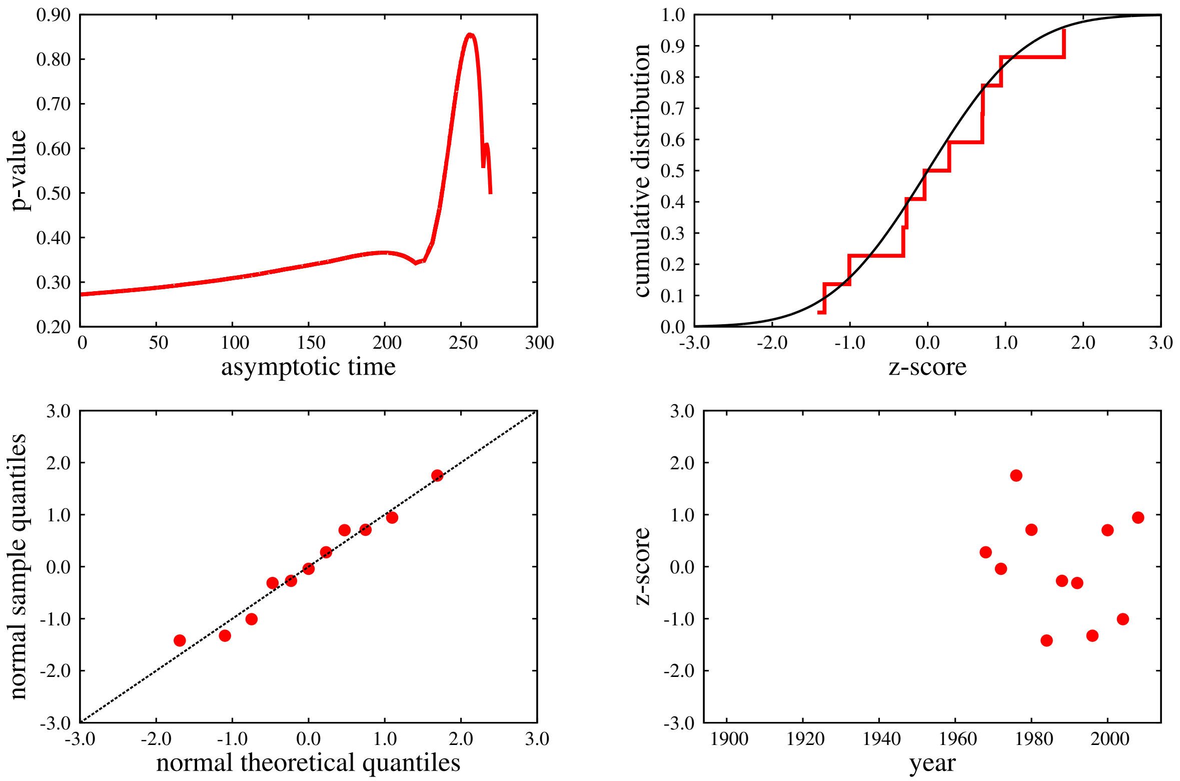

Figure S3.25: Swimming: women 400 meters individual medley

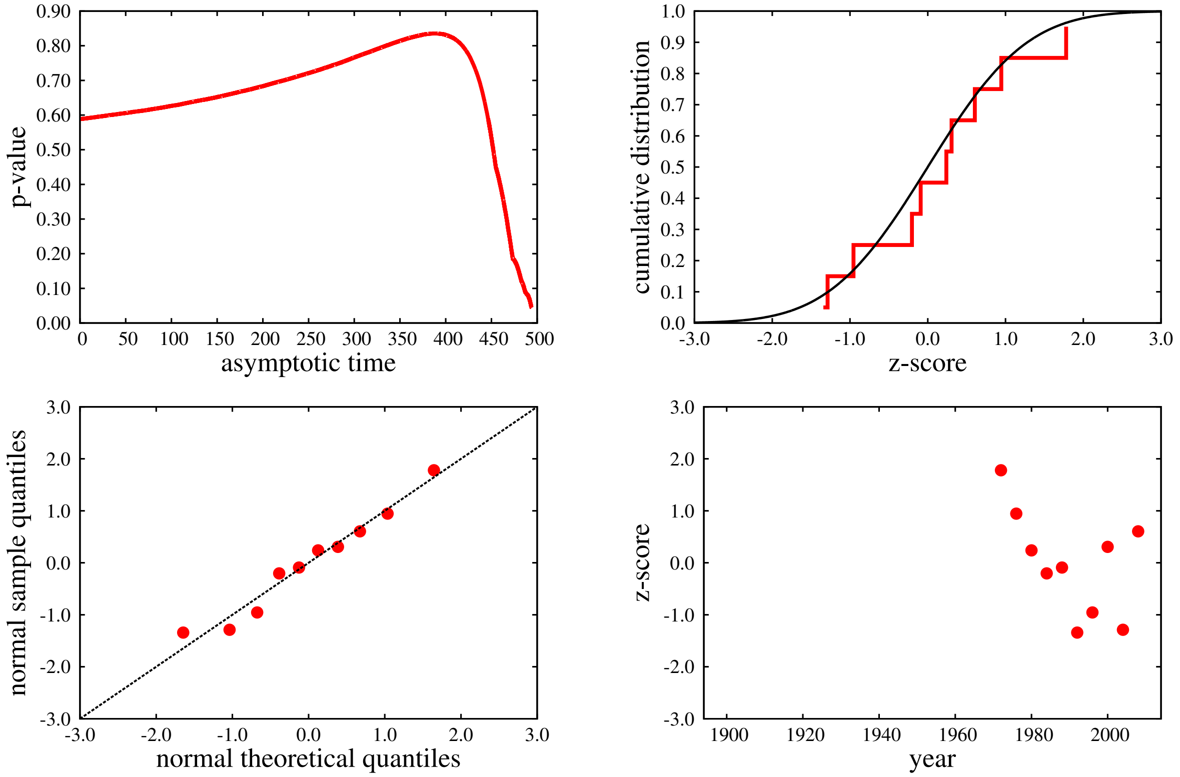

Figure S3.26: Swimming: women 800 meters freestyle

specialty	$\hat{p}_\infty$	$\hat{\mu}$	$\hat{\sigma}$	A^{*2}	p-value	$\hat{p}_\infty^{(u)}$	nr. events
men 100 meters backstroke*	48.98	0.09	0.11	0.20	0.93	52.53	22
men 100 meters breaststroke	57.38	0.16	0.16	0.20	0.93	58.90	11
men 100 meters butterfly*	50.36	0.21	0.29	0.27	0.76	50.57	11
men 100 meters freestyle	44.84	0.09	0.10	0.21	0.92	46.90	23
men 1500 meters freestyle	576.77	0.05	0.05	0.37	0.50	837.68	23
men 200 meters backstroke	113.69	0.24	0.34	0.28	0.74	113.92	13
men 200 meters breaststroke*	122.15	0.10	0.15	0.39	0.46	127.51	23
men 200 meters butterfly	107.24	0.13	0.14	0.15	0.99	112.01	14
men 200 meters freestyle	100.18	0.14	0.15	0.14	0.99	102.94	12
men 4 x 100 meters medley relay	207.67	0.21	0.20	0.33	0.59	209.30	13
men 4 x 200 meters freestyle relay	405.84	0.12	0.13	0.20	0.93	417.97	22
men 400 meters freestyle	163.41	0.05	0.05	0.31	0.64	211.94	23
men 400 meters individual medley	212.73	0.07	0.08	0.18	0.96	243.79	12
women 100 meters backstroke*	54.73	0.08	0.14	0.34	0.59	58.95	20
women 100 meters breaststroke	62.08	0.13	0.10	0.23	0.86	64.84	11
women 100 meters butterfly	52.74	0.09	0.19	0.16	0.98	55.66	14
women 100 meters freestyle	51.87	0.12	0.19	0.36	0.54	53.11	22
women 200 meters backstroke	125.21	0.19	0.44	0.25	0.81	125.22	11
women 200 meters breaststroke	122.64	0.08	0.07	0.49	0.28	138.99	20
women 200 meters butterfly	108.28	0.07	0.11	0.29	0.69	124.16	11
women 200 meters freestyle	114.06	0.19	0.31	0.35	0.54	114.80	11
women 4 x 100 meters freestyle relay	207.48	0.13	0.11	0.20	0.93	213.20	18
women 4 x 100 meters medley relay	218.78	0.11	0.11	0.18	0.96	232.64	13
women 400 meters freestyle	213.55	0.08	0.08	0.35	0.56	235.58	20
women 400 meters individual medley	255.76	0.11	0.18	0.23	0.86	269.40	12
women 800 meters freestyle	388.16	0.05	0.07	0.24	0.84	493.31	11

Table S3.1: Summary table for specialties in Swimming. From left to right, we report: the name of the specialty, the best estimate of the asymptotic performance value $\hat{p}_\infty$, the best estimate of the average value $\hat{\mu}$ and standard deviation $\hat{\sigma}$ of performance improvements, the Anderson-Darling distance A^{*2} between sample and theoretical normal distributions, the statistical significance (p-value) of the normal fit, the upper value at 5% significance level of the limiting performance value $\hat{p}_\infty^{(u)}$, and the number of Olympic games that included the specialty. Unless specified, the values of $\hat{\mu}$, $\hat{\sigma}$, A^{*2} and p-value have been calculated at $p_\infty = \hat{p}_\infty$. Results annotated with * have been obtained by identifying the best estimate of the asymptotic time as a local maximum of the p-value.
